# Key floral-fruity aroma compounds in Sichuan Congou black tea: identification via MDGC-MS/O and sensory evaluation

**DOI:** 10.3389/fnut.2025.1577302

**Published:** 2025-05-02

**Authors:** Sixuan Wang, Luorui Li, Qing Meng, Wei Luo, Fang Wei, Liyong Luo, Liang Zeng

**Affiliations:** ^1^Integrative Science Center of Germplasm Creation in Western China (CHONGQING) Science City, College of Food Science, Southwest University, Chongqing, China; ^2^Chongqing Key Laboratory of Speciality Food Co-Built by Sichuan and Chongqing, Southwest University, Chongqing, China; ^3^Tea Research Institute, Southwest University, Chongqing, China; ^4^Westa College, Southwest University, Chongqing, China

**Keywords:** Sichuan Congou black tea, floral-fruity odor note, MDGC-MS/O, sensory evaluation, addition experiment

## Abstract

Congou black tea has garnered significant interest among customers because of its distinctive floral-fruity aroma. To determine the differences in characteristic volatile compounds among various varieties, a total of 147 volatile compounds were identified across 25 samples via gas chromatography–mass spectrometry (GC-MS). Through hierarchical clustering analysis (HCA), 25 samples were classified into two clusters, in which the Fuding variety, Jinguanyin variety, Huangguanyin variety, and Meizhan variety were clustered into one category, whereas the Sichuan population variety was clustered into the other. These two categories were further analyzed via orthogonal partial least squares discriminant analysis (OPLS-DA), and 48 differential compounds were identified. The Sichuan population variety was found to contain a higher concentration of floral and fruity aroma compounds while exhibiting fewer green or grassy odor compounds. To explore the floral-fruity aroma components of Sichuan Congou black tea (SCBT), forty-nine aroma-active compounds were identified through multidimensional GC–MS/olfactometry (MDGC-MS/O). Among them, six components with odor activity values (OAVs) ≥1 were regarded as key floral-fruity aroma components. Recombination and omission tests ranked the contributions of these compounds as follows: geraniol, linalool, methyl salicylate, benzeneacetaldehyde, beta-myrcene and benzaldehyde. Addition experiments confirmed for the first time that linalool, beta-myrcene and methyl salicylate are the key citrus-like compounds, offering valuable insights for the exploration of germplasm resources and quality control of black tea with special aromas.

## 1 Introduction

Tea a favorable drink that is made from the tender buds and leaves of *Camellia sinensis* L. It is renowned for its taste, nutritional value, and intriguing qualities. Depending on the processing techniques used, fresh tea leaves can be made into various types of finished tea products. Among them, black tea is the most dominate in international trade, accounting for 78% of global tea exports, highlighting its widespread popularity and economic value (1). Black tea is classified into Congou black tea, Souchong black tea and broken black tea (2), one of which, Congou black tea, is an important part of the high-end black tea market. China is a major producer of Congou black tea, offering a diverse range of varieties, such as Sichuan black tea, Yunnan black tea, Keemun black tea, Fujian black tea, and Yingde black tea (3).

Beyond its high concentration of bioactive components, including theaflavins, flavonoids, and tea polysaccharides, Congou black tea is celebrated for its distinctive flavor. However, the aroma profile of Congou black tea varies due to the differences in cultivars, production practices, and processing technics (4). For example, the aromas of Dianhong Congou black tea are sweet, caramel-like, and floral, whereas those of Keemun black tea are honey-like (5, 6). Sichuan Congou black tea (SCBT) is a representative product of the Southwest China tea region, it is distinguished by its unique floral and citrus-like aroma, making it a fascinating subject for flavor chemistry research (7). SCBT is made from tea plant of population variety, propagated from seeds, which undergo gene recombination and mutation over extended periods of natural selection and artificial breeding. This process results in a rich and diverse genetic background, giving rise to teas with complex and layered aroma profiles (8). Among the SCBT produced from the fresh leaves of various Sichuan tea tree varieties, the Sichuan population variety stands out for its exceptional aroma quality, marked by a pronounced floral and fruity profile, making it an exceptional raw material for producing high-fragrance black teas (9, 10). And floral and fruity aroma profiles well meet evolving consumer preferences, emphasizing the importance of analyzing these aroma components. Therefore, it is valuable for understanding aroma formation and evaluating the influence of tea varieties and manufacturing techniques on aroma profile.

To date, more than 600 volatile compounds in black tea have been identified via advanced analytical methods (11). Extensive research has demonstrated that compounds such as linalool, β-ionone, geraniol, β-myrcene, β-cyclocitral, phenethyl alcohol, α-farnesene, (E)-2-hexenal and (Z)-3-hexenyl hexanoate are key components in the development of flowery and fruity aroma (12–15). Traditional artificial sensory evaluation is currently the main approach to define the flavor of tea, but it is difficult to resolve complex odor mixtures and identify trace active compounds (16). To address these challenges, GC-MS has been widely used for the identification and quantitative analysis of tea aroma substances (17, 18). Gas chromatography-olfactometry (GC-O) screening techniques is currently the only method to estimate the sensory contribution of a single odor-active compound in a range of volatile mixtures (19–21). In complex matrices such as tea, high-resolution techniques such as MDGC-MS/O are needed when olfactometry is used to distinguish aroma-active compounds from other components (22). This high-resolution technique not only narrows the range of target analytes but also improves the accuracy of sensory evaluation by reducing matrix interference. It is now widely used in flavor studies of red wine, coffee, and citrus flowers (23–25).

Therefore, the current study was conducted to analyze the floral-fruity compounds of FFBT (floral-fruity aroma Congou black tea). Twenty-five samples with floral-fruity aromas were selected via sensory evaluation from a total of 59 Congou black teas, including both sexual and asexual varieties. The samples were first clustered according to the results of headspace solid-phase microextraction gas chromatography–mass spectrometry (HS-SPME-GC-MS). After screening for the differential metabolites via OPLS-DA, the variable importance in projection (VIP) value analysis was performed to compare and choose the variety with the best floral-fruity scent. The screened aroma-active components were discovered with additional application of MDGC-MS/O. Additionally, OAV analysis was used to filtered out potentially important scent components, which were then confirmed through addition, omission, and recombination tests.

## 2 Materials and methods

### 2.1 Tea samples

Twenty-five samples exhibiting floral-fruity aroma characteristics were selected based on the standard “methodology for sensory evaluation of tea (GB/T 23776-2018)” from fifty-nine Congou black teas in Chongqing in April 2023 (26). The tea sample information and the results of the sensory evaluation are listed in Supplementary Tables S1, S2. Among these twenty-five FFBTs, nine samples were made from the Sichuan population variety (sexual), four samples were made from the Fuding variety (asexual), four samples were made from the Jinguanyin variety (asexual), four samples were made from the Huangguanyin variety (asexual), and four samples were made from the Meizhan (asexual), variety. Additionally, 4 samples from the Sichuan population variety (labeled B1, B2, B3, and B4) presented the most pronounced floral and fruity aromas, with the highest sensory evaluation scores. These samples were chosen for further investigation and stored at −40°C in compound bags lined with aluminum foil.

### 2.2 Chemicals

Sigma-Aldrich (Shanghai, China) supplied a blend of hydrocarbons from heptane to triacontane. We purchased 99% ethyl decanoate from Aladdin Industrial Co. (Shanghai, China). GC-grade ethanol and sodium chloride (NaCl) were acquired from Chongqing Zewu Biotechnology Co., Ltd. (Chongqing, China). A ULUP-II-10T ultrapure water machine (Sichuan ULUPURE Ultrapure Technology Co., Ltd.) was used to produce ultrapure water. Supplementary Table S3 lists the standard scent compounds used for identification verification along with their purity and sources of procurement.

### 2.3 Sensory evaluation of the floral-fruity aroma Congou black tea

#### 2.3.1 Comprehensive analysis and evaluation of aroma factors

The aroma quality of the tea samples was evaluated by 6 experts (3 males and 3 females, aged 20–30 years) with rich experience in sensory review according to the Chinese national standard “Methodology of Sensory Evaluation of Tea” (GB/T 23776, 2018). Three grams of FFBT was infused with 150 mL of boiling water (100°C) for 5 min, after which the liquor was promptly decanted to terminate extraction. The reviewers evaluated the samples by combining hot sniffing (cup temperature of ~75°C), warm sniffing (cup temperature of ~45°C), and cold sniffing (near ambient temperature) and described and scored the samples according to the type, purity, concentration, and persistence of the aroma.

#### 2.3.2 Lexicon development and orientation

An evaluation team of 20 panelists was selected from among tea science students at Southwest University. The brewed tea infusion was transferred into a 40 ml atomizer, which was provided to each panelist for personal sniffing, and the aroma descriptors were recorded. The team leader collected all the descriptors and compiled a flavor wheel. On the basis of this flavor wheel, the panelists further discussed and determined the lexicon of aroma characteristics of the FFBT samples (27). Ultimately, a collection of FFBTs' fragrance phrases, definitions, and references was acquired, and sensory training was carried out on this basis to ensure that the panelists were proficient in identifying the aroma type and intensity to meet the requirements of the QDA.

#### 2.3.3 Quantitative descriptive analysis

The responsibility of evaluating scent qualities and supplying odor intensities fell to a trained panel of ten extremely skilled individuals, five of whom were male and five of whom were female, all of whom were between the ages of twenty and thirty. All the panelists underwent rigorous training using floral-fruity black tea samples and standard aromatic compounds to develop proficiency in recognizing, describing, and discriminating among different aroma qualities. The perceived strength of olfactory characteristics was quantified via a 7-point intensity scale (1 = imperceptible, 4 = moderate, 7 = pronounced). Panel performance was analyzed through PanelCheck 1.4.0 software. Specifically, Tucker-1 multivariate analysis was employed to visualize evaluator consistency patterns, where the spatial proximity of data points reflected stronger panel consensus. High F scores (greater than those corresponding to the 1% and 5% levels of significance) and low MSE values are expected for samples containing differences. In general, the F values and MSE values can be used to evaluate the discriminatory ability and repeatability of evaluators (28, 29). The final sensory scores were derived by computing the average assessments provided by the panelists across the various descriptors. The research was evaluated and approved by the Southwest University IRB (IRB number: HF20240910).

### 2.4 Aroma extraction by HS-SPME

Volatile collection, identification and quantification procedures were performed with slight modifications to the methods of Yu et al. (30). HS-SPME technology was employed to adsorb the scent of tea. The concentration of the internal standard, ethyl decanoate, was 17.26 μg/L, and 0.5 g of tea powder and 2 g of NaCl were added. After adding 5 mL of boiling water and allowing the sample to equilibrate for 5 min, it was maintained at 60°C in a water bath. The SPME fiber (50/30 μm DVB/CAR/PDMS, 1 cm, Supelco, Pennsylvania, USA) were used to adsorb volatile chemicals for 50 min in a headspace container. The fiber was then maintained in a GC injector for 5 min. Each sample was measured in triplicate to ensure robust and reproducible results.

### 2.5 MDGC-MS/O analysis

A 7890B MDGC apparatus (Agilent, Palo Alto, CA, USA) in conjunction with an Agilent 5977B MS and an olfactometric detector (Volatile Analysis Co., Grant, USA) were used to analyze the odorants. Two capillary column systems were used for MDGC separations: an SLOGEL-WAX column (2D, 30 m × 0.53 mm × 0.5 μm) and a BP-5 column (1D, 30 m × 0.53 mm × 0.5 μm). The temperatures of the ion source and transfer line were maintained at 230°C and 280°C, respectively. The scanning range for electron impact (EI) ionization was m/z 33–500, and it was performed at 70 eV. The oven temperature was first set at 35°C, then ramped up by 7°C per minute to 91°C, and then ramped up by 10°C per minute to 201°C. The chemical structures were validated through retrieval from mass spectral libraries (NIST11, W10N14) and matching with authentic standards. Sensory attributes, such as odor quality and intensity, were also considered. A panel of three trained assessors conducted the sensory evaluation via a protocol adapted from prior literature (31). The odor attributes and aroma intensities (AIs) of the separated compounds were measured on a scale from 0 to 100 (0 = absence, 50 = moderate, 100 = strong). The results were averaged across all evaluations, and each sample was examined three times.

### 2.6 GC–MS analysis

The SPME fiber was inserted into the injector of the GC/MS-QP 2020 NX system (Shimadzu Corporation, Kyoto, Japan). The adsorbed compounds were thermally desorbed and then splitlessly injected at an injector temperature of 230°C into a DB-5MS capillary column (30 m × 0.25 mm × 0.25 μm, Shimadzu Corporation, Kyoto, Japan). An initial temperature of 40°C was maintained for 2 min, followed by a ramp of 4°C/min to 100°C, a second ramp of 2°C/min to 120°C, which was sustained for 4 min, and a final ramp of 2.5°C/min to 180°C, which was held for 2 min to achieve chromatographic separation. After 20°C each minute, the oven was raised to 230°C and maintained there for 2 min. The mass scan range was m/z 40–400, the ion source temperature was 230°C, and the ionization energy was 70 eV. The solvent delay time was 5 min.

Volatile chemicals were identified in part via mass spectral libraries (NIST20-1, NIST20-2, and NIST20s). For additional identification, retention indices (RIs) were computed with reference to n-alkanes (C7–C30). The quantification of key odorants was conducted via the use of external standards prepared in ethanol. Calibration curves (*R*^2^ ≥ 99%) were generated to ensure accurate concentration measurements.

### 2.7 Calculation of OAVs

The odor activity value (OAV) is the ratio of a compound's concentration to its odor threshold (OT) in water. Compounds with OAVs ≥1 are thought to contribute to the aroma character of the tea (32). By dividing the chemical concentration in the tea infusion by its OT, each OAV was calculated. The OT represents the minimum concentration perceivable by human olfaction.

### 2.8 Aroma recombination

Both the qualitative and quantitative results of the volatile components in SCBT were validated via recombination analysis. Aroma recombination was conducted via two distinct matrices—pure water and SCBT infusion—that lacked floral and fruity notes, as confirmed through sensory evaluation by well-trained panelists. For reconstitution, all quantitated odorants with OAVs ≥1 were added to each of the two matrices. To create the reconstituted models, standards of these compounds were specifically dissolved in ethanol and introduced into each of the two matrices at the same concentration as the original tea broth. The panelists assessed the scent of the reconstituted models after they had been submerged in a water bath at 60°C for five minutes (33).

### 2.9 Omission tests

In the omission experiment, three samples were labeled with a random code, two of which were complete recombinants containing all the compounds and one was a simplified recombinant missing one compound, and the samples were prepared in the same way as in chapter 2.8 (34). Every odorant with an OAV ≥1 underwent a triangle test. If the sensory panel detected a difference, the flavor as a whole depended on the absent ingredient. Finally, a statistical table was used to establish significance, and the number of correct answers was computed based on the standard “Sensory analysis—Methodology—Triangle test (GB/T 12311-2012)”.

### 2.10 Addition tests

To further verify the extent to which key substances contribute to the floral and fruity aroma attributes of the samples, we conducted addition experiments with slight modifications based on the methods of Yu et al. and Wei et al. (30, 35). Key substances (OAVs ≥ 1) were added to the tea broth lacking floral and fruity aromas on the basis of the average concentration configurations of samples B1, B2, B3, and B4, which were then immediately sealed and blended well. The floral, fruity, and citrus-like attributes of the samples were evaluated by the sensory panelists according to the methodology in Section 2.3.3.

### 2.11 Statistical analysis

All the experiments were conducted in triplicate, and the results were expressed as the means ± standard deviations (SDs). The data were analyzed via ANOVA to determine significant differences between the two samples via IBM SPSS Statistics software (version 27.0, SPSS Inc., Chicago, IL, USA). Panel performance was assessed via PanelCheck 1.4.0 (http://www.panelcheck.com). Hierarchical clustering analysis (HCA) and orthogonal partial least square discriminant analysis (OPLS-DA) were performed via SIMCA 14.1 (Umetrics Corporation, Umeå, Sweden). Heatmaps were generated with TBtools-II (https://github.com/CJ-Chen/TBtools), and figures were created via Origin 8 (OriginLab Co., USA).

## 3 Results and discussion

### 3.1 Lexicon development of floral-fruity aroma Congou black tea samples

The 25 FFBT samples with floral and fruity aroma profile were selected, despite variations in aroma type and intensity. To thoroughly analyze the aroma profiles of FFBT, the panelists individually evaluated each sample, documenting all the perceived aroma attributes. The panel leader consolidated the descriptors and organized them into a flavor wheel (Supplementary Figure S1). This effort resulted in an FFBT lexicon that included 76 aroma descriptors along with their frequencies, which were grouped into 11 categories, with floral and fruity descriptors being the most prevalent. Supplementary Table S4 contains definitions and reference standards for the seven essential phrases that the panelists agreed upon to reflect the sensory aspects of FFBT in the final lexicon on the basis of the descriptive analysis. The panel's performance was subsequently assessed via PanelCheck 1.4.0, confirming its high discriminative ability and reproducibility and meeting the requirements for further analysis (Supplementary Figures S2, S3). To uncover differences in volatile compounds among the FFBT varieties, chemometric analysis was applied to the volatile profiles obtained through GC-MS.

### 3.2 Differences of characteristic volatile compounds in floral-fruity aroma Congou black tea with different varieties

#### 3.2.1 Hierarchical clustering analysis of FFBT

**Figure 1:**
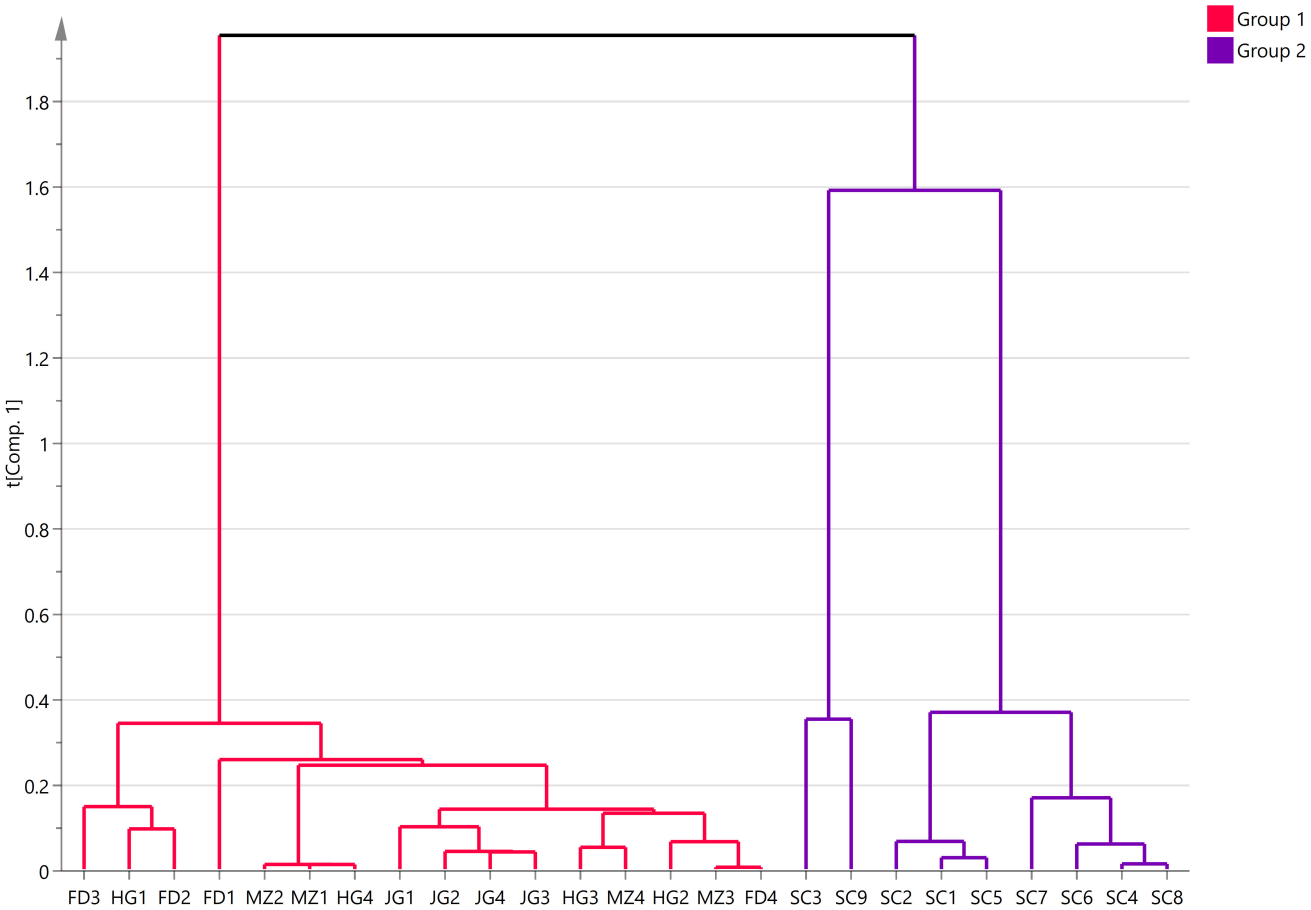
Hierarchical clustering analysis results of 147 compounds in the five varieties floral-fruity aroma Congou black teas in Chongqing.

A total of 147 volatile compounds were identified across the 25 tea samples using GC-MS analysis (Supplementary Table S5), including 30 different types of alcohols, 28 aldehyde compounds, 16 alkenes, 4 alkanes, 20 ketones, 8 acids, 26 esters, and 15 other compounds. The clustering analysis revealed two distinct groups (Figure 1): samples from the Fuding variety, Jinguanyin variety, Huangguanyin variety and Meizhan variety were grouped into group 1, whereas all Sichuan population variety samples were clustered into group 2. In group 1, the Jinguanyin samples formed a distinct cluster, while the other varieties exhibited poor clustering. This may be due to differences in the geographical locations of the tea plantations and variations in processing conditions. Among the different varieties of black tea cultivated in the Chongqing region, the Sichuan population variety presented a unique floral-fruity aroma and was clustered into a separate group. These results align with earlier studies (9).

#### 3.2.2 Candidate differential volatile compounds in FFBT

**Figure 2:**
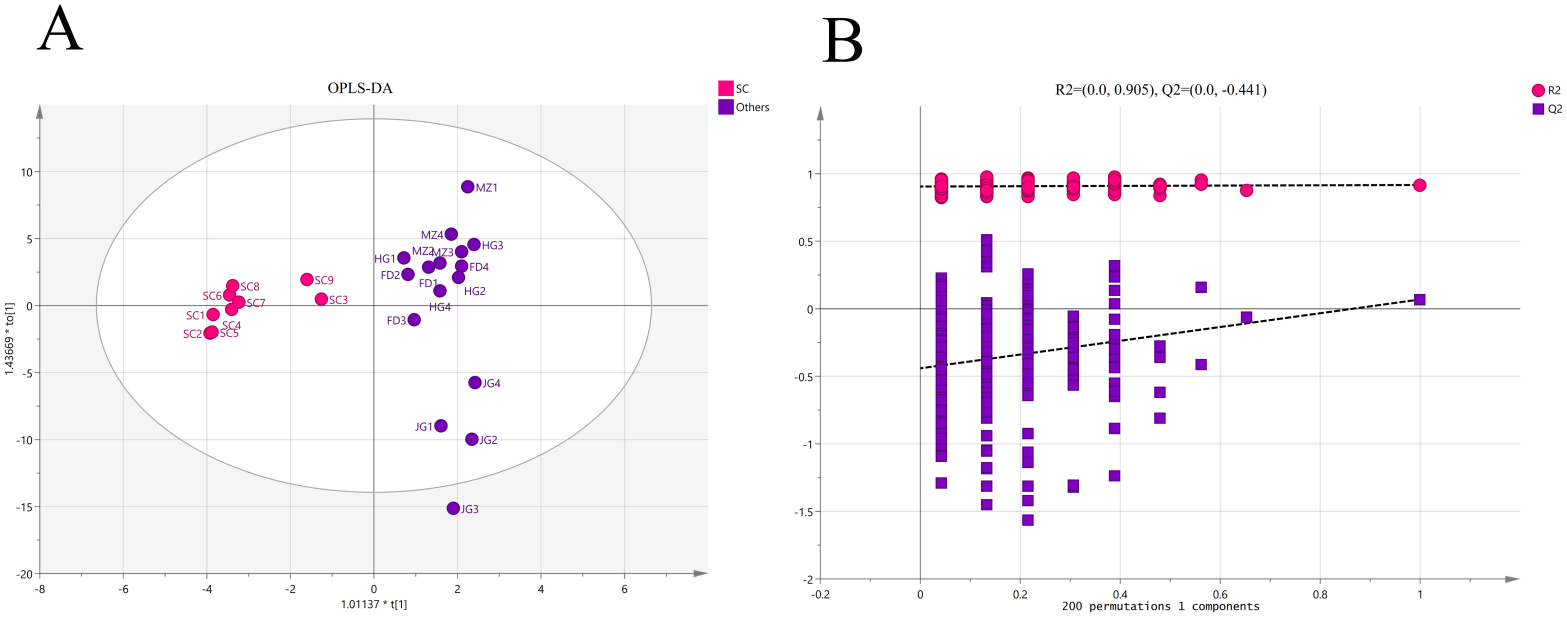
OPLS-DA plot and cross-validation of the five varieties floral-fruity aroma Congou black teas in Chongqing. **(A)** Score plot of OPLS-DA (R2X = 0.508, R2Y = 0.917). **(B)** OPLS-DA validation model using 200 permutation tests (*R*^2^ = 0.905, *Q*^2^ = −0.431).

An OPLS-DA model was developed to identify key volatile markers contributing to class separation between the Sichuan variety and other tea samples, which are displayed in Figure 2A. The cross-validation results are displayed in Figure 2B. The low intercepts (*R*^2^ = 0.905 and *Q*^2^ = −0.431) indicate that the model is not overfit. Supplementary Table S5 shows that 48 compounds had VIP values >1.

**Figure 3:**
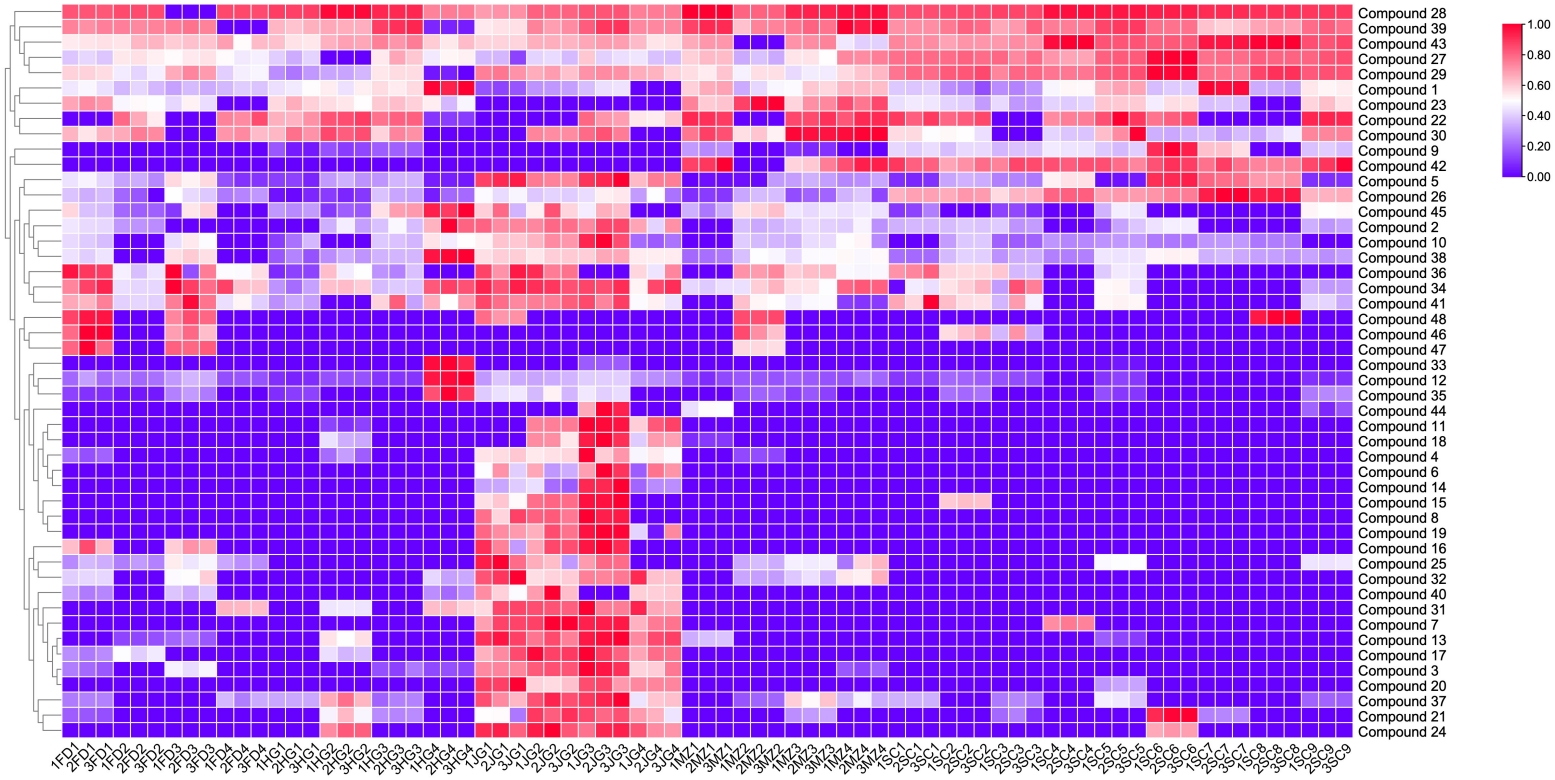
Heatmap results of 48 compounds with a significant difference in the five varieties floral-fruity aroma Congou black teas in Chongqing (VIP value >1 in OPLS-DA). (notes: Compound-1: nerol; Compound-2: (E)-2-octenal; Compound-3: (E,E)-2,4-nonadienal; Compound-4: (E)-2-decenal; Compound-5: (E,E)-2,4-heptadienal; Compound-6: furfural; Compound-7: (E)-3-hexenyl butanoate; Compound-8: (E,E)-2,4-decadienal; Compound-9: nerolidol; Compound-10: hexanal; Compound-11: 2-ethylnitrobenzene; Compound-12: (E)-2-nonenal; Compound-13: benzyl nitrile; Compound-14: 1,2-dihydro-1,1,6-trimethylnaphthalene; Compound-15: (R)-5,6,7,7a-tetrahydro-4,4,7a-trimethyl-2(4H)-benzofuranone; Compound-16: 3-octen-2-one; Compound-17: 2-butyl-2-octenal; Compound-18: (Z)-tetrahydro-6-(2-pentenyl)-2H-pyran-2-one; Compound-19: (E)-β-farnesene; Compound-20: 2-methylpropanoic acid 2-phenylethyl ester; Compound-21: hexyl hexanoate; Compound-22: (E)-citral trans-3,7-dimethylocta-2,6-dienal; Compound-23: α-terpineol; Compound-24: indole; Compound-25: 6-methyl-5-hepten-2-one; Compound-26: benzeneacetaldehyde; Compound-27: benzyl alcohol; Compound-28: geraniol; Compound-29: benzaldehyde; Compound-30: neral; Compound-31: 2,6,6-trimethylcyclohexa-1,3-diene-1-carbaldehyde; Compound-32: (E,Z)-2,6-nonadienal; Compound-33: heptanoic acid; Compound-34: 6,10-dimethyl-5,9-undecadien-2-one; Compound-35: (E)-2-heptenal; Compound-36: α-ionone; Compound-37: cis-3-hexenyl α-methylbutyrate; Compound-38: 3,5-octadien-2-one; Compound-39: trans-linalool oxide (furanoid); Compound-40: (Z)-3-hexen-1-yl benzoate; Compound-41: decanal; Compound-42: β-myrcene; Compound-43: methyl salicylate; Compound-44: 1-hexanol; Compound-45: 1-octen-3-ol; Compound-46: terpinen-4-ol; Compound-47: (E)-1-(2,6,6-trimethylcyclohexa-1,3-dien-1-yl)but-2-en-1-one; Compound-48: citral).

A heatmap was constructed using 48 VIP > 1 compounds to elucidate the aroma characteristics across different varieties (Figure 3). Among these aroma compounds, floral and fruity aroma compounds, such as benzeneacetaldehyde, benzyl alcohol, geraniol and methyl salicylate, were highly abundant in SCBT. In particular, compounds with citrus-like aromas, such as beta-myrcene, nerolidol and (E)-citral trans-3,7-dimethylocta-2,6-dienal, were also present in significant amounts. Each of these factors add to the fruity and floral scent characteristics of SCBTs. Conversely, compounds associated with green or grassy notes, such as (E,E)-2,4-nonadienal, (E)-3-hexenyl butanoate, (E,E)-2,4-decadienal and 3-octen-2-one, were found at lower concentrations in the SCBT samples than in the other samples, which aligns with their cleaner and more refined flavors (36). To uncover the distinct characteristics and significant contributions of the key floral-fruity aroma compounds within the Sichuan population variety, we carried out a sensomics-assisted in-depth characterization of SCBT.

### 3.3 Sensomics-assisted characterization of key floral-fruity aroma compounds in SCBT

#### 3.3.1 Aroma profile analysis of SCBT samples

**Figure 4:**
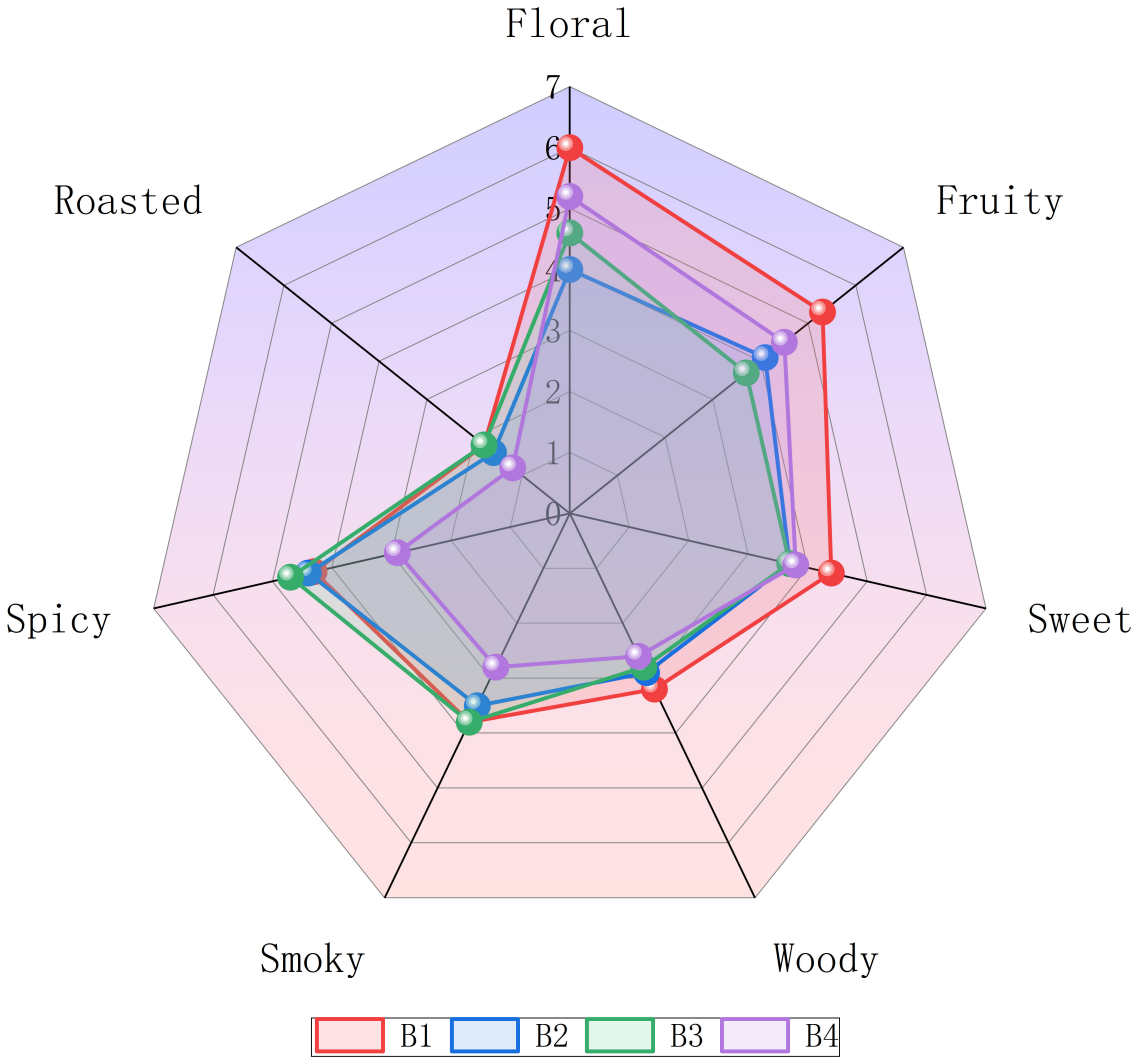
Aroma profiles of Sichuan Congou black tea.

Quantitative descriptive analysis (QDA) revealed seven volatile attributes for the four SCBT samples. The results of the panel test verified that floral aroma (4.0-6.0), fruity aroma (3.7-5.3), and sweet aroma (3.7-4.4) were significant odor attributes, followed by spicy aroma (2.9-4.7), smoky aroma (2.8-3.8), roasted aroma (1.2-1.8), and woody aroma (2.6-3.2). Floral and fruity aromas were the most prominent and consistent aromas across all the samples (Figure 4). In addition, for comprehensive SCBT flavor information, a better combination of sensory and instrumental analysis is needed. Therefore, we identified aroma-active compounds through MDGC-MS/O.

#### 3.3.2 Aroma-active compounds identified by MDGC-MS/O

Among the four samples, 46 aroma-active chemicals, including 9 alcohols, 6 esters, 6 ketones, 3 acids, 4 phenols, 1 sulfide, 1 nitrile compound, 2 heterocyclic compounds, 4 alkenes and 10 aldehydes, were detected together with the characteristics and intensities of their odors (Table 1). All four tea samples included 11 aroma-active chemicals, namely, β-ionone (81-88), linalool (73-90), benzeneacetaldehyde (59-93), and geraniol (56-91), which exhibit relatively high aroma intensities, and β-ionone, linalool, and geraniol are all terpenoid compounds. Because they are easier to perceive and have lower odor thresholds, these chemicals are the main sources of the scent of tea (37). The primary active ingredients that give Congou black tea its floral scent are terpenoids (38). Hexanal (44-74), Jasmone (53-63), and 2,4-di-tert-butylphenol (39-77) have moderate aroma intensities, and methyl salicylate (20-68), benzaldehyde (15-41), and β-myrcene (10-31) have slightly lower aroma intensities. Only two tea samples contained β-damascenone, but because of its incredibly low threshold, it was the strongest aroma-active compound (86–92). These 11 aroma-active compounds predominantly exhibited floral, fruity, and sweet characteristics during olfaction, showing diverse floral attributes, such as orchid-like, honeysuckle-like, osmanthus-like, rose-like, and fruity attributes, including citrus-like, apple-like, and peach-like attributes. Notably, floral and fruity descriptors appeared with the highest frequency compared with other vocabularies in all odor quality descriptions, indicating that floral and fruity aromas are the predominant flavor characteristics in the SCBT and revealing a rich diversity of floral and fruity profiles.

**Table 1:** Identification and intensity of aroma-active compounds in SCBT identified by MDGC-MS/O.

**No.^a^**	**CAS**	**Odorants**	**Odor quality^b^**	**Identification method^c^**	**Intensity**			
					**B1**	**B2**	**B3**	**B4**
1	123-72-8	Butanal	Sweety	MS, AD	–^d^	–	64	–
2	590-86-3	Isovaleraldehyde	Sweety	MS, AD	–	80	–	–
3	96-17-3	2-methylbutyraldehyde	Floral	MS, AD	–	18	–	–
4	107-47-1	Tert-butyl sulfide	Oil-Like	MS, AD	–	20	–	–
5	66-25-1	Hexanal	Green, apple-like, pungent	MS, AD	53	70	74	44
6	4914-91-4	cis-3,4-dimethyl-2-pentene	Green	MS, AD	–	46	–	–
7	10493-98-8	2-hydroxycyclopent-2-en-1-one	Unpleasant	MS, AD	–	-	30	–
8	505-57-7	2-hexenal	Roasty	MS, AD	–	27	24	–
9	928-97-2	trans-3-hexen-1-ol	Apple-Like	MS, AD	–	–	–	–
10	123-35-3	beta-myrcene	Woody, citrus-like, milk-like, nutty, roasty	MS, AD, STD	31	10	13	25
11	67-51-6	3,5-dimethylpyrazole	Green, floral	MS, AD	–	79	–	–
12	110-93-0	6-methyl-5-hepten-2-one	Sweety, roasty	MS, AD, STD	88	81	–	–
13	5989-27-5	(+)-dipentene	Green, citrus-like, hawthorn, sweety, potato-like, pungent	MS, AD, STD	58	–	71	51
14	3391-86-4	1-octen-3-ol	Sweety	MS, AD, STD	–	18	–	–
15	4313 03 5	(E,E)-2,4-heptadienal	Sweety	MS, AD, STD	–	43	19	–
16	100-52-7	benzaldehyde	Floral, sweety, herbal, woody	MS, AD, STD	17	19	41	15
17	5989-33-3	Cis-alpha, alpha,5-trimethyl-5-vinyltetrahydrofuran-2-methanol	Unpleasant	MS, AD	–	–	17	–
18	10117-03-0	Ethyl 2-(5-methyl-5-vinyltetrahydrofuran-2-yl)propan-2-yl carbonate	Chocolate-like, orchid, peach, herbal, roasty	MS, AD	67	65	25	53
19	30086-02-3	3,5-octadien-2-one	Apple-like	MS, AD	–	–	58	–
20	78-70-6	Linalool	Orchid, citrus-like, honeysuckle, pungent	MS, AD, STD	83	90	73	77
21	2835-96-3	4-amino-2-methylphenol	Honeysuckle, jasmine	MS, AD	–	90	–	77
22	122-78-1	Benzeneacetaldehyde	Stamen, orchid, green, osmanthus, rose	MS, AD, STD	85	93	59	78
23	29957-43-5	3,7-dimethylocta-1,5,7-trien-3-ol	Orchid, honeysuckle, green, rose, fruity	MS, AD	76	59	–	88
24	1786-08-9	Neryl oxide	Roasty, apple-like, honeysuckle, fatty	MS, AD	77	–	50	–
25	142-62-1	Hexanoic acid	Bean-like, milk-like	MS, AD	–	40	–	–
26	100-51-6	Benzyl alcohol	Floral, roasty, pungent, smoky, grape-like	MS, AD	–	45	66	47
27	586-63-0	3-methyl-6-(1-methylethylidene)cyclohexene	Unpleasant	MS, AD	–	60	–	–
28	119-36-8	Methyl salicylate	Green, nutty, floral, herbal	MS, AD, STD	45	20	63	68
29	60 12 8	Phenylethyl alcohol	Orchid, rose	MS, AD	–	90	–	88
30	106-25-2	Nerol	Rose, pungent, fruity, leather-like	MS, AD, STD	86	–	81	–
31	5392-40-5	Citral	Osmanthus, green, grape-like, rose	MS, AD	54	48	30	–
32	2142-94-1	Neryl formate	Rose	MS, AD	–	–	–	90
33	140-29-4	Benzyl nitrile	Floral	MS, AD	–	29	–	–
34	106-24-1	Geraniol	Rose, honeysuckle, orchid, woody, fruity	MS, AD, STD	87	91	63	56
35	624-15-7	3,7-dimethyl-2,6-octadien-1-ol	Floral, sweety	MS, AD	41	75	–	–
36	4411-89-6	2-phenyl-2-butenal	Unpleasant, fatty	MS, AD	–	–	29	55
37	23726-93-4	Beta-damascenone	Jujube, sweety, honeysuckle	MS, AD	92	86	–	–
38	488-10-8	Jasmone	Caramel-like, fatty, herbal, floral	MS, AD, STD	53	60	63	63
39	85-91-6	Benzoic acid, 2-(methylamino)-, methyl ester	Acidity, fruity	MS, AD	–	–	30	–
40	79-77-6	β-lonone	Osmanthus, citrus-like, honeysuckle	MS, AD	82	88	79	81
41	4698 08 2	(2E)-3,7-dimethyl-2,6-octadienoic acid	Osmanthus	MS, AD	–	41	–	–
42	134-20-3	Methyl anthranilate	Grape-like, floral	MS, AD	40	–	86	–
43	6846-50-0	2,2,4-trimethyl-1,3-pentanediol diisobutyrate	Rose	MS, AD, STD	41	–	–	–
44	40716-66-3	Nerolidol	Milk-like, tobacco	MS, AD	–	62	40	–
45	459-80-3	Geranic acid	Milk-like, tobacco	MS, AD	–	90	–	–
46	96-76-4	2,4-di-tert-butylphenol	Sweety, floral, milk-like, herbal	MS, AD	73	77	45	39

a Compounds are shown according to their order of appearance in the chromatogram on the BP-5 and SLOGEL-WAX columns.

b The odor quality of each odorant was described as the panelists sensed at the sniffing port.

c MS, mass spectra; AD, aroma descriptor; STD, authentic reference compounds.

d Not detected at the sniffing port.

Furthermore, linalool, β-ionone and β-myrcene, which are compounds with citrus aroma, were detected in all the four SCBTs. These substances have relatively high scent intensities and are thought to be the main aroma-active components in mandarins (39–41). Additionally, in the fruity odor quality assessment by SCBT, citrus had the highest occurrence frequency, suggesting that the fruity characteristics of SCBT can be further interpreted as a citrus-like aroma. These findings are consistent with those of previous studies; SCBT has a characteristic citrus-like aroma, but the key substances responsible for presenting this citrus-like aroma still need to be further verified. However, more quantification of these compounds is required in order to accurately assess the contribution of these aroma-active compounds and take into account the effect of the matrix on aroma release.

#### 3.3.3 Quantitation of volatiles and calculation of OAVs

**Figure 5:**
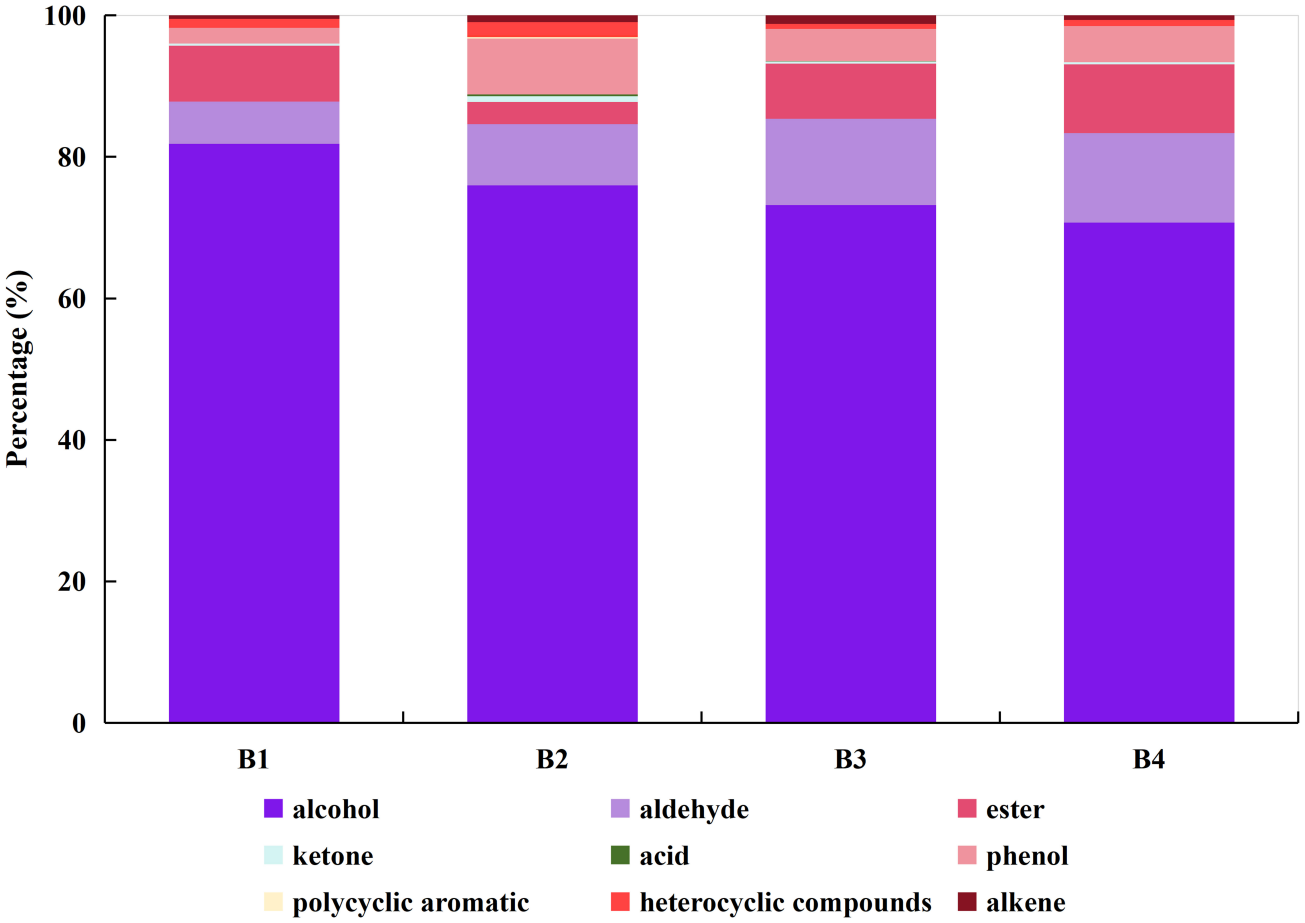
Constitution of volatile compounds in SCBT obtained with HS-SPME-GC–MS (B1, B2, B3, and B4 denote the four SCBT samples with the most distinctive floral and fruity aroma characteristics selected through sensory evaluation). The types of volatile compounds represented by different colors are as follows: (1) dark purple for alcohol, (2) light purple for aldehyde, (3) rose for ester, (4) blue for ketone, (5) green for acid, (6) pink for phenol, (7) yellow for polycyclic aromatic, (8) bright red for heterocyclic and (9) dark red for alkene.

GC-MS was used to examine volatile chemicals both qualitatively and quantitatively. Eleven alcohols, six esters, two ketones, one acid, three phenols, one polycyclic aromatic molecule, four heterocyclic compounds, three alkenes, and twelve aldehydes were detected in the four SCBT samples (Table 2). The content of each compound category varied among the four samples, but the differences in the proportions of the total content were not significant (Figure 5). According to the results of the sensory evaluation, the comparable scent profiles of the four SCBTs may be explained by the comparable proportions of different constituent groups (42).

**Table 2:** Identification, concentrations, OTs and OAVs (scores ≥ 1) of volatile compounds determined by GC–MS in SCBT.

**No**.	**RI^a^**	**Compounds**	**Identification basis^b^**	**Content (ug/L)^c^**				**OT (ug/L)^d^**	**OAV^e^**			
				**B1**	**B2**	**B3**	**B4**		**B1**	**B2**	**B3**	**B4**
1	1249	Geraniol	RI, MS, STD	1,903.63 ± 2.82	1,534.12 ± 9.94	1,247.32 ± 28.91	1,161.53 ± 14.99	6.60	288.4	232.4	188.9	176.0
2	1100	Linalool	RI, MS, STD	39.69 ± 0.42	26.35 ± 1.31	116.85 ± 1.71	46.72 ± 3.11	0.22	180.4	119.8	531.2	212.4
3	1045	Benzeneacetaldehyde	RI, MS, STD	81.23 ± 1.23	64.41 ± 3.79	168.55 ± 6.78	140.55 ± 1.08	4.00	20.3	16.1	42.1	35.1
4	994	Beta-myrcene	RI, MS, STD	11.41 ± 0.27	12.18 ± 0.46	9.11 ± 3	8.18 ± 0.49	1.20	9.5	10.1	7.6	6.8
5	1190	Methyl salicylate	RI, MS, STD	185.94 ± 10.865	73.81 ± 5.22	151.06 ± 22.79	185.56 ± 3.71	40.00	4.6	1.8	3.8	4.6
6	964	Benzaldehyde	RI, MS, STD	40.68 ± 1.68	100.51 ± 1.68	40.42 ± 0.3	70.93 ± 0.62	24.00	1.7	4.2	1.7	3.0
7	1071	Cis-alpha, alpha,5-trimethyl-5-vinyltetrahydrofuran-2-methanol	RI, MS	14.94 ± 0.52	n.d.^f^	n.d.	n.d.	6.00	2.5	n. a.	n. a.	n. a.
8	804	Hexanal	RI, MS, STD	1.47 ± 0.14	1.26 ± 0.85	1.85 ± 0.69	1.34 ± 0.33	4.50	<1	<1	<1	<1
9	853	2-hexenal	RI, MS	1.85 ± 0.08	1.06 ± 0.21	1.58 ± 0.13	3.02 ± 0.63	40.00	<1	<1	<1	<1
10	855	(E)-hex-3-en-1-ol	RI, MS	1.15 ± 0.06	1.35 ± 0.71	0.86 ± 0.18	0.96 ± 0.2	110.00	<1	<1	<1	<1
11	1014	(E,E)-2,4-heptadienal	RI, MS, STD	8.27 ± 0.21	36.69 ± 9.06	20.84 ± 8.53	15.59 ± 4.57	15.40	<1	2.4	1.4	1.0
12	1035	Benzyl alcohol	RI, MS, STD	24.95 ± 0.91	119.74 ± 34.22	43.36 ± 10.59	49.86 ± 6.47	100.00	<1	1.2	<1	<1
13	1047	1-ethyl-1 h-pyrrole-2-carbaldehyde	RI, MS	7.23 ± 0.65	24.2 ± 15.48	n.d.	n.d.	2,000.00	<1	<1	n. a.	n. a.
14	1063	2-acetyl pyrrole	RI, MS, STD	3.21 ± 0.09	5.65 ± 0.89	2.65 ± 0.64	n.d.	1,00000.00	<1	<1	<1	n. a.
15	1087	Trans-Linalool oxide (furanoid)	RI, MS, STD	20.61 ± 1.43	22.68 ± 3.9	11.37 ± 3.36	15.89 ± 1.77	190.00	<1	<1	<1	<1
16	1103	3,7-dimethylocta-1,5,7-trien-3-ol	RI, MS	11.26 ± 0.77	28.32 ± 0.72	3.35 ± 0.19	4.08 ± 0.59	1,100.00	<1	<1	<1	<1
17	1110	Phenylethyl alcohol	RI, MS, STD	28.01 ± 0.49	84.36 ± 26.54	40.93 ± 7.82	48.27 ± 2.91	60.00	<1	1.4	<1	<1
18	1184	Cis-3-hexenyl butyrate	RI, MS	0.95 ± 0.29	n.d.	n.d.	n.d.	500.00	<1	n. a.	n. a.	n. a.
19	1194	Alpha-terpineol	RI, MS, STD	1.81 ± 0.09	3.31 ± 1.41	1.62 ± 0.05	n.d.	86.00	<1	<1	<1	n. a.
20	1223	Nerol	RI, MS, STD	6.16 ± 0.19	3.57 ± 0.39	40.33 ± 6.83	2.49 ± 0.22	49.00	<1	<1	<1	<1
21	1236	(Z)-3,7-dimethylocta-2,6-dienal	RI, MS	1.59 ± 0.01	1.37 ± 0.26	1.46 ± 0.26	1.73 ± 0.71	3.00	<1	<1	<1	<1
22	1266	(E)-citral trans-3,7-dimethylocta-2,6-dienal	RI, MS	10.87 ± 0.21	15.55 ± 3.92	n.d.	n.d.	32.00	<1	<1	n. a.	n. a.
23	1376	Cis-3-hexenyl hexanoate	RI, MS, STD	3.72 ± 0.3	n.d.	n.d.	n.d.	781.00	<1	n. a.	n. a.	n. a.
24	1387	Jasmone	RI, MS, STD	5.37 ± 0.11	13.51 ± 0.4	3.21 ± 1.41	2.79 ± 0.43	24.00	<1	<1	<1	<1
25	1504	2,4-di-tert-butylphenol	RI, MS	1.92 ± 1.18	7.94 ± 5.77	3.3 ± 1	n.d.	500.00	<1	<1	<1	n. a.
26	1560	Nerolidol	RI, MS, STD	1.53 ± 0.06	6.27 ± 1.67	2.67 ± 0.21	n.d.	250.00	<1	<1	<1	n. a.
27	734	Pentanal	RI, MS	n.d.	n.d.	0.72 ± 0.21	n.d.	200.00	n. a.	n. a.	<1	n. a.
28	903	Heptanal	RI, MS	n.d.	n.d.	0.54 ± 0.18	n.d.	31.00	n. a.	n. a.	<1	n. a.
29	981	Hexanoic acid	RI, MS, STD	n.d.	6.32 ± 3.88	0.69 ± 0.28	n.d.	80,000.00	n. a.	<1	<1	n. a.
30	1060	(E)-2-octenal	RI, MS	n.d.	2.32 ± 0.49	n.d.	n.d.	0.34	n. a.	6.8	n. a.	n. a.
31	1094	3,5-octadien-2-one	RI, MS	1.92 ± 0.05	8.63 ± 1.18	3.19 ± 1	2.84 ± 0.85	n. f.	n. a.	n. a.	n. a.	n. a.
32	1136	(E,Z)-alloocimene	RI, MS	0.71 ± 0.42	n.d.	n.d.	n.d.	n. f.	n. a.	n. a.	n. a.	n. a.
33	1167	(3R,6S)-2,2,6-trimethyl-6-vinyltetrahydro-2H-pyran-3-ol	RI, MS	32.29 ± 0.19	24.86 ± 4.83	5.68 ± 1.23	7.94 ± 0.88	n. f.	n. a.	n. a.	n. a.	n. a.
34	1181	Naphthalene	RI, MS	n.d.	6.11 ± 2.2	n.d.	n.d.	6.00	n. a.	1.0	n. a.	n. a.
35	1213	2,4-dimethylbenzaldehyde	RI, MS	n.d.	8.45 ± 0.71	n.d.	n.d.	n. f.	n. a.	n. a.	n. a.	n. a.
36	1266	Citral	RI, MS, STD	n.d.	n.d.	n.d.	8.84 ± 0.69	28.00	n. a.	n. a.	n. a.	<1
37	1287	Indole	RI, MS, STD	n.d.	3.31 ± 1.6	n.d.	n.d.	11.00	n. a.	<1	n. a.	n. a.
38	1419	Beta-cedrene	RI, MS	n.d.	13.92 ± 5.34	14.15 ± 0.71	4.22 ± 0.5	n. f.	n. a.	n. a.	n. a.	n. a.
39	1484	Creamy lactone	RI, MS	1.09 ± 0.3	7.89 ± 3.2	n.d.	n.d.	n. f.	n. a.	n. a.	n. a.	n. a.
40	1585	2,2,4-trimethyl-1,3-pentanediol diisobutyrate	RI, MS	2.91 ± 0.07	n.d.	n.d.	n.d.	n. f.	n. a.	n. a.	n. a.	n. a.
41	1599	Cedrol	RI, MS, STD	n.d.	406.96 ± 53.68	423.91 ± 6.36	126.92 ± 14.75	n. f.	n. a.	n. a.	n. a.	n. a.
42	1639	Methyl jasmonate	RI, MS	n.d.	2.67 ± 0.71	n.d.	n.d.	13.00	n. a.	<1	n. a.	n. a.
43	1649	Alpha-cadinol	RI, MS	n.d.	3.46 ± 0.25	n.d.	n.d.	n. f.	n. a.	n. a.	n. a.	n. a.

a The RI of the odorants was calculated via a mixture of n-alkane series (C7–C30).

b RI, retention index; MS, mass spectra; STD, authentic reference compounds.

c Values were expressed as “average concentration ± SD”, each included 3–6 replicates.

d OT, odor threshold; The OTs were taken from a book titled “Compilations of odor threshold values in air, water and other media” (49) and determined (nos. 7, 8, 12, 16, 17, 20, 24 and 26) according to references (21, 46, 50–52).

e Odor activity values were calculated by dividing the average concentration by the OTs in water.

f n.d., not detectable; n.f., not yet found; n.a., not available owing to lack of quantitative data.

Notably, 22 of these compounds were consistent with those detected by MDGC-MS/O. Interestingly, compounds such as β-lonone, hexanal, and 2,4-di-tert-butylphenol, which presented relatively high intensities in the MDGC-MS/O analysis, were not detected. These overlaps may lead to unclear mass spectrometry library matches, particularly with 1D GC separation (43). For the analysis of MDGC-MS/O, multidimensional separation was carried out via gas chromatographic columns with different polarities (BP-5 column and SLOGEL-WAX column), achieving good separation of aromatic isomers with good resolution and enabling more accurate determination results to be obtained.

Therefore, the quantification of volatile compounds is crucial for assessing the influences of these compounds on the SCBT. Standard curves were established, and precise quantification analysis was carried out using the associated standard substances for important chemicals with OAVs ≥ 1 (Supplementary Table S6). Among these compounds, geraniol presented the highest concentration in SCBT, ranging from 1,161.53 to 1,903.63 μg/L, which was tenfold greater than the concentrations of the other compounds. Methyl salicylate (73.81–185.94 μg/L), benzeneacetaldehyde (64.41–168.55 μg/L), benzaldehyde (40.42–100.51 μg/L), and linalool (26.35–116.85 μg/L) were the second most abundant compounds. Notably, cedrol (126.92–423.91 μg/L) exhibited high concentrations in the three tea samples but was not detected in the B1 sample.

**Figure 6:**
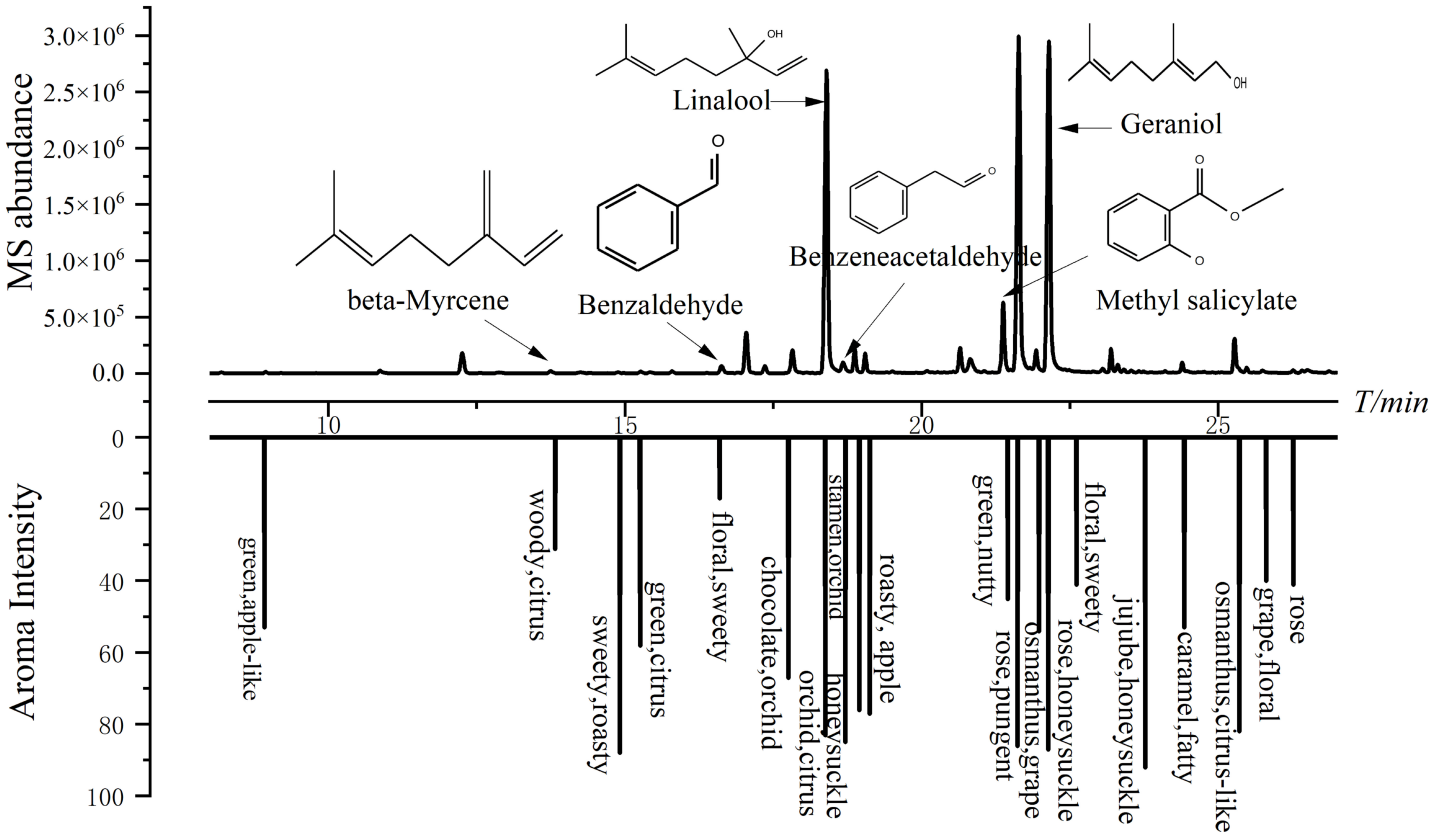
MDGC-MS and MDGC-O chromatograms (**upper**) and aroma intensity (AI) chromatograms (**lower**) of Sichuan Congou black tea.

Since some odorants have very low OTs, they contribute considerably to the overall aroma even at low concentrations. Hence, odor activity values (OAVs) were calculated to provide a more nuanced assessment of the role of odorants in tea infusions. Across all four tea samples, six odorants presented OAVs ≥ 1 (Table 2). Geraniol (176.0–288.4) and linalool (119.8–531.2) had the highest OAVs, followed by benzeneacetaldehyde (20.3–42.1), beta-myrcene (6.8–10.1), methyl salicylate (1.8–4.6), and benzaldehyde (1.7–4.2). In certain tea samples, six additional odorants were present at OAVs ≥ 1, including cis-alpha, alpha,5-trimethyl-5-vinyltetrahydrofuran-2-methanol (2.5), (E,E)-2,4-heptadienal (1.0–2.4), benzyl alcohol (1.2), phenylethyl alcohol (1.4), (E)-2-octenal (6.8), and naphthalene (1.0). Comparatively, six substances with OAVs ≥ 1 across all four tea samples were also detected and found to have high intensity via MDGC-MS/O analysis (Figure 6). Therefore, it is inferred that these six compounds constitute the key aroma-active compounds in SCBT. Furthermore, through panel testing, it was verified via MDGC-MS/O that all six aroma-active compounds exhibit floral and fruity aroma characteristics. By excluding and adding odorants with OAVs ≥ 1, the impact of certain odorants on the intensity of the floral-fruity aroma was further elucidated.

#### 3.3.4 Verification of key floral-fruity odorants of SCBT

**Figure 7:**
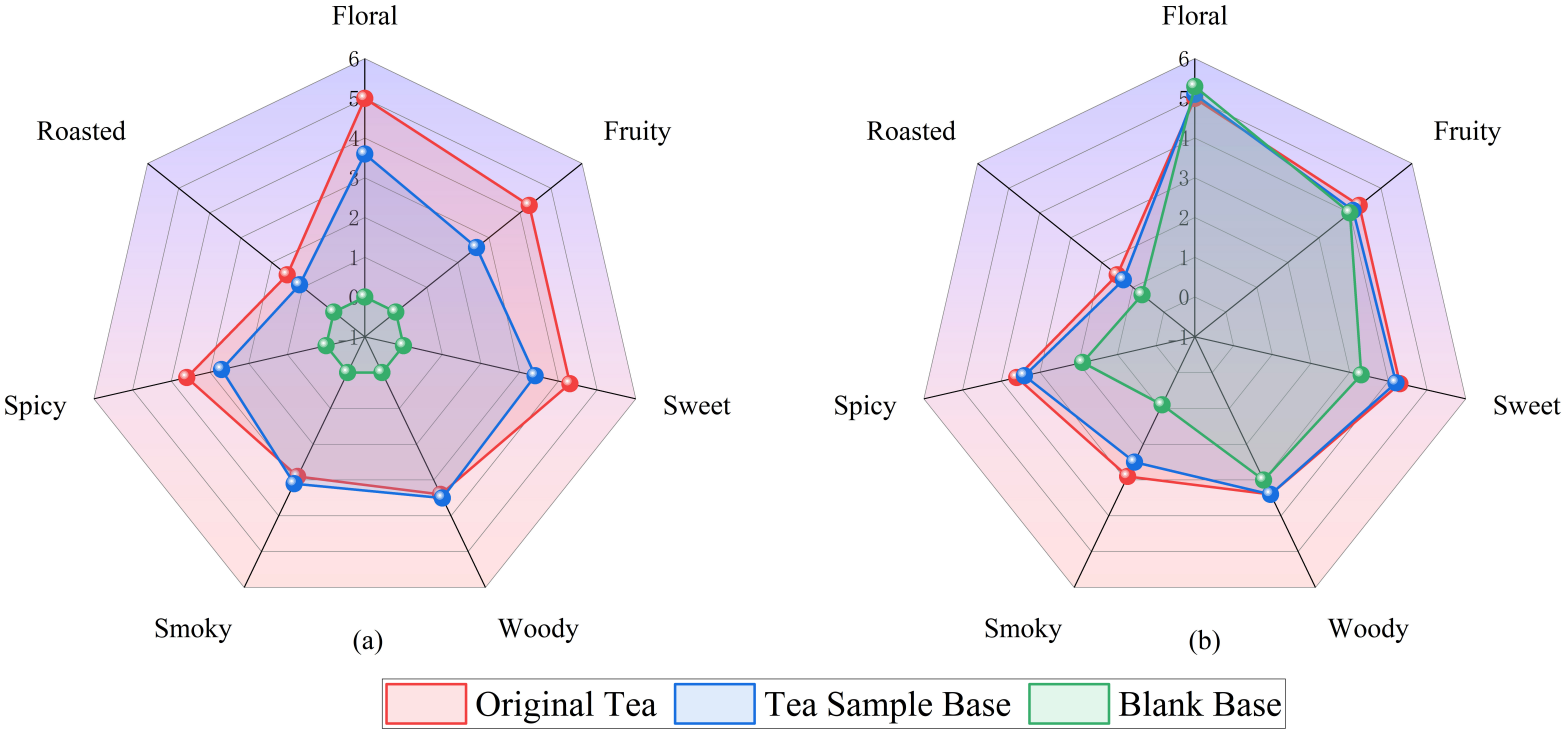
**(a)** Two bases for the recombinant models: a blank base (green line) and a tea sample base (blue line). **(b)** Sichuan Congou black tea infusion (red line) and two aroma recombination models in a water bath at 60°C (green line, blue line) are compared for their scent profiles.

Reconstitution experiments were carried out to verify whether the identified and quantified odorants significantly contributed to the overall aroma of SCBT by replicating the aromatic profile of the infusion. Considering that these six compounds predominantly exhibit floral, fruity, and sweet aroma attributes, two reconstitution bases were established: (1) a blank base and (2) a base tea sample selected by the expert panel, which shares similar flavor characteristics with the original tea infusion except for floral, fruity, and sweet aroma attributes (Figure 7a). The reconstitution results using the blank matrix demonstrated that the addition of the six compounds effectively represented floral and fruity attributes, albeit with some deficiencies in sweet, woody, roasted, spicy, and smoky aroma characteristics. These six compounds are the primary factors contributing to the floral and fruity scent profile of the SCBT, as further supported by the base tea reconstitution's overall similarity score of 2.8 (out of 3), which successfully represented all of the important aromas found in the SCBT (Figure 7b). The aromas of Keemun, Guizhou, Yunnan, and Hunan black teas are also largely attributed to these constituents (44–48).

Omission studies were conducted to assess the influence of key aroma-active compounds on the overall olfactory profile. These tests involved sniffing individual models in which each of the six compounds was omitted from the reconstitution experiment. The experimental results are summarized in Table 3. According to the table for the correct answers and the number of evaluators required for the three-point test, all the test results show sensory differences, demonstrating the significant impact of the six omitted compounds on the overall aroma of the SCBT. According to the omission experiments, the contributions of these six odorants from high to low are as follows: geraniol, linalool, methyl salicylate, benzeneacetaldehyde, beta-myrcene and benzaldehyde.

**Table 3:** Number of correct answers and p-values in omission tests.

**Test**	**Components**	**Correct ratio^a^**	**Statistical significance**
1	Benzaldehyde	15/24	*p =* 0.01
2	Linalool	17/24	*p* < 0.001
3	Methyl salicylate	16/24	*p =* 0.001
4	Geraniol	20/24	*p* < 0.001
5	Benzeneacetaldehyde	16/24	*p =* 0.001
6	Beta-myrcene	16/24	*p =* 0.001

a The correct ratio is expressed as “correct answers/participated panelists”.

We performed addition experiments to better investigate the roles of these key compounds in floral and fruity scent qualities (Table 4). The results demonstrated that linalool, geraniol and methyl salicylate contribute to both floral and fruity aromas. Benzaldehyde, benzeneacetaldehyde and beta-myrcene contribute to the fruity aroma. The contributions of these key fruity aroma compounds to the citrus-like aroma of SCBT were specifically analyzed. The results indicated that linalool, beta-myrcene and methyl salicylate are the key citrus-like compounds in SCBT. Interestingly, although methyl salicylate does not have a citrus-like aroma, it effectively enhances the citrus-like aroma of SCBT. It might interact with other substances, strengthening the citrus-like aroma attributes and modifying and coordinating the overall floral and fruity aroma effects of SCBT.

**Table 4:** Group assignment and result of adding floral and fruity odorants in SCBT.

**Group^a^**	**Odorants**	**Intensity of floral odor note^b^**	**Intensity of fruity odor note**	**Intensity of citrus-like odor note**	**Variance of floral odor note (%)^c^**	**Variance of fruity odor note (%)**	**Variance of citrus-like odor note (%)**
Base tea	SCBT that lacked floral and fruity notes	3.6^c^^b^	2.6^c^	0^c^	–	–	–
1	Base tea + benzaldehyde	3.46^c^^d^	3.14^c^^b^	0^c^	−3.89	20.77	–
2	Base tea + linalool	4.71^a^	3.86^a^^b^	1.79^a^	30.83	48.46	–
3	Base tea + methyl salicylate	4.37^a^^b^	3.86^a^^b^	1.07^b^	21.39	48.46	–
4	Base tea + geraniol	4.97^a^	2.96^c^	0^c^	38.06	13.85	–
5	Base tea + benzeneacetaldehyde	3.21^c^^d^	2.93^c^	0^c^	−10.83	12.69	–
6	Base tea + beta-myrcene	2.64^d^	3.21^c^^b^	1.71^a^	−26.67	23.46	–
7	Base tea + benzaldehyde, linalool, methyl salicylate, geraniol, benzeneacetaldehyde and beta-myrcene	5.14^a^	4.13^a^	2.21^a^	42.78	58.85	–

a The base tea used in the addition experiment is a tea sample with very low intensities of floral and fruity odors, which was selected by the expert panel.

b The intensities were expressed as the average score, and the values followed by the same letter in the same column represented not significantly different (p > 0.05).

c The variance was the rate of the difference between the added group and the odor intensity of the base tea.

## 4 Conclusion

Floral-fruity aroma Congou black tea (FFBT) has recently attracted consumer interest due to its distinctive flavor. Among the 59 Congou black teas from Chongqing, 25 samples with floral-fruity aromas were identified through sensory evaluation, resulting in the creation of a comprehensive FFBT lexicon comprising 76 descriptors. To investigate the differences in characteristic volatile compounds among the FFBT varieties, 147 volatile compounds were identified via HS-SPME-GC-MS. Cluster analysis grouped the 25 samples into two distinct clusters, in which the Fuding variety, Jinguanyin variety, Huangguanyin variety and Meizhan variety were clustered into one category, whereas the Sichuan population variety was clustered into the other.

These two categories were further analyzed via OPLS-DA, which identified 48 differential compounds (VIP values greater than 1) between the two groups. Among them, the Sichuan population variety had relatively high levels of differential compounds exhibiting floral and fruity aromas and relatively low levels of differential compounds exhibiting green or grassy odors, further highlighting the superior quality of Sichuan Congou black tea (SCBT).

To explore the floral-fruity aroma components of SCBT, four tea samples from the Sichuan population variety, selected for their high sensory scores and prominent floral-fruity aroma characteristics, were subjected to detailed analysis. MDGC-MS/O identified 49 aroma-active compounds, among which six with OAVs ≥ 1 were deemed key contributors to the floral-fruity aroma of SCBT. Recombination and omission tests ranked their contributions as follows: geraniol, linalool, methyl salicylate, benzeneacetaldehyde, β-myrcene, and benzaldehyde. Addition experiments further revealed that linalool, geraniol, and methyl salicylate contribute to both floral and fruity notes, while benzaldehyde, benzeneacetaldehyde, and β-myrcene primarily enhance fruity aromas. Notably, linalool, β-myrcene, and methyl salicylate were identified as key citrus-like compounds in SCBT for the first time.

Although this study successfully identified the aroma-differentiating compounds between sexual and asexual tea varieties and determined the key floral-fruity aroma components in SCBT, further research is necessary to understand the transformation of these compounds during processing and elucidate the aroma formation mechanisms using multi-omics approaches. Future studies will focus on identifying the key enzymes involved in the biosynthesis of floral-fruity aroma compounds during processing, unraveling the mechanisms behind flower and fruit aroma formation induced by mechanical damage, and providing scientific guidance for improving and precisely regulating the aroma quality of SCBT.

## Data Availability

The original contributions presented in the study are included in the article/Supplementary material, further inquiries can be directed to the corresponding author/s.
