# Supplementary material for: Key floral-fruity aroma compounds in Sichuan Congou black tea: identification via MDGC-MS/O and sensory evaluation
Source: Front Nutr. 2025 May 2;12:1577302. doi: 10.3389/fnut.2025.1577302 (PMC12083010; doi:10.3389/fnut.2025.1577302)
Supplement: Supplementary file 1 [file Table_1.docx]

***Supplementary Material***

**Table S1. The Information of Tea Samples in Study.**

| **No.^ab^** | **Manufacturer** | **Name** | **Origin** | **SC Number** |
| --- | --- | --- | --- | --- |
| 1 | Wuxi Xinming Tea Industry Co., Ltd | Gangshang black tea | Wuxi County | SC11450023851010 |
| 2 | Chongqing Shanyu Yixiang Tea Co., Ltd | Shanyu Danxia | Banan District | SC11450011332861 |
| 3 | Chongqing Dexuanju Tea Industry Co., Ltd | Forest wild tea (Kui Hong) | Kaizhou District | SC11450015450238 |
| 4 | Chongqing Dexuanju Tea Industry Co., Ltd | Forest wild tea (Jin Hong) | Kaizhou District | SC11450015450238 |
| 5 | Tianliang Agricultural Development Co., Ltd | Nanchuan Black Tea | Nanchuan District | SC11450011950215 |
| 6 | Chongqing Yuniao Forestry Co., Ltd | Longxi Congou | Wanzhou District | SC11450010131807 |
| 7 | Chongqing Shajingshan Tea Industry Co., Ltd | Nanchuan Black Tea | Nanchuan District | SC11450011937558 |
| 8 | Chongqing Shajingshan Tea Industry Co., Ltd | Hongyun Congou | Nanchuan District | SC11450011937558 |
| 9 | Chongqing Guorui Tea Industry Co., Ltd | Youyang Black Tea 1 | Youyang County | SC11450024230425 |
| 10 | Chongqing Guorui Tea Industry Co., Ltd | Youyang Black Tea 2 | Youyang County | SC11450024230425 |
| 11 | Chongqing Xishi Gai Tea Industry Co., Ltd | Xiushan black tea | Xiushan County | SC11450024132354 |
| 12 | Chongqing Xiushan Zhongling Tea Industry Co., Ltd | Zhongling Gongou Black Tea | Xiushan County | SC11450024112630 |
| 13 | Chongqing Junzhiyuan Agricultural Development Co., Ltd | Yanshan black tea | Wanzhou District | SC11450010120884 |
| 14 | Chongqing Border Town Yun Tea Industry Co., Ltd | Xiushan black tea | Xiushan County | SC11450024132354 |
| 15 | Chongqing Longcangxiang Dashu Tea Co., Ltd. Cooperative | Jinfoshan Guqiao Black Tea | Nanchuan District | SC11450011937558 |
| 16 | Chongqing Chengkou Jiming Tea Industry Co., Ltd | Jiming Black Tea | Chengkou County | SC11450022904175 |
| 17 | Xiushan County Yuanshui Agricultural Development Co., Ltd | Biancheng Gongou Black Tea | Xiushan County | SC11450024131763 |
| 18 | Chongqing Huangmingyuan Agricultural Comprehensive Development Co., Ltd | Xiushan black tea | Xiushan County | SC11450024103836 |
| 19 | Xiushan Jiaming Tea Industry Co., Ltd | Xiushan black tea | Xiushan County | SC11450024150167 |
| 20 | Xiushan Jiawo Agricultural Development Co., Ltd | Xiushan black tea | Xiushan County | SC11450024138605 |
| 21 | Xiushan County Minrui Agriculture Co., Ltd | Xiushan black tea | Xiushan County | SC11450024134397 |
| 22 | Chongqing Shuinan Tea Industry Co., Ltd | Shui Nan Xiang | Yongchuan District | SC11453090200159 |
| 23 | Chongqing Longding Tea Industry Co., Ltd | High Mountain Black Tea | Wuxi County | - |
| 24 | Chongqing Yunling Tea Industry Technology Co., Ltd | Yunling Gongou Black Tea | Yongchuan District | SC11450011826650 |
| 25 | Chongqing Yonghu Tea Industry Co., Ltd | Sanxia Black Tea | Wanzhou District | SC11450010132859 |
| 26 | Chongqing Mushu Agricultural Development Co., Ltd | China Red No. 4 | Wulong District | SC11450023214242 |
| 27 | Chongqing Mushu Agricultural Development Co., Ltd | China Red Tripod Charm | Wulong District | SC11450023214242 |
| 28 | Chongqing Yonghu Tea Industry Co., Ltd | Sanxia Black Tea | Wanzhou District | SC11450010132859 |
| 29 | Chongqing Mushu Agricultural Development Co., Ltd | China Red No. 7 | Wulong District | SC11450023214242 |
| 30 | Chongqing Mushu Agricultural Development Co., Ltd | China Red No. 6 | Wulong District | SC11450023214242 |
| 31 | Chongqing Mushu Agricultural Development Co., Ltd | China Red No. 5 | Wulong District | SC11450023214242 |
| 32 | Chongqing Mushu Agricultural Development Co., Ltd | China Red No.1 | Wulong District | SC11450023214242 |
| 33 | Chongqing Mushu Agricultural Development Co., Ltd | China Red No.2 | Wulong District | SC11450023214242 |
| 34 | Yangzhao Tea Processing Factory in Jiangjin District | Bar shaped black tea | Jiangjin District | - |
| 35 | Chongqing Mushu Agricultural Development Co., Ltd | China Red No. 3 | Wulong District | SC11450023214242 |
| 36 | Chongqing Chayou Agricultural Development Co., Ltd | Number 5 | Wulong District | SC11450023229496 |
| 37 | Chongqing Chiming Agriculture Co., Ltd | Wonderland | Wulong District | SC11450023217337 |
| 38 | Chongqing Chiming Agriculture Co., Ltd | Ultimate Realm | Wulong District | SC11450023217337 |
| 39 | Chongqing Yimu Agricultural Development Co., Ltd | Conglao | Wulong District | SC11450023236397 |
| 40 | Xiushan Kaibao Tea Industry Co., Ltd | Kaibao black tea | Xiushan County | SC11450024121899 |
| 41 | Chongqing Tea Industry (Group) Co., Ltd | Bayuhong | Banan District | SC11450011320962 |
| 42 | Chongqing Jinshanhu Agricultural Development Co., Ltd | Ancient Tree Black Tea | Nanchuan District | SC11450011930808 |
| 43 | Xiushan County Dagang Tea Industry Co., Ltd | Guanyin | Xiushan County | SC11450024150222 |
| 44 | Chongqing Jinjiang Ecological Agriculture Co., Ltd | Shizhu black tea | Shizhu County | SC11450024050008 |
| 45 | Chongqing Yinghe Sizhen Tea Co., Ltd | Wen Hong | Jiangjin District | SC11450011650122 |
| 46 | Chongqing Cidi Huahua Tea Culture Communication Co., Ltd | Yanshan Red | Wanzhou District | SC11450010131807 |
| 47 | Xiushan County Jianshan Agricultural Comprehensive Development Co., Ltd | Congou black tea | Xiushan County | SC11450024134565 |
| 48 | Chongqing Dazu District Gulong Tea Co., Ltd | Shengshou Yunhua | Dazu District | SC11450022517931 |
| 49 | Chongqing Wuwen Tea Industry Co., Ltd | Chenjin black tea | Nanchuan District | SC11450011913037 |
| 50 | Chongqing Guorui Tea Industry Co., Ltd | Youyang Black Tea | Youyang County | SC11450024230425 |
| 51 | Chongqing Guorui Tea Industry Co., Ltd | Youyang Black Tea | Youyang County | SC11450024230425 |
| 52 | Chongqing Guorui Tea Industry Co., Ltd | Youyang Black Tea | Youyang County | SC11450024230425 |
| 53 | Chongqing Guorui Tea Industry Co., Ltd | Youyang Black Tea | Youyang County | SC11450024230425 |
| 54 | Xiushan County Minrui Agriculture Co., Ltd | Xiushan black tea | Xiushan County | SC11450024134397 |
| 55 | Xiushan County Minrui Agriculture Co., Ltd | Xiushan black tea | Xiushan County | SC11450024134397 |
| 56 | Xiushan County Minrui Agriculture Co., Ltd | Xiushan black tea | Xiushan County | SC11450024134397 |
| 57 | Xiushan County Minrui Agriculture Co., Ltd | Xiushan black tea | Xiushan County | SC11450024134397 |
| 58 | Chongqing Yonghu Tea Industry Co., Ltd | Sanxia Black Tea | Wanzhou District | SC11450010132859 |
| 59 | Chongqing Yonghu Tea Industry Co., Ltd | Sanxia Black Tea | Wanzhou District | SC11450010132859 |

^a^ Sichuan population variety: SC1, SC2, SC3, SC4, SC5, SC6, SC7, SC8, and SC9 correspond to tea samples No. 19, 29, 31, 34, 36, 46, 48, 58, and 59, respectively. Fuding variety: FD1, FD2, FD3 and FD4 correspond to tea samples No. 35, 54, 55, and 56, respectively. Jinguanyin variety: JG1, JG2, JG3 and JG4 correspond to tea samples 50, 51, 52 and 53 respectively. Huangguanyin variety: HG1, HG2, HG3 and HG4 correspond to tea samples No. 9, 21, 25, and 41, respectively. Meizhan variety: MZ1, MZ2, MZ3 and MZ4 correspond to tea samples No. 22, 33, 40 and 57 tea samples, respectively.

^b^ Samples B1, B2, B3 and B4 correspond to samples SC4, SC6, SC7 and SC8, respectively.

**Table S2. The results of sensory evaluation of fifty-nine Congou black teas.**

| **No.** | **Aroma Comments ^a^** | **Aroma Score ^b^** |
| --- | --- | --- |
| 1 | Pure and normal aroma | 80.8±1 |
| 2 | Strong and lasting sweet potato aroma | 86.2±0.8 |
| 3 | Sweet potato aroma | 85.5±0.5 |
| 4 | High-fired aroma | 78.7±0.8 |
| 5 | Pure and normal aroma | 81±1 |
| 6 | Sweet potato aroma with grass odor | 74±1 |
| 7 | Sweet potato aroma | 84.7±0.6 |
| 8 | Pure and normal aroma | 81.2±1.3 |
| 9 | Pronounced sweet aroma with a hinted floral nuance | 90.5±0.5 |
| 10 | Sweet potato aroma | 85±1 |
| 11 | Pure and normal aroma | 80.8±1 |
| 12 | Sweet potato aroma | 84.5±0.5 |
| 13 | Pure and normal aroma | 80.5±0.5 |
| 14 | Pure and normal aroma | 81.5±1.3 |
| 15 | Sweet potato aroma | 84.2±0.8 |
| 16 | Pure and normal aroma | 81±1 |
| 17 | Sweet potato aroma, little brunt | 70.3±0.6 |
| 18 | Sweet, not persistent | 85.8±1 |
| 19 | Sweet with fruity notes | 91±1 |
| 20 | Sweet potato aroma, not persistent | 84.5±1.3 |
| 21 | Sweet with floral notes | 91.2±1 |
| 22 | High and sharp sweet orange aroma, little milky | 91.8±0.8 |
| 23 | Sweet potato aroma, little brunt | 70.5±0.5 |
| 24 | Sweet potato aroma | 84.5±0.5 |
| 25 | Sweet and fragrant with a hint of floral aroma | 91.5±0.5 |
| 26 | Sweet potato aroma | 84.8±0.8 |
| 27 | Pure and normal aroma | 80.7±1.2 |
| 28 | Sweet potato aroma | 85±1 |
| 29 | Sweet and little floral | 91.3±1.2 |
| 30 | High-fired aroma, little brunt | 71.3±1.5 |
| 31 | Sweet aroma, high-fired aroma, little floral aroma | 84.3±0.6 |
| 32 | Strong and lasting sweet potato aroma | 87±1 |
| 33 | Sweet and floral, long lasting | 93.5±0.5 |
| 34 | Fruity and floral, long lasting | 98.3±0.6 |
| 35 | Sweet and fruity, persistent | 91.3±1.5 |
| 36 | Sweet and little floral, with a cool sensation | 91.3±0.6 |
| 37 | Sweet potato aroma | 84.2±0.8 |
| 38 | Sweet potato aroma | 84.2±1.9 |
| 39 | Sweet potato aroma | 83.8±1 |
| 40 | Sweet potato aroma is dominant, little floral | 89.7±0.6 |
| 41 | Sweet potato aroma is dominant, little floral | 87.7±0.6 |
| 42 | Sweet and strong aroma | 88±1 |
| 43 | Pure and normal aroma | 81.3±1.5 |
| 44 | Sweet potato aroma is strong and persistent | 87.7±0.6 |
| 45 | Sweet potato aroma | 85.5±0.9 |
| 46 | Floral and fruity aroma, long lasting | 96.5±0.5 |
| 47 | Sweet potato aroma | 84.2±0.8 |
| 48 | Floral and fruity with sweet notes | 97.2±0.8 |
| 49 | Pure and normal aroma | 80.8±0.8 |
| 50 | Little floral and fruity, not persistent | 90.5±0.5 |
| 51 | Floral and fruity aroma | 92±1 |
| 52 | Floral and fruity aroma is sweet and long | 94.5±0.5 |
| 53 | Sweet, floral and fruity aroma | 92.7±0.6 |
| 54 | Sweet with caramelized aroma, little floral | 88.5±0.5 |
| 55 | Strong and lasting floral and fruity aroma | 91±1 |
| 56 | Sweet, little floral, little grass odor | 85±1 |
| 57 | Floral and fruity aroma little strong and lasting | 92.8±0.8 |
| 58 | Floral, fruity, sweet and persistent | 97.8±0.8 |
| 59 | Strong and lasting caramelized, little floral aroma | 90.2±0.3 |

^a^ Twenty-five samples (No.9, 19, 21, 22, 25, 29, 31, 33, 34, 35, 36, 40, 41, 46, 48, 50, 51, 52, 53, 54, 55, 56, 57, 58 and 59) exhibiting floral-fruity aroma characteristics were selected based on the standard “methodology for sensory evaluation of tea (GB/T 23776-2018)” from fifty-nine Congou black teas in Chongqing in April 2023.

^b^ Four samples from the Sichuan population variety No.34, 46, 48 and 58 (labeled B1, B2, B3, and B4) demonstrated the most pronounced floral and fruity aroma, achieving the highest sensory evaluation scores.

**Table S3. The Information of Reference Aroma Compounds Used in Experiment.**

| **no.^a^** | **Compound^b^** | **Supplier** |
| --- | --- | --- |
| 1 | geraniol | Macklin Biochem (Shanghai, China) |
| 2 | geranyl formate | Macklin Biochem (Shanghai, China) |
| 3 | cis-3-hexenl butyrate | Macklin Biochem (Shanghai, China) |
| 4 | 2-phenylethanol | Macklin Biochem (Shanghai, China) |
| 5 | hexanal | Macklin Biochem (Shanghai, China) |
| 6 | benzaldehyde | Macklin Biochem (Shanghai, China) |
| 7 | hexanoic acid | Macklin Biochem (Shanghai, China) |
| 8 | cis-jasmone | Macklin Biochem (Shanghai, China) |
| 9 | cis-3-hexenyl hexanoate | Macklin Biochem (Shanghai, China) |
| 10 | alpha-terpineol | Macklin Biochem (Shanghai, China) |
| 11 | 1-ethyl-1h-pyrrole-2-carboxaldehyde | Macklin Biochem (Shanghai, China) |
| 12 | trans,trans-2,4-heptandienal | Macklin Biochem (Shanghai, China) |
| 13 | indole | Macklin Biochem (Shanghai, China) |
| 14 | 2,2,4-trimethyl-1,3-pentanediol  diisobutyrate | Macklin Biochem (Shanghai, China) |
| 15 | beta-myrcene | Macklin Biochem (Shanghai, China) |
| 16 | 6-methyl-5-hepten-2-one | Macklin Biochem (Shanghai, China) |
| 17 | 1-octen-3-ol | Macklin Biochem (Shanghai, China) |
| 18 | linalool | Macklin Biochem (Shanghai, China) |
| 19 | benzeneacetaldehyde | Macklin Biochem (Shanghai, China) |
| 20 | methyl salicylate | Macklin Biochem (Shanghai, China) |
| 21 | cedrol | TCI Chemical (Shanghai, China) |
| 22 | citral(cis-and trans-mixture) | TCI Chemical (Shanghai, China) |
| 23 | trans-2-hexenal | TCI Chemical (Shanghai, China) |
| 24 | nerol | TCI Chemical (Shanghai, China) |
| 25 | (+)-dipentene | Rhawn (Shanghai, China) |
| 26 | benzyl alcohol | Aladdin Industrial (Shanghai, China) |
| 27 | nerolidol | Yuanye Bio-Technology (Shanghai, China) |
| 28 | trans-linalool oxide (furanoid) | Yuanye Bio-Technology (Shanghai, China) |

**Table S4. Definition and References of Sichuan Congou Black Tea Sensory Profiles.**

| **No.** | **Description** | **Definition** | **References (0 = none, 7 = very)** |
| --- | --- | --- | --- |
| 1 | roasted | Warm aroma roasted at high temperatures | Sliced toasted buns = 2 |
| 2 | sweet | Sweet aroma which evokes impression of sugar | Sweet potato chips = 5 |
| 3 | spicy | An irritating effect on the nasal cavity and a slight spicy sensation | Cinnamon = 7 |
| 4 | fruity | A fruity, sweet, and slightly volatile fragrance that resembles the scent of ripe pears and apples | 0.3×10^−3^ g/L ethyl hexanoate = 7 |
| 5 | smoky | Smoky aroma of burnt woody, or like a smell of an object that has been smoked | Lapsong souchong = 5 |
| 6 | woody | Aromatics associated with dry wood or wood by-products | 0.01 g/L Cedrol = 4 |
| 7 | floral | Intense, fresh and sweet perfume impression associated with flowers | 0.559×10^−3^ g/L geranyl formate = 7 |
| 8 | citrus-like | A harmonious combination of sweet, tangy citrus notes with a touch of warmth and softness | Fresh oranges = 7 |

**Table S5. Identification, concentrations and VIP (scores ≥ 1) of volatile compounds determined by GC‒MS in floral-fruity aroma congou black tea samples.**

| **No.** | | **RI** | **CAS** | **Compounds** | **Types** | **Identification basis** | **VIP** | **Content (ug/L)** | | | | | | | |
| --- | --- | --- | --- | --- | --- | --- | --- | --- | --- | --- | --- | --- | --- | --- | --- |
|  |  |  |  |  |  |  |  | **FD1** | **FD2** | **FD3** | **FD4** | **HG1** | **HG2** | **HG3** | **HG4** |
| 1 | 698 | 64-19-7 | Acetic acid | Acids | RI, MS | <1 | n.d. | n.d. | n.d. | n.d. | n.d. | n.d. | n.d. | n.d. |  |
| 2 | 744 | 110-05-4 | Di-tert-butyl peroxide | Others | RI, MS | <1 | n.d. | n.d. | n.d. | n.d. | n.d. | 0.66±0.12 | n.d. | n.d. |  |
| 3 | 801 | 66-25-1 | Hexanal | Aldehydes | RI, MS | 1.3359 | 3±0.37 | n.d. | 4.53±1.05 | n.d. | 1.39±1.01 | n.d. | 2.68±0.25 | 4.85±0.92 |  |
| 4 | 828 | 98-01-1 | Furfural | Aldehydes | RI, MS | 1.18407 | n.d. | n.d. | n.d. | n.d. | n.d. | n.d. | n.d. | n.d. |  |
| 5 | 845 | 505-57-7 | 2-Hexenal | Aldehydes | RI, MS | <1 | n.d. | n.d. | n.d. | 3.72±1.14 | n.d. | 2.55±2.2 | 1.75±0.41 | 0.88±0.22 |  |
| 6 | 850 | 6728-26-3 | (E)-2-Hexenal | Aldehydes | RI, MS | <1 | n.d. | n.d. | n.d. | n.d. | n.d. | n.d. | n.d. | n.d. |  |
| 7 | 852 | 33467-74-2 | (Z)-3-Hexen-1-ol formate | Alcohols | RI, MS | <1 | 3.94±0.23 | n.d. | n.d. | n.d. | n.d. | n.d. | n.d. | n.d. |  |
| 8 | 853 | 928-96-1 | (E)-3-Hexen-1-ol | Alcohols | RI, MS | <1 | n.d. | n.d. | n.d. | n.d. | n.d. | n.d. | 6.08±0.12 | n.d. |  |
| 9 | 863 | 928-95-0 | (E)-2-Hexen-1-ol | Alcohols | RI, MS | <1 | n.d. | n.d. | n.d. | n.d. | n.d. | n.d. | 0.51±0.37 | n.d. |  |
| 10 | 867 | 111-27-3 | 1-Hexanol | Ketones | RI, MS | 1.12692 | n.d. | n.d. | n.d. | n.d. | n.d. | n.d. | n.d. | n.d. |  |
| 11 | 889 | 110-43-0 | 2-Heptanone | Ketones | RI, MS | <1 | n.d. | n.d. | 1.97±0.86 | n.d. | n.d. | n.d. | n.d. | n.d. |  |
| 12 | 901 | 111-71-7 | Heptanal | Aldehydes | RI, MS | <1 | n.d. | n.d. | n.d. | n.d. | 0.79±0.21 | n.d. | n.d. | 0.89±0.03 |  |
| 13 | 909 | 142-83-6 | (E,E)-2,4-Hexadienal | Aldehydes | RI, MS | <1 | n.d. | n.d. | n.d. | n.d. | n.d. | n.d. | n.d. | n.d. |  |
| 14 | 909 | 3208-16-0 | 2-Ethylfuran | Others | RI, MS | <1 | n.d. | n.d. | n.d. | n.d. | n.d. | n.d. | n.d. | n.d. |  |
| 15 | 918 | 100-51-6 | Nerolidol | Alkenes | RI, MS | 1.07399 | n.d. | n.d. | n.d. | n.d. | 0.22±0.18 | 0.33±0.06 | 0.47±0.02 | n.d. |  |
| 16 | 927 | 110-13-4 | 2,5-Hexanedione | Ketones | RI, MS | <1 | n.d. | n.d. | n.d. | n.d. | n.d. | n.d. | n.d. | n.d. |  |
| 17 | 954 | 18829-55-5 | (E)-2-Heptenal | Others | RI, MS | 1.21947 | 0.27±0.06 | 0.11±0.01 | 0.81±0.14 | n.d. | 0.29±0.06 | n.d. | n.d. | 6.79±1.42 |  |
| 18 | 958 | 100-52-7 | Benzaldehyde | Esters | RI, MS | 1.10782 | 19±0.99 | 10.79±1.31 | 31.24±4.98 | 10.94±1.81 | 4.79±0.75 | 6.45±0.58 | 17.46±0.95 | 1.28±0.29 |  |
| 19 | 970 | 111-70-6 | 1-Heptanol | Alcohols | RI, MS | <1 | 0.37±0.07 | n.d. | 1.57±0.88 | n.d. | n.d. | n.d. | n.d. | 1.67±0.86 |  |
| 20 | 976 | 4312-99-6 | 1-Octen-3-one | Ketones | RI, MS | <1 | n.d. | n.d. | n.d. | n.d. | n.d. | n.d. | n.d. | n.d. |  |
| 21 | 979 | 3391-86-4 | 1-Octen-3-ol | Alkenes | RI, MS | 1.25923 | 1.38±0.58 | 0.45±0.03 | 1.41±1.03 | n.d. | 0.75±0.06 | 0.35±0.1 | 2.51±0.5 | 5.12±0.69 |  |
| 22 | 983 | 110-93-0 | 6-Methyl-5-hepten-2-one | Aldehydes | RI, MS | 1.14005 | 1.04±0.21 | 0.8±0.12 | 1.92±0.29 | 0.9±0.13 | n.d. | n.d. | n.d. | n.d. |  |
| 23 | 989 | 123-35-3 | β-Myrcene | Ketones | RI, MS | 1.11163 | n.d. | n.d. | n.d. | n.d. | n.d. | n.d. | n.d. | n.d. |  |
| 24 | 995 | 4313-03-5 | (E,E)-2,4-Heptadienal | Aldehydes | RI, MS | 1.12057 | 5.13±0.65 | 2.69±0.49 | 8.27±0.94 | 0.84±0.22 | 0.76±0.2 | 2.75±0.44 | 3.06±0.52 | 0.46±0.04 |  |
| 25 | 1002 | 124-13-0 | Octanal | Aldehydes | RI, MS | <1 | 0.54±0.07 | 0.17±0.06 | n.d. | n.d. | 0.45±0.02 | 0.16±0.1 | n.d. | 8.52±0.12 |  |
| 26 | 1024 | 99-87-6 | p-Cymene | Alkenes | RI, MS | <1 | n.d. | n.d. | n.d. | n.d. | 0.7±0.13 | n.d. | n.d. | n.d. |  |
| 27 | 1029 | 5989-27-5 | D-Limonene | Alkenes | RI, MS | <1 | n.d. | n.d. | n.d. | n.d. | n.d. | 3.37±1.37 | n.d. | n.d. |  |
| 28 | 1032 | 100-51-6 | Benzyl alcohol | Aldehydes | RI, MS | 1.34865 | 5.78±0.64 | 12.73±0.99 | 12.14±1.51 | 9±1.3 | 5.41±1.18 | n.d. | 18.26±6.11 | 10.26±1.95 |  |
| 29 | 1037 | 3779-61-1 | trans-β-Ocimene | Alkenes | RI, MS | <1 | n.d. | n.d. | n.d. | n.d. | n.d. | n.d. | n.d. | n.d. |  |
| 30 | 1037 | 1669-44-9 | 3-Octen-2-one | Others | RI, MS | 1.10109 | 2.12±0.65 | n.d. | 1.89±0.27 | n.d. | n.d. | n.d. | n.d. | n.d. |  |
| 31 | 1042 | 122-78-1 | benzeneacetaldehyde | Aldehydes | RI, MS | 1.07435 | 13.38±1.18 | 4.94±2.17 | 18.59±4.33 | 8.77±1.12 | 3.15±0.53 | 4.17±0.46 | 12.21±1.91 | 5.61±1.97 |  |
| 32 | 1046 | 2167-14-8 | 1-Ethyl-1H-pyrrole-2-carboxaldehyde | Ketones | RI, MS | <1 | n.d. | n.d. | n.d. | n.d. | n.d. | n.d. | 12.11±0.12 | 1.66±0.1 |  |
| 33 | 1047 | 13877-91-3 | β-Ocimene | Alkenes | RI, MS | <1 | n.d. | n.d. | n.d. | n.d. | n.d. | n.d. | n.d. | n.d. |  |
| 34 | 1048 | 68917-11-3 | Basilene (isomer mixture) | Alkenes | RI, MS | <1 | n.d. | n.d. | n.d. | n.d. | n.d. | n.d. | n.d. | n.d. |  |
| 35 | 1058 | 2548-87-0 | (E)-2-Octenal | Aldehydes | RI, MS | 1.32469 | 2.06±0.13 | 1.1±0.05 | n.d. | n.d. | n.d. | 1.66±0.59 | 1.91±0.5 | 7.83±3.99 |  |
| 36 | 1064 | 1072-83-9 | 1-(1H-Pyrrol-2-yl)ethanone | Ketones | RI, MS | <1 | n.d. | n.d. | n.d. | n.d. | n.d. | n.d. | n.d. | n.d. |  |
| 37 | 1064 | 98-86-2 | 2-acetyl pyrrole | Ketones | RI, MS | <1 | n.d. | n.d. | n.d. | 0.26±0.03 | n.d. | n.d. | n.d. | n.d. |  |
| 38 | 1069 | 1669-44-9 | 3,5-Octadien-2-one | Ketones | RI, MS | 1.28684 | 5.36±0.66 | n.d. | 10.87±1.27 | n.d. | 1.37±0.57 | 2.64±0.48 | 3.56±0.07 | 66.83±5.92 |  |
| 39 | 1072 | 1128-16-1 | cis-2,5,5-Trimethyl-2-vinyltetrahydrofuran | Others | RI, MS | <1 | n.d. | n.d. | n.d. | n.d. | 6.3±0.53 | 6.77±0.43 | 17.63±0.12 | n.d. |  |
| 40 | 1072 | 1365-19-1 | trans-Linalool oxide (furanoid) | Aldehydes | RI, MS | 1.2669 | 18.95±1.29 | 19.32±0.71 | 20.89±1.32 | n.d. | 12.4±1 | 13.74±0.33 | 38.08±3.74 | 0.31±0.06 |  |
| 41 | 1079 | 111-14-8 | Heptanoic acid | Aldehydes | RI, MS | 1.00376 | n.d. | n.d. | n.d. | n.d. | n.d. | n.d. | n.d. | 60.95±9.42 |  |
| 42 | 1101 | 78-70-6 | Linalool | Alcohols | RI, MS | <1 | 51.22±1.69 | 39.72±2.67 | 38.83±2.64 | 40.67±0.99 | 46.63±1.75 | 34.55±0.82 | 44.06±1.18 | 61.33±4.91 |  |
| 43 | 1104 | 78-70-6 | 3,7-Dimethyl-1,5,7-octatrien-3-ol | Alcohols | RI, MS | <1 | 13.03±0.55 | n.d. | 29.7±0.23 | n.d. | n.d. | 11.15±1.2 | 20.98±0.56 | 9.16±0.76 |  |
| 44 | 1105 | 124-19-6 | Nonanal | Aldehydes | RI, MS | <1 | n.d. | 5.45±0.26 | n.d. | 4.15±0.68 | 7.04±0.1 | n.d. | n.d. | n.d. |  |
| 45 | 1110 | 60-12-8 | Phenylethyl Alcohol | Alcohols | RI, MS | <1 | 18.47±0.83 | 18.42±0.93 | 17.57±8.39 | 19.42±5.62 | 6.83±1.83 | 7.72±0.61 | 28.91±4.29 | 9.16±2.02 |  |
| 46 | 1113 | 141-13-9 | (E)-4,8-Dimethylnona-1,3,7-triene | Alkenes | RI, MS | <1 | n.d. | n.d. | n.d. | n.d. | n.d. | n.d. | n.d. | n.d. |  |
| 47 | 1128 | 2049-96-9 | (E,Z)-2,6-Dimethyl-2,4,6-octatriene | Alkenes | RI, MS | <1 | n.d. | n.d. | n.d. | n.d. | n.d. | n.d. | n.d. | n.d. |  |
| 48 | 1128 | 2049-96-9 | 2,6-Dimethyl-2,4,6-octatriene | Alkenes | RI, MS | <1 | n.d. | n.d. | n.d. | n.d. | 1.17±0.01 | n.d. | n.d. | n.d. |  |
| 49 | 1133 | 140-29-4 | Benzyl Nitrile | Aldehydes | RI, MS | 1.20135 | n.d. | 0.54±0.05 | 0.83±0.15 | n.d. | n.d. | 4.13±0.53 | n.d. | n.d. |  |
| 50 | 1136 | 16433-88-8 | trans-3-Nonen-2-one | Ketones | RI, MS | <1 | 0.46±0.01 | 0.15±0.03 | 0.97±0.06 | n.d. | n.d. | n.d. | n.d. | 0.44±0.05 |  |
| 51 | 1148 | 104-53-0 | Lilac Aldehyde B | Aldehydes | RI, MS | <1 | n.d. | n.d. | n.d. | n.d. | n.d. | n.d. | n.d. | n.d. |  |
| 52 | 1150 | 557-48-2 | (E,Z)-2,6-Nonadienal | Aldehydes | RI, MS | 1.33696 | 0.69±0.02 | n.d. | 0.96±0.14 | n.d. | n.d. | n.d. | n.d. | 0.57±0.12 |  |
| 53 | 1152 | 59355-75-4 | (Z)-3-Nonen-1-ol | Alcohols | RI, MS | <1 | 0.81±0.03 | 1.59±0.23 | n.d. | 2.08±0.13 | 0.59±0.13 | 0.97±0.05 | 1±0.23 | n.d. |  |
| 54 | 1155 | 59355-75-4 | (E,Z)-3,6-Nonadien-1-ol | Alcohols | RI, MS | <1 | n.d. | 0.95±0.01 | n.d. | n.d. | n.d. | n.d. | n.d. | n.d. |  |
| 55 | 1158 | 18829-56-6 | (E)-2-Nonenal | Ketones | RI, MS | 1.27497 | 1.4±0.56 | 1.2±0.17 | 2.25±0.31 | 0.77±0.05 | 0.34±0.06 | 0.75±0.02 | 0.44±0.01 | 31.59±2.52 |  |
| 56 | 1161 | 140-11-4 | Benzyl Acetate | Esters | RI, MS | <1 | 2.12±0.93 | n.d. | n.d. | n.d. | n.d. | n.d. | 0.74±0.14 | n.d. |  |
| 57 | 1169 | 16409-43-1 | (3R,6S)-2,2,6-Trimethyl-6-vinyltetrahydro-2H-pyran-3-ol | Alcohols | RI, MS | <1 | 6.38±0.11 | 0.77±0.23 | 5.55±0.12 | 0.83±0.05 | 0.8±0.01 | 4.14±0.14 | 23.81±0.45 | 0.61±0.04 |  |
| 58 | 1172 | 143-08-8 | 1-Nonanol | Alcohols | RI, MS | <1 | 2.22±0.14 | 1.64±0.12 | 3.51±0.43 | 2.41±0.12 | 1.36±0.01 | 2.72±0.01 | n.d. | n.d. |  |
| 59 | 1178 | 1490-04-6 | dl-Menthol | Alcohols | RI, MS | <1 | n.d. | n.d. | n.d. | n.d. | n.d. | n.d. | n.d. | n.d. |  |
| 60 | 1179 | 2216-51-5 | Levomenthol | Alcohols | RI, MS | <1 | n.d. | n.d. | n.d. | n.d. | n.d. | n.d. | n.d. | n.d. |  |
| 61 | 1181 | 562-74-3 | Terpinen-4-ol | Others | RI, MS | 1.25981 | 0.38±0.06 | n.d. | 0.28±0.04 | n.d. | n.d. | n.d. | n.d. | n.d. |  |
| 62 | 1185 | 91-20-3 | Naphthalene | Alkenes | RI, MS | <1 | n.d. | n.d. | n.d. | n.d. | n.d. | n.d. | n.d. | n.d. |  |
| 63 | 1187 | 16409-43-1 | (E)-3-Hexenyl Butanoate | Alcohols | RI, MS | 1.03192 | n.d. | n.d. | n.d. | n.d. | n.d. | n.d. | n.d. | n.d. |  |
| 64 | 1187 | 67674-46-8 | cis-3-Hexenyl Isobutyrate | Esters | RI, MS | <1 | 0.43±0.22 | 0.67±0.01 | n.d. | 0.84±0.01 | 0.53±0.02 | n.d. | 1.28±0.01 | 26.26±0.78 |  |
| 65 | 1187 | 16409-43-1 | (Z)-3-Hexenyl Butanoate | Esters | RI, MS | <1 | n.d. | n.d. | n.d. | n.d. | n.d. | 4.16±0.43 | n.d. | n.d. |  |
| 66 | 1194 | 119-36-8 | Methyl Salicylate | Esters | RI, MS | 1.25782 | 16.8±0.88 | 24.25±6.53 | 23.71±1.44 | 25.11±11.34 | 19.72±4.84 | 32.45±2.75 | 46.61±6.25 | 21.6±5.05 |  |
| 67 | 1198 | 98-55-5 | α-Terpineol | Esters | RI, MS | 1.06755 | 5.19±0.43 | 2.62±0.13 | 2.31±0.51 | n.d. | 3.85±0.58 | 3.04±0.34 | 3.83±0.23 | 2.32±0.73 |  |
| 68 | 1198 | 98-55-5 | L-α-Terpineol | Alcohols | RI, MS | <1 | n.d. | n.d. | n.d. | 1.46±0.2 | n.d. | n.d. | n.d. | n.d. |  |
| 69 | 1201 | 432-25-7 | 2,6,6-Trimethyl-1-cyclohexadiene-1-carboxaldehyde | Alcohols | RI, MS | 1.18762 | n.d. | n.d. | n.d. | 1.31±0.02 | n.d. | 0.8±0.01 | n.d. | 1.26±0.08 |  |
| 70 | 1208 | 112-31-2 | Decanal | Esters | RI, MS | 1.2538 | 1.63±0.21 | 0.78±0.01 | 2.26±0.6 | 0.89±0.16 | 0.54±0.05 | n.d. | 1.33±0.8 | 1.4±0.34 |  |
| 71 | 1217 | 5910-87-2 | (E,E)-2,4-Nonadienal | Alcohols | RI, MS | 1.0351 | 0.18±0.03 | n.d. | 0.45±0.03 | n.d. | n.d. | n.d. | 0.15±0.05 | 0.12±0.01 |  |
| 72 | 1221 | 432-25-7 | 2,6,6-Trimethyl-1-cyclohexene-1-carboxaldehyde | Aldehydes | RI, MS | <1 | 4.36±0.03 | n.d. | n.d. | n.d. | n.d. | n.d. | n.d. | n.d. |  |
| 73 | 1227 | 106-25-2 | nerol | Aldehydes | RI, MS | 1.30751 | 4.79±0.5 | 3.28±0.58 | 2.67±0.35 | 3.29±0.34 | 5.01±0.8 | 5.93±1.3 | 7.48±0.59 | 40.94±5.43 |  |
| 74 | 1231 | 80-56-8 | 1,7,7-Trimethylbicyclo[2.2.1]hept-2-ene | Alkenes | RI, MS | <1 | 1.82±0.11 | n.d. | 4.7±0.12 | n.d. | n.d. | n.d. | 0.92±0.01 | n.d. |  |
| 75 | 1233 | 67674-46-8 | cis-3-Hexenyl α-Methylbutyrate | Aldehydes | RI, MS | 1.3142 | 0.64±0.02 | n.d. | n.d. | 0.87±0.22 | 0.92±0.01 | 3.05±0.61 | 0.6±0.11 | n.d. |  |
| 76 | 1238 | 67674-46-8 | (E,Z)-3-Hexenyl 2-Butenoate | Esters | RI, MS | <1 | n.d. | n.d. | n.d. | n.d. | n.d. | n.d. | n.d. | n.d. |  |
| 77 | 1238 | 67674-46-8 | cis-3-Hexenyl Isovalerate | Esters | RI, MS | <1 | n.d. | n.d. | n.d. | n.d. | n.d. | n.d. | n.d. | n.d. |  |
| 78 | 1240 | 106-26-3 | Neral | Aldehydes | RI, MS | 1.1463 | 3.58±0.58 | 4.9±0.68 | n.d. | 4.53±0.66 | 4.85±0.99 | 6.99±0.43 | 4.45±1.05 | n.d. |  |
| 79 | 1253 | 106-24-1 | Geraniol | Alcohols | RI, MS | 1.37991 | 653.31±17.47 | 770.61±7.68 | n.d. | 798.23±20.2 | 932.1±35.98 | 2056.94±33.92 | 1309.56±10.09 | 351.46±21.49 |  |
| 80 | 1257 | 432-25-7 | 1-Cyclohexene-1-acetaldehyde, 2,6,6-trimethyl- | Aldehydes | RI, MS | <1 | n.d. | n.d. | n.d. | n.d. | n.d. | n.d. | n.d. | n.d. |  |
| 81 | 1261 | 25101-02-4 | Pentanoic acid, 2-methyl-, anhydride | Acids | RI, MS | <1 | n.d. | n.d. | n.d. | n.d. | n.d. | n.d. | 0.86±0.07 | n.d. |  |
| 82 | 1264 | 3913-81-3 | (E)-2-Decenal | Alcohols | RI, MS | 1.30969 | 0.37±0.07 | n.d. | n.d. | n.d. | n.d. | 0.48±0.17 | n.d. | n.d. |  |
| 83 | 1270 | 5392-40-5 | Citral | Esters | RI, MS | 1.08292 | 7.46±0.96 | n.d. | 5.27±0.8 | n.d. | n.d. | n.d. | n.d. | n.d. |  |
| 84 | 1270 | 141-27-5 | (E)-citral trans-3,7-dimethylocta-2,6-dienal | Esters | RI, MS | 1.32215 | n.d. | 8.42±3.84 | n.d. | 11.89±2.95 | 8.14±1.18 | 15.63±1.24 | 12.76±0.82 | 0.45±0.03 |  |
| 85 | 1274 | 112-05-0 | Nonanoic Acid | Acids | RI, MS | <1 | n.d. | n.d. | n.d. | n.d. | n.d. | n.d. | n.d. | n.d. |  |
| 86 | 1294 | 120-72-9 | Indole | Alcohols | RI, MS | 1.22019 | n.d. | n.d. | n.d. | n.d. | n.d. | 4.32±0.58 | n.d. | n.d. |  |
| 87 | 1295 | 536-60-7 | p-Cymen-7-ol | Alcohols | RI, MS | <1 | n.d. | n.d. | n.d. | n.d. | n.d. | n.d. | n.d. | n.d. |  |
| 88 | 1295 | 89-83-8 | Phenol, 2-methyl-5-(1-methylethyl)- | Alcohols | RI, MS | <1 | 0.31±0.01 | n.d. | n.d. | n.d. | n.d. | n.d. | n.d. | n.d. |  |
| 89 | 1300 | 612-22-6 | Benzene, (2-nitroethyl)- | Alcohols | RI, MS | 1.19049 | n.d. | n.d. | n.d. | n.d. | n.d. | 0.28±0.06 | n.d. | n.d. |  |
| 90 | 1302 | 105-86-2 | (Z)-2,6-Octadien-1-ol, 3,7-dimethyl-, formate | Alcohols | RI, MS | <1 | n.d. | n.d. | n.d. | n.d. | n.d. | 1.44±0.08 | n.d. | n.d. |  |
| 91 | 1303 | 105-86-2 | Geranyl Formate | Alcohols | RI, MS | <1 | n.d. | 0.3±0.12 | n.d. | n.d. | 0.45±0.04 | n.d. | n.d. | n.d. |  |
| 92 | 1303 | 111-02-4 | 3,7,11-Trimethyl-2,6,10-dodecatrien-1-ol | Alcohols | RI, MS | <1 | 0.74±0.01 | n.d. | n.d. | n.d. | n.d. | n.d. | 0.44±0.03 | n.d. |  |
| 93 | 1311 | 112-44-7 | Undecanal | Aldehydes | RI, MS | <1 | n.d. | n.d. | n.d. | n.d. | n.d. | n.d. | n.d. | n.d. |  |
| 94 | 1318 | 67874-81-1 | 1-Oxaspiro[4.5]dec-6-ene, 2,6,10,10-tetramethyl- | Ketones | RI, MS | <1 | n.d. | n.d. | n.d. | 0.44±0.05 | n.d. | n.d. | n.d. | n.d. |  |
| 95 | 1320 | 25152-84-5 | (E,E)-2,4-Decadienal | Ketones | RI, MS | 1.2021 | n.d. | n.d. | n.d. | n.d. | n.d. | n.d. | n.d. | n.d. |  |
| 96 | 1321 | 514-81-2 | (Z)-1,5,9-Undecatriene, 2,6,10-trimethyl- | Alkenes | RI, MS | <1 | 0.7±0.01 | n.d. | 1.24±0.08 | n.d. | n.d. | n.d. | n.d. | n.d. |  |
| 97 | 1323 | 105-87-3 | trans-Geranic Acid Methyl Ester | Esters | RI, MS | <1 | 1.18±0.2 | n.d. | n.d. | 0.91±0.08 | 0.56±0.07 | 0.61±0.06 | n.d. | n.d. |  |
| 98 | 1347 | 6846-50-0 | 2,2,4-Trimethyl-1,3-pentanediol Diisobutyrate | Esters | RI, MS | <1 | 0.18±0.02 | 0.57±0.12 | 0.67±0.06 | 0.38±0.05 | 0.29±0.03 | 0.62±0.04 | 0.48±0.04 | 0.58±0.09 |  |
| 99 | 1349 | 17699-14-8 | α-Cubebene | Alkenes | RI, MS | <1 | n.d. | n.d. | n.d. | 0.98±0.71 | 0.36±0.11 | n.d. | n.d. | n.d. |  |
| 100 | 1352 | 544-63-8 | Naphthalene, 1,2-dihydro-1,1,6-trimethyl- | Ketones | RI, MS | 1.35362 | n.d. | n.d. | n.d. | n.d. | n.d. | n.d. | n.d. | n.d. |  |
| 101 | 1354 | 68916-26-7 | (E)-2,6-Octadienoic Acid, 3,7-dimethyl- | Acids | RI, MS | <1 | n.d. | n.d. | n.d. | n.d. | n.d. | n.d. | 2.59±0.23 | n.d. |  |
| 102 | 1358 | 104-61-0 | 2(3H)-Furanone, dihydro-5-pentyl- | Others | RI, MS | <1 | 0.2±0.03 | n.d. | 0.48±0.06 | n.d. | n.d. | n.d. | n.d. | n.d. |  |
| 103 | 1363 | 124-17-4 | Ethanol, 2-(2-butoxyethoxy)-, acetate | Alcohols | RI, MS | <1 | n.d. | n.d. | n.d. | n.d. | n.d. | n.d. | n.d. | n.d. |  |
| 104 | 1372 | 77-36-1 | Propanoic Acid, 2-methyl-, 3-hydroxy-2,2,4-trimethylpentyl Ester | Acids | RI, MS | <1 | n.d. | n.d. | n.d. | n.d. | n.d. | n.d. | n.d. | n.d. |  |
| 105 | 1373 | 5096-71-9 | 2-Octenal, 2-butyl- | Acids | RI, MS | 1.13237 | 0.63±0.07 | 1.32±0.2 | n.d. | n.d. | n.d. | 1.06±0.17 | n.d. | n.d. |  |
| 106 | 1375 | 143-15-7 | 2-Bromo dodecane | Alkanes | RI, MS | <1 | n.d. | n.d. | n.d. | n.d. | n.d. | n.d. | n.d. | n.d. |  |
| 107 | 1380 | 2363-88-4 | 2-Buten-1-one, 1-(2,6,6-trimethyl-1,3-cyclohexadien-1-yl)-, (E)- | Others | RI, MS | 1.279 | 2.48±0.6 | n.d. | 2.18±0.05 | n.d. | n.d. | n.d. | n.d. | n.d. |  |
| 108 | 1381 | 105-87-3 | Geranyl acetate | Esters | RI, MS | <1 | n.d. | 0.45±0.23 | n.d. | n.d. | 0.56±0.07 | 1.51±0.07 | n.d. | n.d. |  |
| 109 | 1384 | 33467-74-2 | (Z)-3-Hexen-1-yl hexanoate | Esters | RI, MS | <1 | n.d. | n.d. | n.d. | n.d. | n.d. | 13.28±0.87 | n.d. | n.d. |  |
| 110 | 1384 | 33467-74-2 | (Z)-4-Hexen-1-yl hexanoate | Esters | RI, MS | <1 | n.d. | 1.2±0.15 | 1.18 | 1.06±0.12 | 0.69±0.12 | n.d. | 2.31±0.17 | 4.49±0.06 |  |
| 111 | 1389 | 16491-36-4 | cis-3-Hexenyl cis-3-hexenoate | Esters | RI, MS | <1 | n.d. | n.d. | n.d. | n.d. | n.d. | 0.89±0.06 | n.d. | n.d. |  |
| 112 | 1391 | 6378-65-0 | Hexanoic acid, hexyl ester | Alcohols | RI, MS | 1.02545 | 0.4±0.04 | n.d. | n.d. | n.d. | n.d. | 1.94±0.57 | 0.71±0.03 | n.d. |  |
| 113 | 1395 | 488-10-8 | (Z)-Jasmone | Ketones | RI, MS | <1 | 2.56±0.26 | 4.66±1.59 | 1.4±0.44 | n.d. | 3.09±0.62 | 15.87±2.34 | 6.73±0.41 | 32.21±3.1 |  |
| 114 | 1418 | 112-54-9 | Dodecanal | Aldehydes | RI, MS | <1 | n.d. | n.d. | n.d. | n.d. | n.d. | n.d. | n.d. | n.d. |  |
| 115 | 1425 | 87-44-5 | Caryophyllene | Alkenes | RI, MS | <1 | n.d. | n.d. | n.d. | n.d. | n.d. | n.d. | n.d. | n.d. |  |
| 116 | 1428 | 127-41-3 | α-Ionone | Others | RI, MS | 1.30697 | 1.56±0.11 | 0.56±0.08 | 1.05±0.83 | 0.71±0.07 | 0.13±0.03 | 0.73±0.16 | 0.38±0.09 | n.d. |  |
| 117 | 1447 | 103-45-7 | Propanoic acid, 2-methyl-, 2-phenylethyl ester | Aldehydes | RI, MS | 1.29845 | n.d. | n.d. | n.d. | n.d. | n.d. | n.d. | n.d. | n.d. |  |
| 118 | 1456 | 16984-13-7 | 5,9-Undecadien-2-one, 6,10-dimethyl- | Aldehydes | RI, MS | 1.24872 | 2.22±0.41 | 0.8±0.02 | 2.41±0.51 | 1.67±0.56 | 0.46±0.11 | 1.34±0.05 | 0.7±0.03 | 2.21±0.11 |  |
| 119 | 1463 | 18794-84-8 | (E)-β-Farnesene | Aldehydes | RI, MS | 1.13971 | n.d. | n.d. | n.d. | n.d. | n.d. | n.d. | n.d. | n.d. |  |
| 120 | 1483 | 2363-88-4 | 4-(2,6,6-Trimethylcyclohexa-1,3-dienyl)but-3-en-2-one | Ketones | RI, MS | <1 | n.d. | n.d. | n.d. | n.d. | n.d. | n.d. | n.d. | n.d. |  |
| 121 | 1485 | 14901-07-6 | β-Lonone | Ketones | RI, MS | <1 | 8.02±0.23 | n.d. | 6.88±0.47 | n.d. | 2.15±0.11 | 3.81±0.14 | 1.98±0.04 | 2.07±0.2 |  |
| 122 | 1490 | 122-57-6 | 5-Methyl-2-phenyl-2-hexenal | Ketones | RI, MS | <1 | 0.47±0.12 | n.d. | n.d. | n.d. | n.d. | n.d. | n.d. | n.d. |  |
| 123 | 1492 | 104-61-0 | 2H-Pyran-2-one, tetrahydro-6-(2-pentenyl)-, (Z)- | Others | RI, MS | 1.29651 | n.d. | n.d. | n.d. | n.d. | n.d. | 1.92±0.54 | n.d. | n.d. |  |
| 124 | 1495 | 104-61-0 | 2H-Pyran-2-one, tetrahydro-6-pentyl- | Others | RI, MS | <1 | n.d. | n.d. | n.d. | n.d. | n.d. | n.d. | n.d. | n.d. |  |
| 125 | 1501 | 6750-60-3 | (3S,3aR,3bR,4S,7R,7aR)-4-Isopropyl-3,7-dimethyloctahydro-1H-cyclopenta[1,3]cyclopropa[1,2]benzen-3-ol | Alcohols | RI, MS | <1 | n.d. | n.d. | n.d. | n.d. | n.d. | n.d. | n.d. | n.d. |  |
| 126 | 1510 | 128-37-0 | Butylated Hydroxytoluene | Others | RI, MS | <1 | 0.31±0.03 | n.d. | n.d. | n.d. | n.d. | 0.16±0.03 | 0.32±0.08 | n.d. |  |
| 127 | 1515 | 96-76-4 | 2,4-Di-tert-butylphenol | Others | RI, MS | <1 | 0.67±0.1 | 4.99±4.3 | 11.8±1.18 | 4.81±1.03 | n.d. | n.d. | 0.76±0.2 | n.d. |  |
| 128 | 1526 | 489-39-4 | Isoledene | Alkenes | RI, MS | <1 | 1.12±0.23 | 0.56±0.12 | n.d. | n.d. | 0.51±0.03 | n.d. | n.d. | n.d. |  |
| 129 | 1527 | 489-39-4 | 1-Isopropyl-4,7-dimethyl-1,2,3,5,6,8a-hexahydronaphthalene | Others | RI, MS | <1 | n.d. | n.d. | n.d. | n.d. | n.d. | n.d. | n.d. | n.d. |  |
| 130 | 1528 | 6813-99-0 | 2(4H)-Benzofuranone, 5,6,7,7a-tetrahydro-4,4,7a-trimethyl-, (R)- | Ketones | RI, MS | 1.10326 | n.d. | n.d. | n.d. | n.d. | n.d. | n.d. | n.d. | n.d. |  |
| 131 | 1568 | 106-32-1 | Butanoic acid, 3,7-dimethyl-2,6-octadienyl ester, (E)- | Esters | RI, MS | <1 | n.d. | n.d. | n.d. | n.d. | n.d. | 0.39±0.07 | n.d. | n.d. |  |
| 132 | 1572 | 7212-44-4 | nerolidol | Alcohols | RI, MS | 1.30969 | 2.46±0.2 | 4.66±0.12 | 5.92±1.23 | n.d. | 1.47±0.07 | 13.46±0.99 | 2.39±0.19 | 0.31±0.05 |  |
| 133 | 1580 | 1139-30-6 | 3-Hexen-1-ol, benzoate, (Z)- | Ketones | RI, MS | 1.01396 | 0.38±0.06 | n.d. | 0.55±0.06 | n.d. | n.d. | n.d. | n.d. | 0.35±0.06 |  |
| 134 | 1580 | 3063-30-5 | 3-Hexen-1-ol benzoate | Esters | RI, MS | <1 | n.d. | n.d. | n.d. | n.d. | n.d. | n.d. | n.d. | n.d. |  |
| 135 | 1587 | 629-94-7 | N-HENEICOSANE | Alkanes | RI, MS | <1 | n.d. | n.d. | n.d. | n.d. | n.d. | n.d. | n.d. | n.d. |  |
| 136 | 1588 | 1139-30-6 | Caryophyllene oxide | Others | RI, MS | <1 | n.d. | n.d. | n.d. | n.d. | n.d. | n.d. | n.d. | n.d. |  |
| 137 | 1588 | 84-66-2 | Diethyl Phthalate | Others | RI, MS | <1 | n.d. | n.d. | n.d. | n.d. | 1.02±0.02 | n.d. | n.d. | n.d. |  |
| 138 | 1614 | 77-53-2 | Cedrol | Alcohols | RI, MS | <1 | 0.63±0.13 | 0.5±0.14 | 1.15±0.38 | 2.61±0.55 | n.d. | 0.36±0.02 | n.d. | n.d. |  |
| 139 | 1655 | 71735-74-5 | Cyclopentaneacetic acid, 3-oxo-2-(2-pentenyl)-, methyl ester, [1.alpha.,2.alpha.(Z)]- | Esters | RI, MS | <1 | n.d. | n.d. | n.d. | n.d. | n.d. | 0.51±0.08 | n.d. | n.d. |  |
| 140 | 1661 | 1119-40-0 | Cyclopentaneacetic acid, 3-oxo-2-pentyl-, methyl ester | Esters | RI, MS | <1 | n.d. | n.d. | n.d. | n.d. | n.d. | n.d. | n.d. | n.d. |  |
| 141 | 1666 | 481-34-5 | α-Cadinol | Alcohols | RI, MS | <1 | n.d. | n.d. | 0.71±0.22 | n.d. | n.d. | n.d. | n.d. | n.d. |  |
| 142 | 1769 | 112-06-1 | neryl hexanoate | Esters | RI, MS | <1 | n.d. | n.d. | n.d. | n.d. | n.d. | 0.15±0.07 | n.d. | n.d. |  |
| 143 | 1798 | 6458-57-7 | 2-Hexyl-1-decano | Alkanes | RI, MS | <1 | n.d. | n.d. | n.d. | n.d. | n.d. | n.d. | 0.27±0.01 | n.d. |  |
| 144 | 1843 | 1937-52-0 | 2-Pentadecanone, 6,10,14-trimethyl- | Ketones | RI, MS | <1 | n.d. | n.d. | 0.51±0.23 | n.d. | n.d. | n.d. | n.d. | n.d. |  |
| 145 | 1945 | 112-39-0 | Hexadecanoic acid, methyl ester | Acids | RI, MS | <1 | 0.43±0.03 | 0.29±0.08 | 0.41±0.03 | 0.37±0.16 | 0.6±0.5 | 0.27±0.03 | 0.3±0.01 | 0.18±0.06 |  |
| 146 | 1960 | 57-10-3 | n-Hexadecanoic acid | Acids | RI, MS | <1 | 0.22±0.07 | 0.11±0.04 | 0.18±0.13 | n.d. | n.d. | 0.22±0.07 | 2.89±1.26 | 0.45±0.31 |  |
| 147 | 1985 | 84-74-2 | Dibutyl phthalate | Others | RI, MS | <1 | n.d. | n.d. | n.d. | n.d. | n.d. | n.d. | n.d. | n.d. |  |

| **No.** | **RI** | **CAS** | **Compounds** | **Types** | **Identification basis** | **VIP** | **Content (ug/L)** | | | | | | | |
| --- | --- | --- | --- | --- | --- | --- | --- | --- | --- | --- | --- | --- | --- | --- |
|  |  |  |  |  |  |  | **JG1** | **JG2** | **JG3** | **JG4** | **MZ1** | **MZ2** | **MZ3** | **MZ4** |
| 1 | 698 | 64-19-7 | Acetic acid | Acids | RI, MS | <1 | n.d. | n.d. | n.d. | n.d. | n.d. | n.d. | 0.15±0.07 | n.d. |
| 2 | 744 | 110-05-4 | Di-tert-butyl peroxide | Others | RI, MS | <1 | 0.9±0.04 | n.d. | n.d. | n.d. | n.d. | n.d. | n.d. | n.d. |
| 3 | 801 | 66-25-1 | Hexanal | Aldehydes | RI, MS | 1.3359 | 6.64±1.26 | 10.12±1.55 | 24.22±6.43 | 1.04±0.17 | n.d. | 2.65±0.32 | 3.05±0.65 | 3.76±1.93 |
| 4 | 828 | 98-01-1 | Furfural | Aldehydes | RI, MS | 1.18407 | 2.09±1.05 | 1.57±1.16 | 4.94±1.79 | 2.49±1.79 | n.d. | n.d. | n.d. | n.d. |
| 5 | 845 | 505-57-7 | 2-Hexenal | Aldehydes | RI, MS | <1 | 5.93±2.75 | 5.23±0.23 | n.d. | 5.99±6.84 | 2.83±0.06 | n.d. | n.d. | n.d. |
| 6 | 850 | 6728-26-3 | (E)-2-Hexenal | Aldehydes | RI, MS | <1 | n.d. | n.d. | n.d. | n.d. | n.d. | n.d. | 17±1.86 | n.d. |
| 7 | 852 | 33467-74-2 | (Z)-3-Hexen-1-ol formate | Alcohols | RI, MS | <1 | n.d. | n.d. | n.d. | n.d. | n.d. | n.d. | n.d. | n.d. |
| 8 | 853 | 928-96-1 | (E)-3-Hexen-1-ol | Alcohols | RI, MS | <1 | n.d. | n.d. | n.d. | n.d. | 11.88±0.12 | n.d. | n.d. | n.d. |
| 9 | 863 | 928-95-0 | (E)-2-Hexen-1-ol | Alcohols | RI, MS | <1 | n.d. | n.d. | 1.62±0.39 | n.d. | n.d. | 0.31±0.06 | n.d. | n.d. |
| 10 | 867 | 111-27-3 | 1-Hexanol | Ketones | RI, MS | 1.12692 | n.d. | n.d. | 2.58±0.85 | n.d. | 1±0.11 | n.d. | n.d. | n.d. |
| 11 | 889 | 110-43-0 | 2-Heptanone | Ketones | RI, MS | <1 | n.d. | n.d. | n.d. | n.d. | n.d. | n.d. | n.d. | n.d. |
| 12 | 901 | 111-71-7 | Heptanal | Aldehydes | RI, MS | <1 | n.d. | n.d. | n.d. | n.d. | 1.61±0.62 | 0.46±0 | n.d. | n.d. |
| 13 | 909 | 142-83-6 | (E,E)-2,4-Hexadienal | Aldehydes | RI, MS | <1 | n.d. | 1.97±1.66 | n.d. | 2.26±1.42 | n.d. | n.d. | n.d. | n.d. |
| 14 | 909 | 3208-16-0 | 2-Ethylfuran | Others | RI, MS | <1 | n.d. | n.d. | 2.08±1.16 | n.d. | n.d. | n.d. | n.d. | n.d. |
| 15 | 918 | 100-51-6 | Nerolidol | Alkenes | RI, MS | 1.07399 | n.d. | n.d. | n.d. | n.d. | 0.8±0.06 | n.d. | 0.2±0.03 | n.d. |
| 16 | 927 | 110-13-4 | 2,5-Hexanedione | Ketones | RI, MS | <1 | n.d. | n.d. | n.d. | n.d. | 0.08±0.01 | n.d. | n.d. | n.d. |
| 17 | 954 | 18829-55-5 | (E)-2-Heptenal | Others | RI, MS | 1.21947 | 1.63±0.17 | 1.57±0.58 | 1.62±0.14 | n.d. | n.d. | 0.5±0.06 | 0.37±0.08 | n.d. |
| 18 | 958 | 100-52-7 | Benzaldehyde | Esters | RI, MS | 1.10782 | 37.23±4.27 | 26.32±3.9 | 33.59±1.18 | 23.65±3.75 | 18.43±3.06 | 15.58±3.22 | 10.84±0.97 | 21.53±3.08 |
| 19 | 970 | 111-70-6 | 1-Heptanol | Alcohols | RI, MS | <1 | n.d. | 1.56±0.24 | 1.68±0.21 | n.d. | n.d. | n.d. | n.d. | n.d. |
| 20 | 976 | 4312-99-6 | 1-Octen-3-one | Ketones | RI, MS | <1 | n.d. | n.d. | n.d. | n.d. | n.d. | n.d. | n.d. | n.d. |
| 21 | 979 | 3391-86-4 | 1-Octen-3-ol | Alkenes | RI, MS | 1.25923 | 2.23±1.32 | 2.62±1.22 | 2.11±0.59 | n.d. | 0.79±0.2 | 2.13±0.19 | 1.34±0.07 | 1.35±0.04 |
| 22 | 983 | 110-93-0 | 6-Methyl-5-hepten-2-one | Aldehydes | RI, MS | 1.14005 | 6.44±1.68 | 2.38±1.27 | 4.3±0.62 | n.d. | n.d. | 1.12±0.09 | 1.76±0.03 | 2.11±1.29 |
| 23 | 989 | 123-35-3 | β-Myrcene | Ketones | RI, MS | 1.11163 | n.d. | n.d. | n.d. | n.d. | 14.75±3.36 | n.d. | 6.09±2.05 | 14.1±2.31 |
| 24 | 995 | 4313-03-5 | (E,E)-2,4-Heptadienal | Aldehydes | RI, MS | 1.12057 | 33.12±4.32 | 17.64±0.89 | 37.12±7.75 | 15.79±3.88 | 0.27±0.07 | 1.06±1.32 | 2.88±0.28 | 2.46±0.39 |
| 25 | 1002 | 124-13-0 | Octanal | Aldehydes | RI, MS | <1 | 0.96±0.36 | 1.58±0.47 | n.d. | 1.33±0.64 | n.d. | 0.24±0.12 | n.d. | 0.3±0.14 |
| 26 | 1024 | 99-87-6 | p-Cymene | Alkenes | RI, MS | <1 | n.d. | n.d. | n.d. | n.d. | 1.01±0.08 | n.d. | n.d. | n.d. |
| 27 | 1029 | 5989-27-5 | D-Limonene | Alkenes | RI, MS | <1 | n.d. | n.d. | n.d. | n.d. | n.d. | n.d. | n.d. | n.d. |
| 28 | 1032 | 100-51-6 | Benzyl alcohol | Aldehydes | RI, MS | 1.34865 | 3.35±2.16 | 5.98±1.09 | 10.3±6.38 | 5.92±1.52 | 18.76±2.12 | 4.34±0.71 | 12.21±1.75 | 33.85±2.26 |
| 29 | 1037 | 3779-61-1 | trans-β-Ocimene | Alkenes | RI, MS | <1 | n.d. | n.d. | n.d. | n.d. | n.d. | n.d. | n.d. | n.d. |
| 30 | 1037 | 1669-44-9 | 3-Octen-2-one | Others | RI, MS | 1.10109 | 2.05±1.33 | 2.57±0.57 | 3.61±0.3 | n.d. | n.d. | n.d. | n.d. | n.d. |
| 31 | 1042 | 122-78-1 | benzeneacetaldehyde | Aldehydes | RI, MS | 1.07435 | 20.51±2.73 | 15.61±0.72 | 24.05±5.52 | 13.6±8.32 | 4.23±0.28 | 12.9±1.28 | 6.05±0.69 | 10.79±5.19 |
| 32 | 1046 | 2167-14-8 | 1-Ethyl-1H-pyrrole-2-carboxaldehyde | Ketones | RI, MS | <1 | n.d. | n.d. | n.d. | n.d. | 17.05±1.45 | 3.32±0.67 | n.d. | n.d. |
| 33 | 1047 | 13877-91-3 | β-Ocimene | Alkenes | RI, MS | <1 | n.d. | n.d. | n.d. | n.d. | n.d. | n.d. | n.d. | n.d. |
| 34 | 1048 | 68917-11-3 | Basilene (isomer mixture) | Alkenes | RI, MS | <1 | n.d. | n.d. | n.d. | n.d. | n.d. | n.d. | n.d. | n.d. |
| 35 | 1058 | 2548-87-0 | (E)-2-Octenal | Aldehydes | RI, MS | 1.32469 | 6.09±0.79 | 6.35±0.59 | 7.39±1.52 | 4±1.83 | n.d. | 1.48±0.15 | 1.86±0.17 | 1.76±0.66 |
| 36 | 1064 | 1072-83-9 | 1-(1H-Pyrrol-2-yl)ethanone | Ketones | RI, MS | <1 | n.d. | n.d. | n.d. | n.d. | n.d. | n.d. | n.d. | n.d. |
| 37 | 1064 | 98-86-2 | 2-acetyl pyrrole | Ketones | RI, MS | <1 | 0.46±0.27 | n.d. | n.d. | 0.4±0.42 | n.d. | n.d. | n.d. | n.d. |
| 38 | 1069 | 1669-44-9 | 3,5-Octadien-2-one | Ketones | RI, MS | 1.28684 | 11.36±1.09 | 11.27±1.51 | 15.23±2.85 | 9±0.11 | 1.55±0.16 | 6.07±1.82 | 6.54±1.17 | 7.67±0.59 |
| 39 | 1072 | 1128-16-1 | cis-2,5,5-Trimethyl-2-vinyltetrahydrofuran | Others | RI, MS | <1 | n.d. | n.d. | n.d. | n.d. | 19.54±0.78 | 12.82±0.21 | n.d. | n.d. |
| 40 | 1072 | 1365-19-1 | trans-Linalool oxide (furanoid) | Aldehydes | RI, MS | 1.2669 | 8.48±0.35 | 17.67±1.98 | 33.49±5.93 | 18.67±0.09 | 38.16±4.38 | 16.92±4.76 | 23.13±2.23 | 52.57±5.83 |
| 41 | 1079 | 111-14-8 | Heptanoic acid | Aldehydes | RI, MS | 1.00376 | n.d. | n.d. | 1.13±0.01 | n.d. | n.d. | n.d. | n.d. | n.d. |
| 42 | 1101 | 78-70-6 | Linalool | Alcohols | RI, MS | <1 | 28.85±8.13 | 41.48±0.28 | 35.57±4.03 | 25.95±1 | 35.1±1.74 | 44.42±4.14 | 62.45±0.1 | 82.09±13.43 |
| 43 | 1104 | 78-70-6 | 3,7-Dimethyl-1,5,7-octatrien-3-ol | Alcohols | RI, MS | <1 | 17.79±0.83 | 22.82 | 25.67±0.12 | 14.4 | n.d. | 11.97±0.2 | n.d. | 11.35±0.88 |
| 44 | 1105 | 124-19-6 | Nonanal | Aldehydes | RI, MS | <1 | n.d. | n.d. | n.d. | n.d. | n.d. | n.d. | 7.59±0.09 | n.d. |
| 45 | 1110 | 60-12-8 | Phenylethyl Alcohol | Alcohols | RI, MS | <1 | 10.59±4.3 | 11.27 | 36.23±2.22 | 12.77±3.14 | 11.68±2.75 | 19.3±7.58 | 14.82±1.16 | 30.67±20.21 |
| 46 | 1113 | 141-13-9 | (E)-4,8-Dimethylnona-1,3,7-triene | Alkenes | RI, MS | <1 | 0.59±0.03 | 0.58±0.09 | n.d. | n.d. | n.d. | n.d. | n.d. | n.d. |
| 47 | 1128 | 2049-96-9 | (E,Z)-2,6-Dimethyl-2,4,6-octatriene | Alkenes | RI, MS | <1 | n.d. | n.d. | n.d. | n.d. | 1.72±0.13 | n.d. | n.d. | n.d. |
| 48 | 1128 | 2049-96-9 | 2,6-Dimethyl-2,4,6-octatriene | Alkenes | RI, MS | <1 | n.d. | n.d. | n.d. | n.d. | n.d. | n.d. | n.d. | n.d. |
| 49 | 1133 | 140-29-4 | Benzyl Nitrile | Aldehydes | RI, MS | 1.20135 | 15.06±2.11 | 10.18±1.97 | 18.41±1.34 | 11.32±2.65 | 2.04±0.22 | n.d. | n.d. | n.d. |
| 50 | 1136 | 16433-88-8 | trans-3-Nonen-2-one | Ketones | RI, MS | <1 | n.d. | n.d. | n.d. | n.d. | n.d. | 0.26±0.01 | 0.36±0.23 | 0.36±0.01 |
| 51 | 1148 | 104-53-0 | Lilac Aldehyde B | Aldehydes | RI, MS | <1 | n.d. | n.d. | n.d. | n.d. | n.d. | n.d. | n.d. | n.d. |
| 52 | 1150 | 557-48-2 | (E,Z)-2,6-Nonadienal | Aldehydes | RI, MS | 1.33696 | 2.16±0.33 | 1.09±0.04 | 1.58±0.04 | 1.52±0.57 | n.d. | 0.48±0.02 | 0.64±0.09 | 1.08±0.18 |
| 53 | 1152 | 59355-75-4 | (Z)-3-Nonen-1-ol | Alcohols | RI, MS | <1 | n.d. | n.d. | n.d. | n.d. | n.d. | 0.58±0.01 | 0.82±0.07 | n.d. |
| 54 | 1155 | 59355-75-4 | (E,Z)-3,6-Nonadien-1-ol | Alcohols | RI, MS | <1 | n.d. | n.d. | n.d. | n.d. | n.d. | n.d. | n.d. | n.d. |
| 55 | 1158 | 18829-56-6 | (E)-2-Nonenal | Ketones | RI, MS | 1.27497 | 2.36±0.52 | 2.31±0.17 | 3.17±0.02 | 1.65±0.13 | 0.53±0.17 | 0.81±0.03 | 0.85±0.07 | 1.03±0.3 |
| 56 | 1161 | 140-11-4 | Benzyl Acetate | Esters | RI, MS | <1 | n.d. | n.d. | n.d. | n.d. | n.d. | 1.19±0.29 | n.d. | n.d. |
| 57 | 1169 | 16409-43-1 | (3R,6S)-2,2,6-Trimethyl-6-vinyltetrahydro-2H-pyran-3-ol | Alcohols | RI, MS | <1 | n.d. | 2.57±0.01 | 4.79±0.13 | 1.73±0.01 | 9.86±0.01 | n.d. | 6.64±0.06 | 21.16±0.41 |
| 58 | 1172 | 143-08-8 | 1-Nonanol | Alcohols | RI, MS | <1 | 1.68±0.01 | 2.5±0.06 | 4.36±0.43 | 1.74±0.02 | 3.41±0.01 | n.d. | 2.59±0.02 | n.d. |
| 59 | 1178 | 1490-04-6 | dl-Menthol | Alcohols | RI, MS | <1 | n.d. | n.d. | n.d. | n.d. | n.d. | n.d. | n.d. | n.d. |
| 60 | 1179 | 2216-51-5 | Levomenthol | Alcohols | RI, MS | <1 | n.d. | n.d. | n.d. | n.d. | n.d. | 0.47±0.12 | n.d. | n.d. |
| 61 | 1181 | 562-74-3 | Terpinen-4-ol | Others | RI, MS | 1.25981 | n.d. | n.d. | n.d. | n.d. | n.d. | 0.29±0.05 | n.d. | n.d. |
| 62 | 1185 | 91-20-3 | Naphthalene | Alkenes | RI, MS | <1 | n.d. | n.d. | n.d. | n.d. | n.d. | n.d. | n.d. | n.d. |
| 63 | 1187 | 16409-43-1 | (E)-3-Hexenyl Butanoate | Alcohols | RI, MS | 1.03192 | 4.46±1.1 | 6.38±1.08 | 6.12±0.11 | 3.59±0.6 | n.d. | n.d. | n.d. | n.d. |
| 64 | 1187 | 67674-46-8 | cis-3-Hexenyl Isobutyrate | Esters | RI, MS | <1 | n.d. | n.d. | n.d. | n.d. | n.d. | 0.41±0.01 | n.d. | 1.35±0.23 |
| 65 | 1187 | 16409-43-1 | (Z)-3-Hexenyl Butanoate | Esters | RI, MS | <1 | n.d. | n.d. | n.d. | n.d. | n.d. | n.d. | 1.73±0.01 | n.d. |
| 66 | 1194 | 119-36-8 | Methyl Salicylate | Esters | RI, MS | 1.25782 | 20.35±0.18 | 29.56±1.05 | 35.35±2.17 | 20.51±5.32 | 36.41±4.22 | n.d. | 43.98±3.69 | 8.23±1.36 |
| 67 | 1198 | 98-55-5 | α-Terpineol | Esters | RI, MS | 1.06755 | n.d. | n.d. | n.d. | n.d. | 4.51±1.16 | 10.86±2.02 | 4.86±1.17 | 7.52±1.09 |
| 68 | 1198 | 98-55-5 | L-α-Terpineol | Alcohols | RI, MS | <1 | n.d. | n.d. | n.d. | n.d. | n.d. | n.d. | n.d. | n.d. |
| 69 | 1201 | 432-25-7 | 2,6,6-Trimethyl-1-cyclohexadiene-1-carboxaldehyde | Alcohols | RI, MS | 1.18762 | 1.76±0.57 | 1.97±0.07 | 2.02±0.58 | 1.72±0.57 | n.d. | n.d. | n.d. | n.d. |
| 70 | 1208 | 112-31-2 | Decanal | Esters | RI, MS | 1.2538 | 2.05±0.28 | 1.97±0.16 | 2.19±0.32 | 0.98±0.12 | n.d. | 1.16±0.2 | 0.95±0.05 | 0.17±0.01 |
| 71 | 1217 | 5910-87-2 | (E,E)-2,4-Nonadienal | Alcohols | RI, MS | 1.0351 | 0.82±0.01 | 0.97±0.02 | 1.17±0.08 | 0.7±0.14 | n.d. | n.d. | n.d. | 0.15±0.03 |
| 72 | 1221 | 432-25-7 | 2,6,6-Trimethyl-1-cyclohexene-1-carboxaldehyde | Aldehydes | RI, MS | <1 | n.d. | n.d. | n.d. | n.d. | n.d. | n.d. | n.d. | n.d. |
| 73 | 1227 | 106-25-2 | nerol | Aldehydes | RI, MS | 1.30751 | 0.8±0.12 | 2.14±0.14 | 3.96±0.72 | n.d. | 10.87±2.03 | 3.97±1.36 | 7.6±1.89 | 10.37±0.25 |
| 74 | 1231 | 80-56-8 | 1,7,7-Trimethylbicyclo[2.2.1]hept-2-ene | Alkenes | RI, MS | <1 | n.d. | n.d. | n.d. | n.d. | n.d. | 1.16±0.23 | n.d. | n.d. |
| 75 | 1233 | 67674-46-8 | cis-3-Hexenyl α-Methylbutyrate | Aldehydes | RI, MS | 1.3142 | 3.45±0.93 | 4.73±0.59 | 5.58±0.73 | 2.1±0.58 | n.d. | 0.45±0.05 | 2.17±0.35 | 1.16±0.35 |
| 76 | 1238 | 67674-46-8 | (E,Z)-3-Hexenyl 2-Butenoate | Esters | RI, MS | <1 | n.d. | n.d. | n.d. | n.d. | n.d. | n.d. | n.d. | n.d. |
| 77 | 1238 | 67674-46-8 | cis-3-Hexenyl Isovalerate | Esters | RI, MS | <1 | n.d. | n.d. | n.d. | n.d. | n.d. | n.d. | n.d. | n.d. |
| 78 | 1240 | 106-26-3 | Neral | Aldehydes | RI, MS | 1.1463 | n.d. | 5.06±0.13 | 6.15±0.73 | n.d. | 6.77±1.03 | 3.08±0.44 | 10.03±0.34 | 10.47±0.31 |
| 79 | 1253 | 106-24-1 | Geraniol | Alcohols | RI, MS | 1.37991 | 248.26±21.44 | 537.4±23.5 | 669.49±3.28 | 355.42±6.39 | 2381.7±14.75 | 538.02±15.01 | 1409.16±46.05 | 1870.42±51.56 |
| 80 | 1257 | 432-25-7 | 1-Cyclohexene-1-acetaldehyde, 2,6,6-trimethyl- | Aldehydes | RI, MS | <1 | 0.93±0.01 | n.d. | n.d. | n.d. | n.d. | n.d. | n.d. | n.d. |
| 81 | 1261 | 25101-02-4 | Pentanoic acid, 2-methyl-, anhydride | Acids | RI, MS | <1 | n.d. | n.d. | 0.66±0.2 | n.d. | n.d. | n.d. | n.d. | n.d. |
| 82 | 1264 | 3913-81-3 | (E)-2-Decenal | Alcohols | RI, MS | 1.30969 | 1.59±0.12 | 1.53±0.08 | 2.71±1.33 | 1.3±0.12 | n.d. | 0.27±0.01 | n.d. | n.d. |
| 83 | 1270 | 5392-40-5 | Citral | Esters | RI, MS | 1.08292 | 4.45±0.59 | n.d. | n.d. | n.d. | n.d. | 6.09±0.11 | n.d. | n.d. |
| 84 | 1270 | 141-27-5 | (E)-citral trans-3,7-dimethylocta-2,6-dienal | Esters | RI, MS | 1.32215 | n.d. | n.d. | 10.18±2.24 | 5.4±0.87 | 18.32±1.2 | n.d. | 16.54±1.59 | 17.66±2.9 |
| 85 | 1274 | 112-05-0 | Nonanoic Acid | Acids | RI, MS | <1 | n.d. | n.d. | n.d. | n.d. | 2.56±0.23 | 2.33±0.07 | n.d. | n.d. |
| 86 | 1294 | 120-72-9 | Indole | Alcohols | RI, MS | 1.22019 | 5.58±0.93 | 6.16±1.23 | 5.5±1.17 | 4±0.75 | n.d. | n.d. | n.d. | n.d. |
| 87 | 1295 | 536-60-7 | p-Cymen-7-ol | Alcohols | RI, MS | <1 | n.d. | n.d. | n.d. | n.d. | n.d. | 0.28±0.21 | n.d. | n.d. |
| 88 | 1295 | 89-83-8 | Phenol, 2-methyl-5-(1-methylethyl)- | Alcohols | RI, MS | <1 | n.d. | n.d. | n.d. | n.d. | n.d. | n.d. | n.d. | n.d. |
| 89 | 1300 | 612-22-6 | Benzene, (2-nitroethyl)- | Alcohols | RI, MS | 1.19049 | n.d. | 4.08±0.09 | 7.35±0.34 | 4.39±1.56 | n.d. | n.d. | n.d. | n.d. |
| 90 | 1302 | 105-86-2 | (Z)-2,6-Octadien-1-ol, 3,7-dimethyl-, formate | Alcohols | RI, MS | <1 | n.d. | n.d. | n.d. | n.d. | n.d. | n.d. | 1.06±0.02 | n.d. |
| 91 | 1303 | 105-86-2 | Geranyl Formate | Alcohols | RI, MS | <1 | n.d. | n.d. | n.d. | n.d. | n.d. | n.d. | n.d. | 1.12±0.15 |
| 92 | 1303 | 111-02-4 | 3,7,11-Trimethyl-2,6,10-dodecatrien-1-ol | Alcohols | RI, MS | <1 | n.d. | n.d. | n.d. | n.d. | n.d. | n.d. | n.d. | n.d. |
| 93 | 1311 | 112-44-7 | Undecanal | Aldehydes | RI, MS | <1 | n.d. | n.d. | n.d. | n.d. | n.d. | n.d. | n.d. | n.d. |
| 94 | 1318 | 67874-81-1 | 1-Oxaspiro[4.5]dec-6-ene, 2,6,10,10-tetramethyl- | Ketones | RI, MS | <1 | n.d. | n.d. | n.d. | n.d. | n.d. | n.d. | n.d. | n.d. |
| 95 | 1320 | 25152-84-5 | (E,E)-2,4-Decadienal | Ketones | RI, MS | 1.2021 | 1.03±0.28 | 1.12±0.06 | 1.47±0.07 | n.d. | n.d. | n.d. | n.d. | n.d. |
| 96 | 1321 | 514-81-2 | (Z)-1,5,9-Undecatriene, 2,6,10-trimethyl- | Alkenes | RI, MS | <1 | n.d. | n.d. | n.d. | n.d. | n.d. | n.d. | n.d. | n.d. |
| 97 | 1323 | 105-87-3 | trans-Geranic Acid Methyl Ester | Esters | RI, MS | <1 | n.d. | n.d. | 1.61±0.03 | n.d. | 1.7±0.1 | n.d. | 1.76±0.21 | 2.62±0.11 |
| 98 | 1347 | 6846-50-0 | 2,2,4-Trimethyl-1,3-pentanediol Diisobutyrate | Esters | RI, MS | <1 | n.d. | 0.92±0.06 | 0.48±0.07 | 0.66±0.49 | n.d. | 0.5±0.11 | 0.55±0.07 | n.d. |
| 99 | 1349 | 17699-14-8 | α-Cubebene | Alkenes | RI, MS | <1 | n.d. | n.d. | n.d. | n.d. | 1.26±0.03 | n.d. | 0.65±0.01 | n.d. |
| 100 | 1352 | 544-63-8 | Naphthalene, 1,2-dihydro-1,1,6-trimethyl- | Ketones | RI, MS | 1.35362 | 0.46±0.07 | 0.36±0.21 | 1.79±0.14 | 0.31±0.06 | n.d. | n.d. | n.d. | n.d. |
| 101 | 1354 | 68916-26-7 | (E)-2,6-Octadienoic Acid, 3,7-dimethyl- | Acids | RI, MS | <1 | n.d. | n.d. | n.d. | n.d. | n.d. | n.d. | n.d. | n.d. |
| 102 | 1358 | 104-61-0 | 2(3H)-Furanone, dihydro-5-pentyl- | Others | RI, MS | <1 | n.d. | 0.72±0.01 | n.d. | 0.6±0.29 | n.d. | n.d. | n.d. | n.d. |
| 103 | 1363 | 124-17-4 | Ethanol, 2-(2-butoxyethoxy)-, acetate | Alcohols | RI, MS | <1 | n.d. | n.d. | n.d. | n.d. | n.d. | n.d. | n.d. | n.d. |
| 104 | 1372 | 77-36-1 | Propanoic Acid, 2-methyl-, 3-hydroxy-2,2,4-trimethylpentyl Ester | Acids | RI, MS | <1 | n.d. | n.d. | n.d. | n.d. | n.d. | n.d. | n.d. | n.d. |
| 105 | 1373 | 5096-71-9 | 2-Octenal, 2-butyl- | Acids | RI, MS | 1.13237 | 3.03±0.78 | 4.48±0.64 | 4.37±0.95 | 3.03±0.45 | n.d. | n.d. | n.d. | n.d. |
| 106 | 1375 | 143-15-7 | 2-Bromo dodecane | Alkanes | RI, MS | <1 | n.d. | n.d. | n.d. | n.d. | n.d. | 0.66±0.09 | n.d. | n.d. |
| 107 | 1380 | 2363-88-4 | 2-Buten-1-one, 1-(2,6,6-trimethyl-1,3-cyclohexadien-1-yl)-, (E)- | Others | RI, MS | 1.279 | n.d. | n.d. | n.d. | n.d. | n.d. | 1.27±0.02 | n.d. | n.d. |
| 108 | 1381 | 105-87-3 | Geranyl acetate | Esters | RI, MS | <1 | n.d. | n.d. | n.d. | n.d. | 1.7±0.1 | n.d. | 1.76±0.21 | 1.22±0.04 |
| 109 | 1384 | 33467-74-2 | (Z)-3-Hexen-1-yl hexanoate | Esters | RI, MS | <1 | n.d. | 15.68±4.09 | 12.19±1.57 | 11.19±2.2 | n.d. | n.d. | 3.61±0.21 | 1.21±0.13 |
| 110 | 1384 | 33467-74-2 | (Z)-4-Hexen-1-yl hexanoate | Esters | RI, MS | <1 | n.d. | n.d. | n.d. | n.d. | n.d. | 0.73±0.1 | n.d. | n.d. |
| 111 | 1389 | 16491-36-4 | cis-3-Hexenyl cis-3-hexenoate | Esters | RI, MS | <1 | n.d. | 0.67±0.01 | 0.77±0.09 | n.d. | n.d. | n.d. | n.d. | n.d. |
| 112 | 1391 | 6378-65-0 | Hexanoic acid, hexyl ester | Alcohols | RI, MS | 1.02545 | 1.38±0.67 | 4.75±0.53 | 4.34±0.34 | 2.41±0.84 | n.d. | n.d. | 0.84±0.08 | n.d. |
| 113 | 1395 | 488-10-8 | (Z)-Jasmone | Ketones | RI, MS | <1 | 9.42±0.46 | 11.7±0.32 | n.d. | 9.97±4.15 | 13.91±1.05 | 2.17±0.44 | 6.63±1.29 | 6.32±1.09 |
| 114 | 1418 | 112-54-9 | Dodecanal | Aldehydes | RI, MS | <1 | n.d. | 0.2±0.07 | n.d. | n.d. | n.d. | n.d. | n.d. | n.d. |
| 115 | 1425 | 87-44-5 | Caryophyllene | Alkenes | RI, MS | <1 | n.d. | n.d. | n.d. | n.d. | n.d. | n.d. | n.d. | n.d. |
| 116 | 1428 | 127-41-3 | α-Ionone | Others | RI, MS | 1.30697 | 1.4±0.29 | 1.33±0.21 | n.d. | 0.85±0.28 | n.d. | 1.09±0.04 | 0.9±0.05 | 0.65±0.01 |
| 117 | 1447 | 103-45-7 | Propanoic acid, 2-methyl-, 2-phenylethyl ester | Aldehydes | RI, MS | 1.29845 | 1.17±0.13 | 0.65±0.04 | 0.97±0.15 | 0.86±0 | n.d. | n.d. | n.d. | n.d. |
| 118 | 1456 | 16984-13-7 | 5,9-Undecadien-2-one, 6,10-dimethyl- | Aldehydes | RI, MS | 1.24872 | 2.47±0.26 | 2.36±0.15 | 2.33±0.15 | 1.94±0.78 | 0.78±0.05 | 1.18±0.32 | 1.1±0.1 | 2.05±0.07 |
| 119 | 1463 | 18794-84-8 | (E)-β-Farnesene | Aldehydes | RI, MS | 1.13971 | 0.81±0.07 | 0.84±0.14 | 1.19±0.03 | 0.4±0.38 | n.d. | n.d. | n.d. | n.d. |
| 120 | 1483 | 2363-88-4 | 4-(2,6,6-Trimethylcyclohexa-1,3-dienyl)but-3-en-2-one | Ketones | RI, MS | <1 | n.d. | 0.56±0.09 | 0.47±0.03 | n.d. | n.d. | n.d. | n.d. | n.d. |
| 121 | 1485 | 14901-07-6 | β-Lonone | Ketones | RI, MS | <1 | n.d. | 8.64±2.23 | 6.71±0.78 | 6.87±0.92 | n.d. | 5.54±0.23 | 5.08±0.88 | 2.91±0.25 |
| 122 | 1490 | 122-57-6 | 5-Methyl-2-phenyl-2-hexenal | Ketones | RI, MS | <1 | n.d. | n.d. | n.d. | n.d. | n.d. | n.d. | n.d. | n.d. |
| 123 | 1492 | 104-61-0 | 2H-Pyran-2-one, tetrahydro-6-(2-pentenyl)-, (Z)- | Others | RI, MS | 1.29651 | n.d. | 5.95±1.86 | 14.44±2.08 | 4.89±2.38 | 0.33±0.05 | n.d. | n.d. | n.d. |
| 124 | 1495 | 104-61-0 | 2H-Pyran-2-one, tetrahydro-6-pentyl- | Others | RI, MS | <1 | n.d. | 1.25±0.23 | 1.56±0.11 | n.d. | n.d. | n.d. | n.d. | n.d. |
| 125 | 1501 | 6750-60-3 | (3S,3aR,3bR,4S,7R,7aR)-4-Isopropyl-3,7-dimethyloctahydro-1H-cyclopenta[1,3]cyclopropa[1,2]benzen-3-ol | Alcohols | RI, MS | <1 | n.d. | n.d. | n.d. | n.d. | 0.4±0.08 | n.d. | n.d. | n.d. |
| 126 | 1510 | 128-37-0 | Butylated Hydroxytoluene | Others | RI, MS | <1 | n.d. | n.d. | n.d. | n.d. | n.d. | 0.29±0.03 | n.d. | n.d. |
| 127 | 1515 | 96-76-4 | 2,4-Di-tert-butylphenol | Others | RI, MS | <1 | 4.07±0.34 | 5.35±0.52 | 4.24±0.66 | 4.12±1.76 | n.d. | n.d. | 7.74±3.54 | 4.43±2.06 |
| 128 | 1526 | 489-39-4 | Isoledene | Alkenes | RI, MS | <1 | n.d. | n.d. | n.d. | n.d. | 1.37±0.12 | n.d. | 0.93±0.03 | n.d. |
| 129 | 1527 | 489-39-4 | 1-Isopropyl-4,7-dimethyl-1,2,3,5,6,8a-hexahydronaphthalene | Others | RI, MS | <1 | n.d. | n.d. | n.d. | n.d. | n.d. | n.d. | n.d. | n.d. |
| 130 | 1528 | 6813-99-0 | 2(4H)-Benzofuranone, 5,6,7,7a-tetrahydro-4,4,7a-trimethyl-, (R)- | Ketones | RI, MS | 1.10326 | 0.98±0.12 | 1.61±0.04 | 2.27±0.07 | n.d. | n.d. | n.d. | n.d. | n.d. |
| 131 | 1568 | 106-32-1 | Butanoic acid, 3,7-dimethyl-2,6-octadienyl ester, (E)- | Esters | RI, MS | <1 | n.d. | n.d. | n.d. | n.d. | 0.66±0.12 | n.d. | n.d. | n.d. |
| 132 | 1572 | 7212-44-4 | nerolidol | Alcohols | RI, MS | 1.30969 | 40.52±4.2 | 35.85±0.45 | 45.58±2.3 | 38.99±4.09 | 1.21±0.12 | n.d. | 4.11±0.12 | 2.09±0.12 |
| 133 | 1580 | 1139-30-6 | 3-Hexen-1-ol, benzoate, (Z)- | Ketones | RI, MS | 1.01396 | 1.06±0.29 | 1.56±0.63 | n.d. | 1.04±0.02 | n.d. | n.d. | n.d. | n.d. |
| 134 | 1580 | 3063-30-5 | 3-Hexen-1-ol benzoate | Esters | RI, MS | <1 | n.d. | n.d. | n.d. | n.d. | n.d. | n.d. | n.d. | n.d. |
| 135 | 1587 | 629-94-7 | N-HENEICOSANE | Alkanes | RI, MS | <1 | n.d. | n.d. | n.d. | n.d. | n.d. | n.d. | n.d. | 0.51±0.22 |
| 136 | 1588 | 1139-30-6 | Caryophyllene oxide | Others | RI, MS | <1 | n.d. | n.d. | n.d. | n.d. | n.d. | n.d. | n.d. | n.d. |
| 137 | 1588 | 84-66-2 | Diethyl Phthalate | Others | RI, MS | <1 | n.d. | n.d. | n.d. | n.d. | 1.19±0.26 | 0.43±0.01 | n.d. | n.d. |
| 138 | 1614 | 77-53-2 | Cedrol | Alcohols | RI, MS | <1 | n.d. | 0.48±0.13 | n.d. | n.d. | n.d. | 0.44±0.07 | 0.28±0.11 | n.d. |
| 139 | 1655 | 71735-74-5 | Cyclopentaneacetic acid, 3-oxo-2-(2-pentenyl)-, methyl ester, [1.alpha.,2.alpha.(Z)]- | Esters | RI, MS | <1 | n.d. | 0.73±0.12 | n.d. | n.d. | n.d. | n.d. | n.d. | n.d. |
| 140 | 1661 | 1119-40-0 | Cyclopentaneacetic acid, 3-oxo-2-pentyl-, methyl ester | Esters | RI, MS | <1 | n.d. | n.d. | n.d. | n.d. | n.d. | 14.28±1.13 | n.d. | n.d. |
| 141 | 1666 | 1474790 | α-Cadinol | Alcohols | RI, MS | <1 | n.d. | n.d. | n.d. | n.d. | 0.8±0.43 | 0.5±0.12 | n.d. | n.d. |
| 142 | 1769 | 112-06-1 | neryl hexanoate | Esters | RI, MS | <1 | n.d. | n.d. | n.d. | n.d. | 0.42±0.01 | n.d. | n.d. | n.d. |
| 143 | 1798 | 6458-57-7 | 2-Hexyl-1-decano | Alkanes | RI, MS | <1 | n.d. | n.d. | n.d. | n.d. | n.d. | n.d. | n.d. | n.d. |
| 144 | 1843 | 1937-52-0 | 2-Pentadecanone, 6,10,14-trimethyl- | Ketones | RI, MS | <1 | 0.41±0.02 | 0.29±0.07 | n.d. | n.d. | n.d. | 0.18±0.03 | n.d. | n.d. |
| 145 | 1945 | 112-39-0 | Hexadecanoic acid, methyl ester | Acids | RI, MS | <1 | 0.64±0.43 | 0.55±0.09 | 0.49±0.03 | 1.08±1.13 | 0.5±0.12 | 0.44±0.02 | 0.43±0.06 | 0.29±0.1 |
| 146 | 1960 | 57-10-3 | n-Hexadecanoic acid | Acids | RI, MS | <1 | 0.18±0.06 | 0.15±0.01 | 0.62±0.09 | 0.51±0.44 | 0.42±0.42 | 0.25±0.1 | 0.34±0.04 | n.d. |
| 147 | 1985 | 84-74-2 | Dibutyl phthalate | Others | RI, MS | <1 | n.d. | n.d. | n.d. | n.d. | 0.06±0.01 | n.d. | n.d. | n.d. |

| **No.** | **RI** | **CAS** | **Compounds** | **Types** | **Identification basis** | | **VIP** | **Content (ug/L)** | | | | |
| --- | --- | --- | --- | --- | --- | --- | --- | --- | --- | --- | --- | --- |
|  |  |  |  |  |  |  |  | **SC1** | **SC2** | **SC3** | **SC5** | **SC9** |
| 1 | 698 | 64-19-7 | Acetic acid | Acids | | RI, MS | <1 | n.d. | n.d. | n.d. | 0.11±0.02 | n.d. |
| 2 | 744 | 110-05-4 | Di-tert-butyl peroxide | Others | | RI, MS | <1 | n.d. | n.d. | n.d. | n.d. | n.d. |
| 3 | 801 | 66-25-1 | Hexanal | Aldehydes | | RI, MS | 1.3359 | n.d. | 2.56±0.58 | 0.92±0.05 | 1.77±0.07 | n.d. |
| 4 | 828 | 98-01-1 | Furfural | Aldehydes | | RI, MS | 1.18407 | n.d. | n.d. | n.d. | n.d. | n.d. |
| 5 | 845 | 505-57-7 | 2-Hexenal | Aldehydes | | RI, MS | <1 | n.d. | n.d. | 5.02±0.15 | n.d. | 1.49±0.23 |
| 6 | 850 | 6728-26-3 | (E)-2-Hexenal | Aldehydes | | RI, MS | <1 | n.d. | 4.75±0.02 | n.d. | n.d. | n.d. |
| 7 | 852 | 33467-74-2 | (Z)-3-Hexen-1-ol formate | Alcohols | | RI, MS | <1 | n.d. | n.d. | n.d. | n.d. | n.d. |
| 8 | 853 | 928-96-1 | (E)-3-Hexen-1-ol | Alcohols | | RI, MS | <1 | n.d. | n.d. | n.d. | n.d. | n.d. |
| 9 | 863 | 928-95-0 | (E)-2-Hexen-1-ol | Alcohols | | RI, MS | <1 | n.d. | n.d. | n.d. | n.d. | n.d. |
| 10 | 867 | 111-27-3 | 1-Hexanol | Ketones | | RI, MS | 1.12692 | n.d. | n.d. | n.d. | n.d. | 0.24±0.11 |
| 11 | 889 | 110-43-0 | 2-Heptanone | Ketones | | RI, MS | <1 | n.d. | n.d. | n.d. | n.d. | n.d. |
| 12 | 901 | 111-71-7 | Heptanal | Aldehydes | | RI, MS | <1 | n.d. | n.d. | n.d. | n.d. | n.d. |
| 13 | 909 | 142-83-6 | (E,E)-2,4-Hexadienal | Aldehydes | | RI, MS | <1 | n.d. | n.d. | n.d. | n.d. | n.d. |
| 14 | 909 | 3208-16-0 | 2-Ethylfuran | Others | | RI, MS | <1 | n.d. | n.d. | n.d. | n.d. | n.d. |
| 15 | 918 | 100-51-6 | Nerolidol | Alkenes | | RI, MS | 1.07399 | 1.39±0.08 | 1.34±0.15 | 1.16±0.06 | 1.24±0.1 | 1.22±0.08 |
| 16 | 927 | 110-13-4 | 2,5-Hexanedione | Ketones | | RI, MS | <1 | n.d. | n.d. | n.d. | n.d. | n.d. |
| 17 | 954 | 18829-55-5 | (E)-2-Heptenal | Others | | RI, MS | 1.21947 | n.d. | 0.62±0.04 | n.d. | 0.31±0.08 | 0.78±0.06 |
| 18 | 958 | 100-52-7 | Benzaldehyde | Esters | | RI, MS | 1.10782 | 44.34±2.67 | 54.48±3.88 | 43.37±6.06 | 53.8±3.06 | 54.07±2.04 |
| 19 | 970 | 111-70-6 | 1-Heptanol | Alcohols | | RI, MS | <1 | n.d. | n.d. | n.d. | n.d. | n.d. |
| 20 | 976 | 4312-99-6 | 1-Octen-3-one | Ketones | | RI, MS | <1 | n.d. | n.d. | n.d. | n.d. | 0.38±0.1 |
| 21 | 979 | 3391-86-4 | 1-Octen-3-ol | Alkenes | | RI, MS | 1.25923 | 0.38±0.2 | n.d. | 0.26±0.06 | 1.25±0.26 | 1.66±0.04 |
| 22 | 983 | 110-93-0 | 6-Methyl-5-hepten-2-one | Aldehydes | | RI, MS | 1.14005 | n.d. | n.d. | n.d. | 1.93±0.03 | 1.66±0.03 |
| 23 | 989 | 123-35-3 | β-Myrcene | Ketones | | RI, MS | 1.11163 | 11.23±1.71 | 7.77±1.52 | 10.69±2.14 | 9.64±1.68 | 14.47±3.24 |
| 24 | 995 | 4313-03-5 | (E,E)-2,4-Heptadienal | Aldehydes | | RI, MS | 1.12057 | 0.63±0.07 | 3.07±0.19 | 1.96±0.11 | 0.35±0.07 | 0.66±0.01 |
| 25 | 1002 | 124-13-0 | Octanal | Aldehydes | | RI, MS | <1 | 0.35±0.98 | 1.23±0.08 | 0.29±0.14 | 0.34±0.03 | 0.1±0.03 |
| 26 | 1024 | 99-87-6 | p-Cymene | Alkenes | | RI, MS | <1 | 0.58±0.71 | 0.6±0.04 | n.d. | n.d. | n.d. |
| 27 | 1029 | 5989-27-5 | D-Limonene | Alkenes | | RI, MS | <1 | n.d. | n.d. | n.d. | n.d. | n.d. |
| 28 | 1032 | 100-51-6 | Benzyl alcohol | Aldehydes | | RI, MS | 1.34865 | 42.72±1.05 | 44.59±2.72 | 40.66±6.77 | 50.19±2.02 | 52.94±5.7 |
| 29 | 1037 | 3779-61-1 | trans-β-Ocimene | Alkenes | | RI, MS | <1 | n.d. | n.d. | n.d. | n.d. | n.d. |
| 30 | 1037 | 1669-44-9 | 3-Octen-2-one | Others | | RI, MS | 1.10109 | n.d. | n.d. | n.d. | n.d. | n.d. |
| 31 | 1042 | 122-78-1 | benzeneacetaldehyde | Aldehydes | | RI, MS | 1.07435 | 46.99±3.49 | 49.72±4.11 | 38.43±6.28 | 50.58±1.74 | 43.22±1.51 |
| 32 | 1046 | 2167-14-8 | 1-Ethyl-1H-pyrrole-2-carboxaldehyde | Ketones | | RI, MS | <1 | n.d. | n.d. | 4.96±0.9 | n.d. | 8.04±0.42 |
| 33 | 1047 | 13877-91-3 | β-Ocimene | Alkenes | | RI, MS | <1 | n.d. | n.d. | n.d. | n.d. | n.d. |
| 34 | 1048 | 68917-11-3 | Basilene (isomer mixture) | Alkenes | | RI, MS | <1 | 2.47±0.16 | n.d. | n.d. | n.d. | n.d. |
| 35 | 1058 | 2548-87-0 | (E)-2-Octenal | Aldehydes | | RI, MS | 1.32469 | 1.49±0.05 | 1.68±0.09 | 1.37±0.37 | 1.13±0.66 | 1.26±0.15 |
| 36 | 1064 | 1072-83-9 | 1-(1H-Pyrrol-2-yl)ethanone | Ketones | | RI, MS | <1 | n.d. | n.d. | n.d. | n.d. | 0.41±0.19 |
| 37 | 1064 | 98-86-2 | 2-acetyl pyrrole | Ketones | | RI, MS | <1 | n.d. | n.d. | n.d. | n.d. | n.d. |
| 38 | 1069 | 1669-44-9 | 3,5-Octadien-2-one | Ketones | | RI, MS | 1.28684 | 1.46±0.06 | 4.43±0.58 | 2.96±0.49 | 5.8±0.15 | 2.69±0.35 |
| 39 | 1072 | 1128-16-1 | cis-2,5,5-Trimethyl-2-vinyltetrahydrofuran | Others | | RI, MS | <1 | n.d. | n.d. | n.d. | 12.35±0.01 | 10.51±3.06 |
| 40 | 1072 | 1365-19-1 | trans-Linalool oxide (furanoid) | Aldehydes | | RI, MS | 1.2669 | 17.59±0.3 | 23.75±2.52 | 18.75±6.25 | 34.46±1.76 | 24.36±1.97 |
| 41 | 1079 | 111-14-8 | Heptanoic acid | Aldehydes | | RI, MS | 1.00376 | n.d. | n.d. | n.d. | 0.17±0.06 | n.d. |
| 42 | 1101 | 78-70-6 | Linalool | Alcohols | | RI, MS | <1 | 25.42±0.4 | 33.59±0.5 | 35.53±0.15 | 40.98±5.07 | 44.28±5.61 |
| 43 | 1104 | 78-70-6 | 3,7-Dimethyl-1,5,7-octatrien-3-ol | Alcohols | | RI, MS | <1 | n.d. | n.d. | n.d. | 13.53±2.01 | 11.23±2.13 |
| 44 | 1105 | 124-19-6 | Nonanal | Aldehydes | | RI, MS | <1 | n.d. | 10.88±0.66 | 6±0.1 | n.d. | n.d. |
| 45 | 1110 | 60-12-8 | Phenylethyl Alcohol | Alcohols | | RI, MS | <1 | 5.69±0.58 | 13.84±0.01 | 10.06±0.1 | 23.98±8.04 | 20.68±5.28 |
| 46 | 1113 | 141-13-9 | (E)-4,8-Dimethylnona-1,3,7-triene | Alkenes | | RI, MS | <1 | n.d. | n.d. | n.d. | n.d. | n.d. |
| 47 | 1128 | 2049-96-9 | (E,Z)-2,6-Dimethyl-2,4,6-octatriene | Alkenes | | RI, MS | <1 | n.d. | n.d. | n.d. | n.d. | n.d. |
| 48 | 1128 | 2049-96-9 | 2,6-Dimethyl-2,4,6-octatriene | Alkenes | | RI, MS | <1 | 0.89±0.03 | n.d. | n.d. | n.d. | n.d. |
| 49 | 1133 | 140-29-4 | Benzyl Nitrile | Aldehydes | | RI, MS | 1.20135 | n.d. | n.d. | n.d. | 0.56±0.16 | n.d. |
| 50 | 1136 | 16433-88-8 | trans-3-Nonen-2-one | Ketones | | RI, MS | <1 | n.d. | n.d. | 0.13±0.02 | 0.32±0.04 | n.d. |
| 51 | 1148 | 104-53-0 | Lilac Aldehyde B | Aldehydes | | RI, MS | <1 | n.d. | n.d. | n.d. | n.d. | n.d. |
| 52 | 1150 | 557-48-2 | (E,Z)-2,6-Nonadienal | Aldehydes | | RI, MS | 1.33696 | n.d. | n.d. | n.d. | n.d. | n.d. |
| 53 | 1152 | 59355-75-4 | (Z)-3-Nonen-1-ol | Alcohols | | RI, MS | <1 | n.d. | 0.66±0.01 | 0.86±0.18 | 0.53±0.06 | 0.79±0.06 |
| 54 | 1155 | 59355-75-4 | (E,Z)-3,6-Nonadien-1-ol | Alcohols | | RI, MS | <1 | n.d. | n.d. | n.d. | n.d. | n.d. |
| 55 | 1158 | 18829-56-6 | (E)-2-Nonenal | Ketones | | RI, MS | 1.27497 | 0.5±0.03 | 0.99±0.09 | 0.69±0.06 | 0.5±0.07 | 0.37±0.04 |
| 56 | 1161 | 140-11-4 | Benzyl Acetate | Esters | | RI, MS | <1 | n.d. | n.d. | n.d. | n.d. | 0.85±0.06 |
| 57 | 1169 | 16409-43-1 | (3R,6S)-2,2,6-Trimethyl-6-vinyltetrahydro-2H-pyran-3-ol | Alcohols | | RI, MS | <1 | 2.31±0.04 | 1.3±0.03 | 3.65±0.62 | 13.12±0.09 | 10.99±2.06 |
| 58 | 1172 | 143-08-8 | 1-Nonanol | Alcohols | | RI, MS | <1 | 2.94±0.3 | 1.59±0.07 | 2.65±0.38 | n.d. | n.d. |
| 59 | 1178 | 1490-04-6 | dl-Menthol | Alcohols | | RI, MS | <1 | n.d. | 0.71±0.01 | 0.53±0.01 | n.d. | n.d. |
| 60 | 1179 | 2216-51-5 | Levomenthol | Alcohols | | RI, MS | <1 | n.d. | n.d. | n.d. | n.d. | n.d. |
| 61 | 1181 | 562-74-3 | Terpinen-4-ol | Others | | RI, MS | 1.25981 | n.d. | 0.24±0.02 | 0.18±0.09 | n.d. | n.d. |
| 62 | 1185 | 91-20-3 | Naphthalene | Alkenes | | RI, MS | <1 | n.d. | n.d. | n.d. | n.d. | n.d. |
| 63 | 1187 | 16409-43-1 | (E)-3-Hexenyl Butanoate | Alcohols | | RI, MS | 1.03192 | n.d. | n.d. | n.d. | n.d. | n.d. |
| 64 | 1187 | 67674-46-8 | cis-3-Hexenyl Isobutyrate | Esters | | RI, MS | <1 | 0.45±0.07 | n.d. | 0.48±0.08 | 1.19±0.06 | 1.13±0.07 |
| 65 | 1187 | 16409-43-1 | (Z)-3-Hexenyl Butanoate | Esters | | RI, MS | <1 | n.d. | n.d. | n.d. | n.d. | n.d. |
| 66 | 1194 | 119-36-8 | Methyl Salicylate | Esters | | RI, MS | 1.25782 | 44.27±2.11 | 37.88±3.22 | 40.85±2.59 | 78.73±0.79 | 84.96±7 |
| 67 | 1198 | 98-55-5 | α-Terpineol | Esters | | RI, MS | 1.06755 | 2.19±0.08 | 1.65±0.39 | 1.42±0.51 | 3.81±0.03 | 3.62±0.44 |
| 68 | 1198 | 98-55-5 | L-α-Terpineol | Alcohols | | RI, MS | <1 | n.d. | n.d. | n.d. | n.d. | n.d. |
| 69 | 1201 | 432-25-7 | 2,6,6-Trimethyl-1-cyclohexadiene-1-carboxaldehyde | Alcohols | | RI, MS | 1.18762 | n.d. | n.d. | n.d. | n.d. | n.d. |
| 70 | 1208 | 112-31-2 | Decanal | Esters | | RI, MS | 1.2538 | 1.96±0.96 | 1.27±0.12 | 1.07±0.51 | 1.07±0.01 | 0.72±0.11 |
| 71 | 1217 | 5910-87-2 | (E,E)-2,4-Nonadienal | Alcohols | | RI, MS | 1.0351 | n.d. | n.d. | n.d. | n.d. | n.d. |
| 72 | 1221 | 432-25-7 | 2,6,6-Trimethyl-1-cyclohexene-1-carboxaldehyde | Aldehydes | | RI, MS | <1 | 2.17±0.36 | n.d. | n.d. | n.d. | n.d. |
| 73 | 1227 | 106-25-2 | nerol | Aldehydes | | RI, MS | 1.30751 | 3.52±0.14 | 1.44±0.58 | 1.85±0.2 | 11.84±0.94 | 6.68±0.4 |
| 74 | 1231 | 80-56-8 | 1,7,7-Trimethylbicyclo[2.2.1]hept-2-ene | Alkenes | | RI, MS | <1 | n.d. | 0.84±0.55 | 0.61±0.03 | n.d. | n.d. |
| 75 | 1233 | 67674-46-8 | cis-3-Hexenyl α-Methylbutyrate | Aldehydes | | RI, MS | 1.3142 | 0.98±0 | n.d. | 0.63±0.05 | 1.39±0.22 | 0.46±0.02 |
| 76 | 1238 | 67674-46-8 | (E,Z)-3-Hexenyl 2-Butenoate | Esters | | RI, MS | <1 | n.d. | n.d. | n.d. | n.d. | 0.16±0.01 |
| 77 | 1238 | 67674-46-8 | cis-3-Hexenyl Isovalerate | Esters | | RI, MS | <1 | n.d. | n.d. | 0.51±0.21 | n.d. | n.d. |
| 78 | 1240 | 106-26-3 | Neral | Aldehydes | | RI, MS | 1.1463 | 2.87±0.42 | 2.32±0.33 | n.d. | 6.5±3.77 | 5.23±0.1 |
| 79 | 1253 | 106-24-1 | Geraniol | Alcohols | | RI, MS | 1.37991 | 734.47±17 | 804.05±15.3 | 843.03±30.47 | 1680.28±23.23 | 1025.3±21.58 |
| 80 | 1257 | 432-25-7 | 1-Cyclohexene-1-acetaldehyde, 2,6,6-trimethyl- | Aldehydes | | RI, MS | <1 | n.d. | n.d. | n.d. | n.d. | n.d. |
| 81 | 1261 | 25101-02-4 | Pentanoic acid, 2-methyl-, anhydride | Acids | | RI, MS | <1 | n.d. | n.d. | n.d. | n.d. | n.d. |
| 82 | 1264 | 3913-81-3 | (E)-2-Decenal | Alcohols | | RI, MS | 1.30969 | n.d. | n.d. | n.d. | n.d. | n.d. |
| 83 | 1270 | 5392-40-5 | Citral | Esters | | RI, MS | 1.08292 | n.d. | n.d. | n.d. | n.d. | n.d. |
| 84 | 1270 | 141-27-5 | (E)-citral trans-3,7-dimethylocta-2,6-dienal | Esters | | RI, MS | 1.32215 | 16.07±2.3 | 14.19±1.53 | n.d. | 19.32±5.56 | 19.73±0.3 |
| 85 | 1274 | 112-05-0 | Nonanoic Acid | Acids | | RI, MS | <1 | n.d. | 0.82±0.22 | n.d. | n.d. | n.d. |
| 86 | 1294 | 120-72-9 | Indole | Alcohols | | RI, MS | 1.22019 | n.d. | n.d. | n.d. | n.d. | n.d. |
| 87 | 1295 | 536-60-7 | p-Cymen-7-ol | Alcohols | | RI, MS | <1 | n.d. | n.d. | n.d. | 0.24±0.03 | n.d. |
| 88 | 1295 | 89-83-8 | Phenol, 2-methyl-5-(1-methylethyl)- | Alcohols | | RI, MS | <1 | n.d. | n.d. | n.d. | n.d. | n.d. |
| 89 | 1300 | 612-22-6 | Benzene, (2-nitroethyl)- | Alcohols | | RI, MS | 1.19049 | n.d. | n.d. | n.d. | n.d. | n.d. |
| 90 | 1302 | 105-86-2 | (Z)-2,6-Octadien-1-ol, 3,7-dimethyl-, formate | Alcohols | | RI, MS | <1 | n.d. | n.d. | n.d. | n.d. | n.d. |
| 91 | 1303 | 105-86-2 | Geranyl Formate | Alcohols | | RI, MS | <1 | n.d. | n.d. | n.d. | 0.82±0.12 | n.d. |
| 92 | 1303 | 111-02-4 | 3,7,11-Trimethyl-2,6,10-dodecatrien-1-ol | Alcohols | | RI, MS | <1 | n.d. | n.d. | n.d. | n.d. | n.d. |
| 93 | 1311 | 112-44-7 | Undecanal | Aldehydes | | RI, MS | <1 | n.d. | 0.16±0.03 | n.d. | n.d. | n.d. |
| 94 | 1318 | 67874-81-1 | 1-Oxaspiro[4.5]dec-6-ene, 2,6,10,10-tetramethyl- | Ketones | | RI, MS | <1 | n.d. | n.d. | n.d. | n.d. | n.d. |
| 95 | 1320 | 25152-84-5 | (E,E)-2,4-Decadienal | Ketones | | RI, MS | 1.2021 | n.d. | n.d. | n.d. | n.d. | n.d. |
| 96 | 1321 | 514-81-2 | (Z)-1,5,9-Undecatriene, 2,6,10-trimethyl- | Alkenes | | RI, MS | <1 | n.d. | n.d. | n.d. | n.d. | n.d. |
| 97 | 1323 | 105-87-3 | trans-Geranic Acid Methyl Ester | Esters | | RI, MS | <1 | 0.33±0.04 | 0.34±0.02 | 0.39±0.09 | n.d. | n.d. |
| 98 | 1347 | 6846-50-0 | 2,2,4-Trimethyl-1,3-pentanediol Diisobutyrate | Esters | | RI, MS | <1 | 0.26±0.12 | n.d. | 0.32±0.02 | 0.2±0.09 | 0.26±0.04 |
| 99 | 1349 | 17699-14-8 | α-Cubebene | Alkenes | | RI, MS | <1 | n.d. | n.d. | n.d. | 0.78±0.06 | n.d. |
| 100 | 1352 | 544-63-8 | Naphthalene, 1,2-dihydro-1,1,6-trimethyl- | Ketones | | RI, MS | 1.35362 | n.d. | n.d. | n.d. | n.d. | n.d. |
| 101 | 1354 | 68916-26-7 | (E)-2,6-Octadienoic Acid, 3,7-dimethyl- | Acids | | RI, MS | <1 | n.d. | n.d. | n.d. | 3.76±0.22 | n.d. |
| 102 | 1358 | 104-61-0 | 2(3H)-Furanone, dihydro-5-pentyl- | Others | | RI, MS | <1 | n.d. | n.d. | n.d. | n.d. | n.d. |
| 103 | 1363 | 124-17-4 | Ethanol, 2-(2-butoxyethoxy)-, acetate | Alcohols | | RI, MS | <1 | n.d. | 0.19±0.07 | n.d. | n.d. | n.d. |
| 104 | 1372 | 77-36-1 | Propanoic Acid, 2-methyl-, 3-hydroxy-2,2,4-trimethylpentyl Ester | Acids | | RI, MS | <1 | n.d. | n.d. | n.d. | n.d. | n.d. |
| 105 | 1373 | 5096-71-9 | 2-Octenal, 2-butyl- | Acids | | RI, MS | 1.13237 | n.d. | n.d. | n.d. | n.d. | n.d. |
| 106 | 1375 | 143-15-7 | 2-Bromo dodecane | Alkanes | | RI, MS | <1 | n.d. | n.d. | n.d. | n.d. | n.d. |
| 107 | 1380 | 2363-88-4 | 2-Buten-1-one, 1-(2,6,6-trimethyl-1,3-cyclohexadien-1-yl)-, (E)- | Others | | RI, MS | 1.279 | n.d. | n.d. | n.d. | n.d. | n.d. |
| 108 | 1381 | 105-87-3 | Geranyl acetate | Esters | | RI, MS | <1 | n.d. | n.d. | n.d. | 1.62±0.19 | n.d. |
| 109 | 1384 | 33467-74-2 | (Z)-3-Hexen-1-yl hexanoate | Esters | | RI, MS | <1 | n.d. | n.d. | n.d. | n.d. | n.d. |
| 110 | 1384 | 33467-74-2 | (Z)-4-Hexen-1-yl hexanoate | Esters | | RI, MS | <1 | n.d. | 0.55±0.09 | 1.07±0.98 | n.d. | n.d. |
| 111 | 1389 | 16491-36-4 | cis-3-Hexenyl cis-3-hexenoate | Esters | | RI, MS | <1 | n.d. | n.d. | n.d. | n.d. | n.d. |
| 112 | 1391 | 6378-65-0 | Hexanoic acid, hexyl ester | Alcohols | | RI, MS | 1.02545 | n.d. | n.d. | 0.46±0.22 | n.d. | n.d. |
| 113 | 1395 | 488-10-8 | (Z)-Jasmone | Ketones | | RI, MS | <1 | n.d. | 1.2±0.62 | 1.91±0.59 | 5.56±0.27 | 5.88±0.31 |
| 114 | 1418 | 112-54-9 | Dodecanal | Aldehydes | | RI, MS | <1 | n.d. | n.d. | n.d. | n.d. | n.d. |
| 115 | 1425 | 87-44-5 | Caryophyllene | Alkenes | | RI, MS | <1 | n.d. | n.d. | n.d. | n.d. | n.d. |
| 116 | 1428 | 127-41-3 | α-Ionone | Others | | RI, MS | 1.30697 | 1.18±0.11 | 0.82±0.01 | 0.6±0.24 | 0.57±0.05 | n.d. |
| 117 | 1447 | 103-45-7 | Propanoic acid, 2-methyl-, 2-phenylethyl ester | Aldehydes | | RI, MS | 1.29845 | n.d. | n.d. | n.d. | 0.3±0.02 | n.d. |
| 118 | 1456 | 16984-13-7 | 5,9-Undecadien-2-one, 6,10-dimethyl- | Aldehydes | | RI, MS | 1.24872 | 0.58±0.48 | 1.29±0.16 | 1.51±0.88 | 0.96±0.11 | 0.53±0.03 |
| 119 | 1463 | 18794-84-8 | (E)-β-Farnesene | Aldehydes | | RI, MS | 1.13971 | n.d. | n.d. | n.d. | n.d. | n.d. |
| 120 | 1483 | 2363-88-4 | 4-(2,6,6-Trimethylcyclohexa-1,3-dienyl)but-3-en-2-one | Ketones | | RI, MS | <1 | n.d. | n.d. | n.d. | n.d. | n.d. |
| 121 | 1485 | 14901-07-6 | β-Lonone | Ketones | | RI, MS | <1 | 5.17±0.06 | 4.44±2.1 | 5.46±0.24 | n.d. | 1.58±0.81 |
| 122 | 1490 | 122-57-6 | 5-Methyl-2-phenyl-2-hexenal | Ketones | | RI, MS | <1 | n.d. | n.d. | n.d. | n.d. | n.d. |
| 123 | 1492 | 104-61-0 | 2H-Pyran-2-one, tetrahydro-6-(2-pentenyl)-, (Z)- | Others | | RI, MS | 1.29651 | n.d. | n.d. | n.d. | n.d. | n.d. |
| 124 | 1495 | 104-61-0 | 2H-Pyran-2-one, tetrahydro-6-pentyl- | Others | | RI, MS | <1 | n.d. | n.d. | n.d. | n.d. | n.d. |
| 125 | 1501 | 6750-60-3 | (3S,3aR,3bR,4S,7R,7aR)-4-Isopropyl-3,7-dimethyloctahydro-1H-cyclopenta[1,3]cyclopropa[1,2]benzen-3-ol | Alcohols | | RI, MS | <1 | n.d. | n.d. | n.d. | n.d. | n.d. |
| 126 | 1510 | 128-37-0 | Butylated Hydroxytoluene | Others | | RI, MS | <1 | n.d. | n.d. | n.d. | n.d. | n.d. |
| 127 | 1515 | 96-76-4 | 2,4-Di-tert-butylphenol | Others | | RI, MS | <1 | n.d. | n.d. | n.d. | 1.99±0.02 | n.d. |
| 128 | 1526 | 489-39-4 | Isoledene | Alkenes | | RI, MS | <1 | n.d. | n.d. | n.d. | 0.75±0.18 | n.d. |
| 129 | 1527 | 489-39-4 | 1-Isopropyl-4,7-dimethyl-1,2,3,5,6,8a-hexahydronaphthalene | Others | | RI, MS | <1 | n.d. | n.d. | n.d. | n.d. | n.d. |
| 130 | 1528 | 6813-99-0 | 2(4H)-Benzofuranone, 5,6,7,7a-tetrahydro-4,4,7a-trimethyl-, (R)- | Ketones | | RI, MS | 1.10326 | n.d. | 1.11±0.04 | n.d. | n.d. | n.d. |
| 131 | 1568 | 106-32-1 | Butanoic acid, 3,7-dimethyl-2,6-octadienyl ester, (E)- | Esters | | RI, MS | <1 | n.d. | n.d. | n.d. | n.d. | n.d. |
| 132 | 1572 | 7212-44-4 | nerolidol | Alcohols | | RI, MS | 1.30969 | 1.7±0.25 | 1.47±0.02 | 1.47±0.28 | 3.45±0.37 | 1.62±0.25 |
| 133 | 1580 | 1139-30-6 | 3-Hexen-1-ol, benzoate, (Z)- | Ketones | | RI, MS | 1.01396 | n.d. | n.d. | n.d. | n.d. | n.d. |
| 134 | 1580 | 3063-30-5 | 3-Hexen-1-ol benzoate | Esters | | RI, MS | <1 | n.d. | n.d. | n.d. | 0.5±0.09 | n.d. |
| 135 | 1587 | 629-94-7 | N-HENEICOSANE | Alkanes | | RI, MS | <1 | n.d. | n.d. | n.d. | n.d. | n.d. |
| 136 | 1588 | 1139-30-6 | Caryophyllene oxide | Others | | RI, MS | <1 | n.d. | n.d. | n.d. | n.d. | n.d. |
| 137 | 1588 | 84-66-2 | Diethyl Phthalate | Others | | RI, MS | <1 | 1.17±0.34 | 1.4±0.23 | 0.85±0.27 | n.d. | n.d. |
| 138 | 1614 | 77-53-2 | Cedrol | Alcohols | | RI, MS | <1 | n.d. | 1.72±0.04 | 0.5±0.26 | 0.47±0.05 | n.d. |
| 139 | 1655 | 71735-74-5 | Cyclopentaneacetic acid, 3-oxo-2-(2-pentenyl)-, methyl ester, [1.alpha.,2.alpha.(Z)]- | Esters | | RI, MS | <1 | n.d. | n.d. | n.d. | n.d. | n.d. |
| 140 | 1661 | 1119-40-0 | Cyclopentaneacetic acid, 3-oxo-2-pentyl-, methyl ester | Esters | | RI, MS | <1 | n.d. | 8.35±0.11 | 1.97±0.13 | n.d. | n.d. |
| 141 | 1666 | 1474790 | α-Cadinol | Alcohols | | RI, MS | <1 | n.d. | n.d. | n.d. | n.d. | n.d. |
| 142 | 1769 | 112-06-1 | neryl hexanoate | Esters | | RI, MS | <1 | n.d. | n.d. | n.d. | 0.26±0.01 | n.d. |
| 143 | 1798 | 6458-57-7 | 2-Hexyl-1-decano | Alkanes | | RI, MS | <1 | n.d. | n.d. | n.d. | n.d. | 0.45±0.06 |
| 144 | 1843 | 1937-52-0 | 2-Pentadecanone, 6,10,14-trimethyl- | Ketones | | RI, MS | <1 | n.d. | n.d. | n.d. | n.d. | n.d. |
| 145 | 1945 | 112-39-0 | Hexadecanoic acid, methyl ester | Acids | | RI, MS | <1 | 0.1±0.01 | 0.4±0.33 | 0.35±0.14 | 0.31±0.11 | 0.32±0.11 |
| 146 | 1960 | 481-34-5 | n-Hexadecanoic acid | Acids | | RI, MS | <1 | 0.12±0.08 | 0.1±0.02 | 0.17±0.03 | 0.91±0.09 | n.d. |
| 147 | 1985 | 84-74-2 | Dibutyl phthalate | Others | | RI, MS | <1 | n.d. | 0.07±0.03 | n.d. | n.d. | n.d. |

^a^ SC4, SC6, SC7 and SC8 refer to Table 2 in the main text respectively as B1, B2, B3, B4.

**Table S6. Standard Curves of Volatile Compounds.**

| **No.** | **Compounds** | **Standard curves** | **R²** | **Purity(%)** |
| --- | --- | --- | --- | --- |
| 1 | geraniol | y= 1377574.4059 x + 968409.0833 | 0.9972 | >98 |
| 2 | linalool | y = 314056.0410 x + 1025171.2917 | 0.9933 | >98 |
| 3 | benzeneacetaldehyde | y = 681169.7608 x + 329848.0833 | 0.9994 | >99 |
| 4 | beta-myrcene | y = 4006017.0027 x + 497738.5833 | 0.9995 | >90 |
| 5 | methyl salicylate | y = 3735170.3038 x - 1316852.1667 | 0.9982 | >98 |
| 6 | benzaldehyde | y = 2427293.7769 x + 3496208.5833 | 0.9940 | >98.5 |

**Figure S1. Aroma wheel of floral-fruity congou black tea.**

**
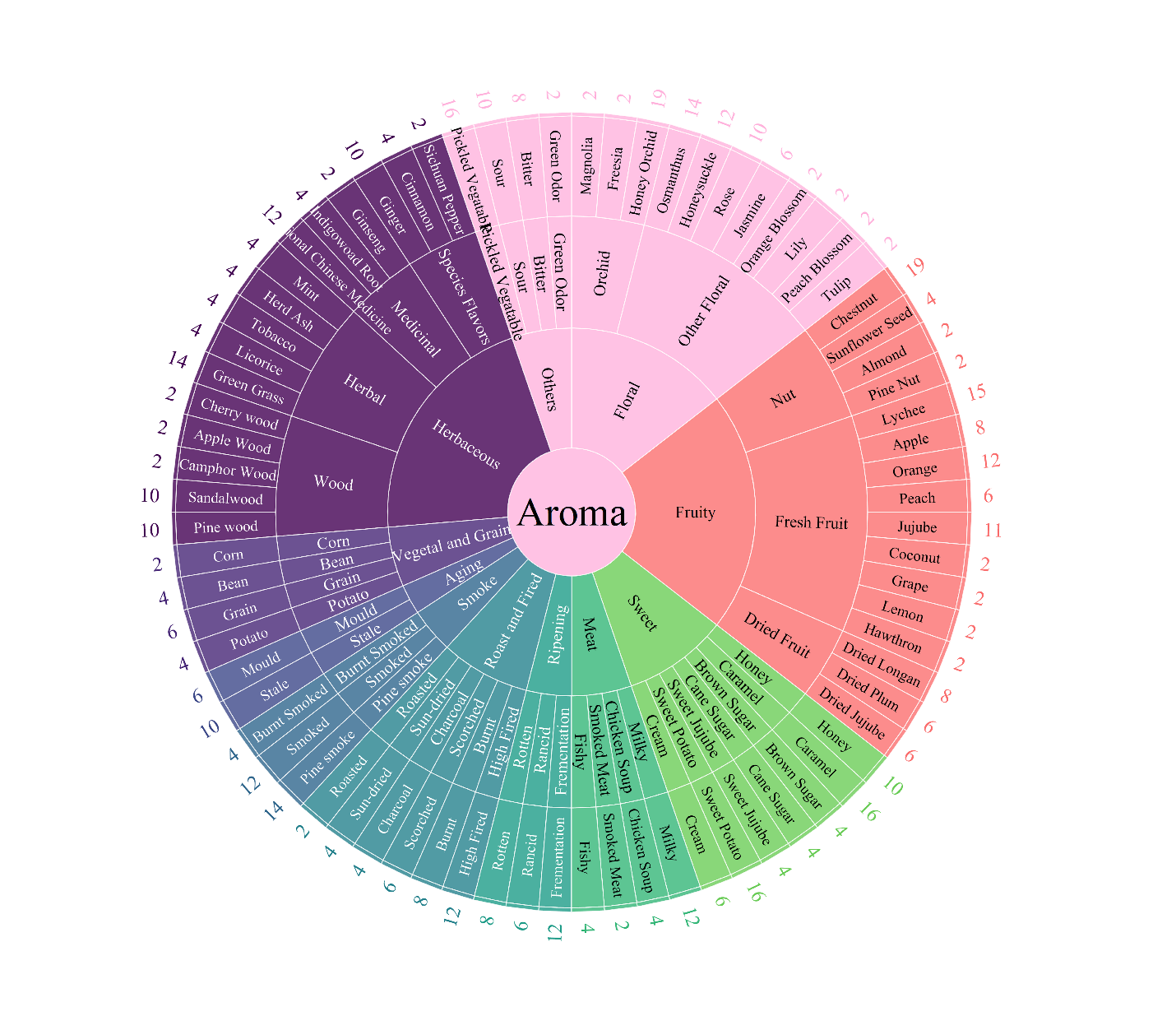
**

**Figure S2-S3. Panel performance testing in sensory analysis by PanelCheck 1.4.0**


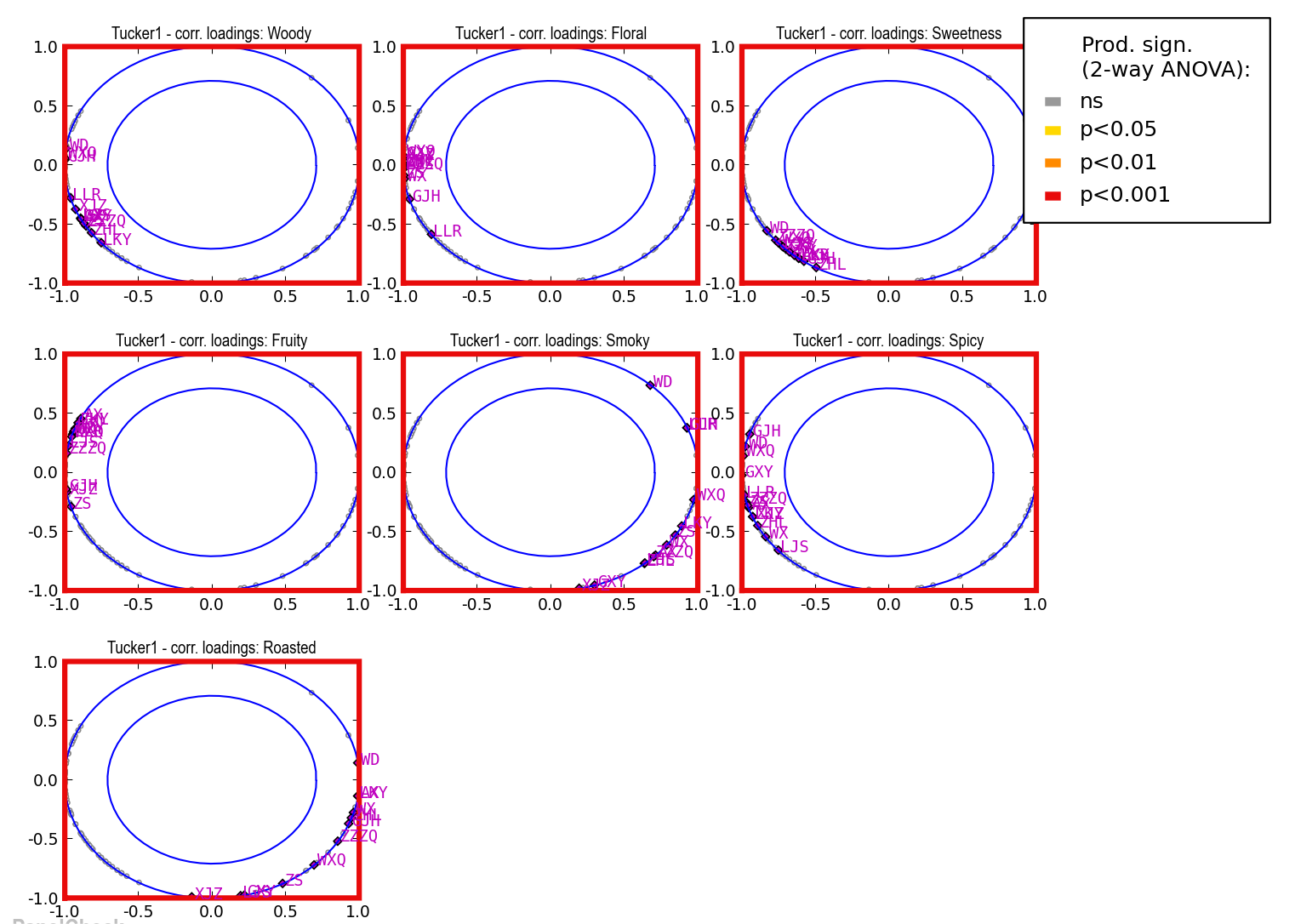


Figure S2 Seven identical Tucker-1 plots with each plot highlighting one of the nine attributes used in the profiling.

(a)


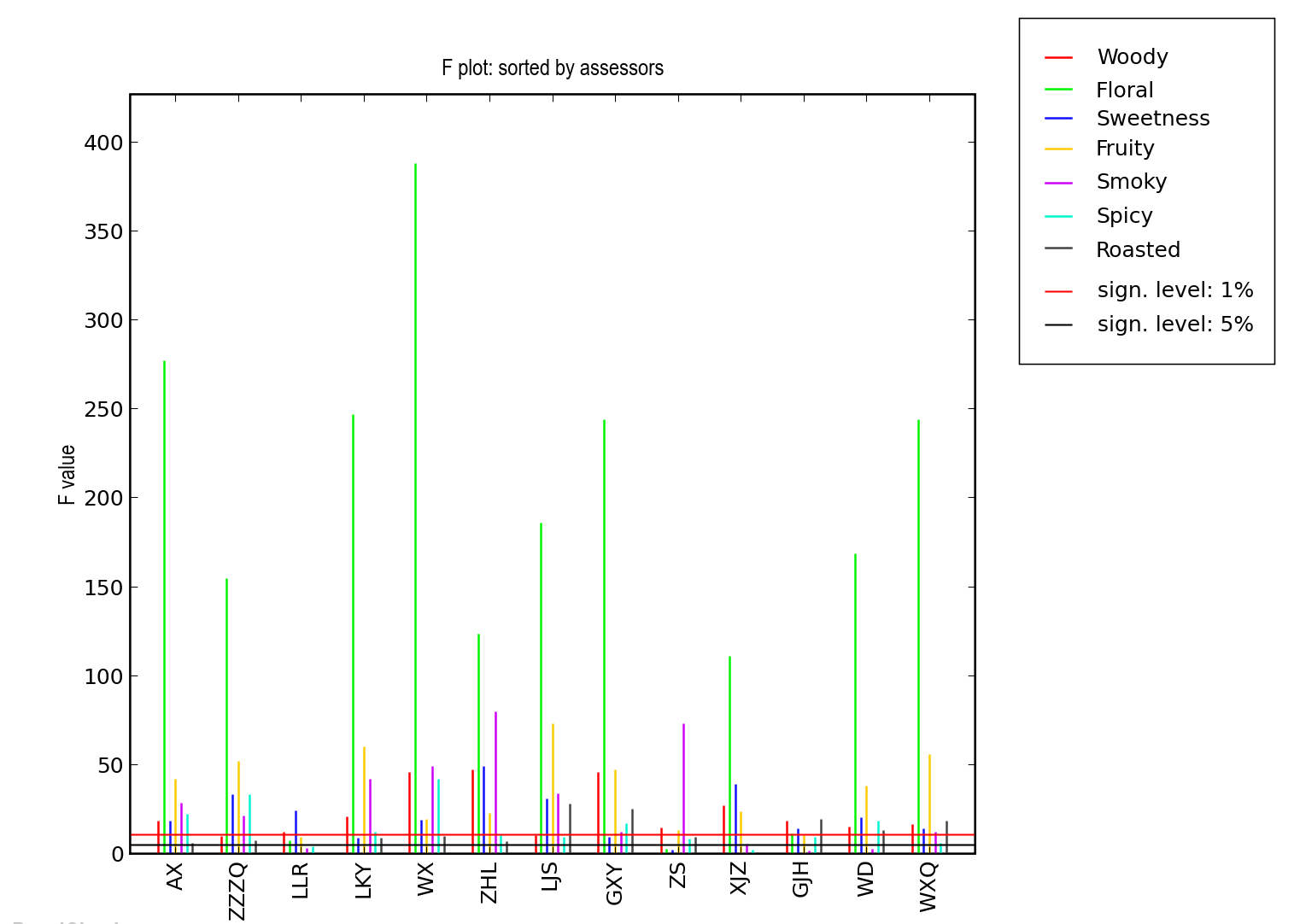


(b)


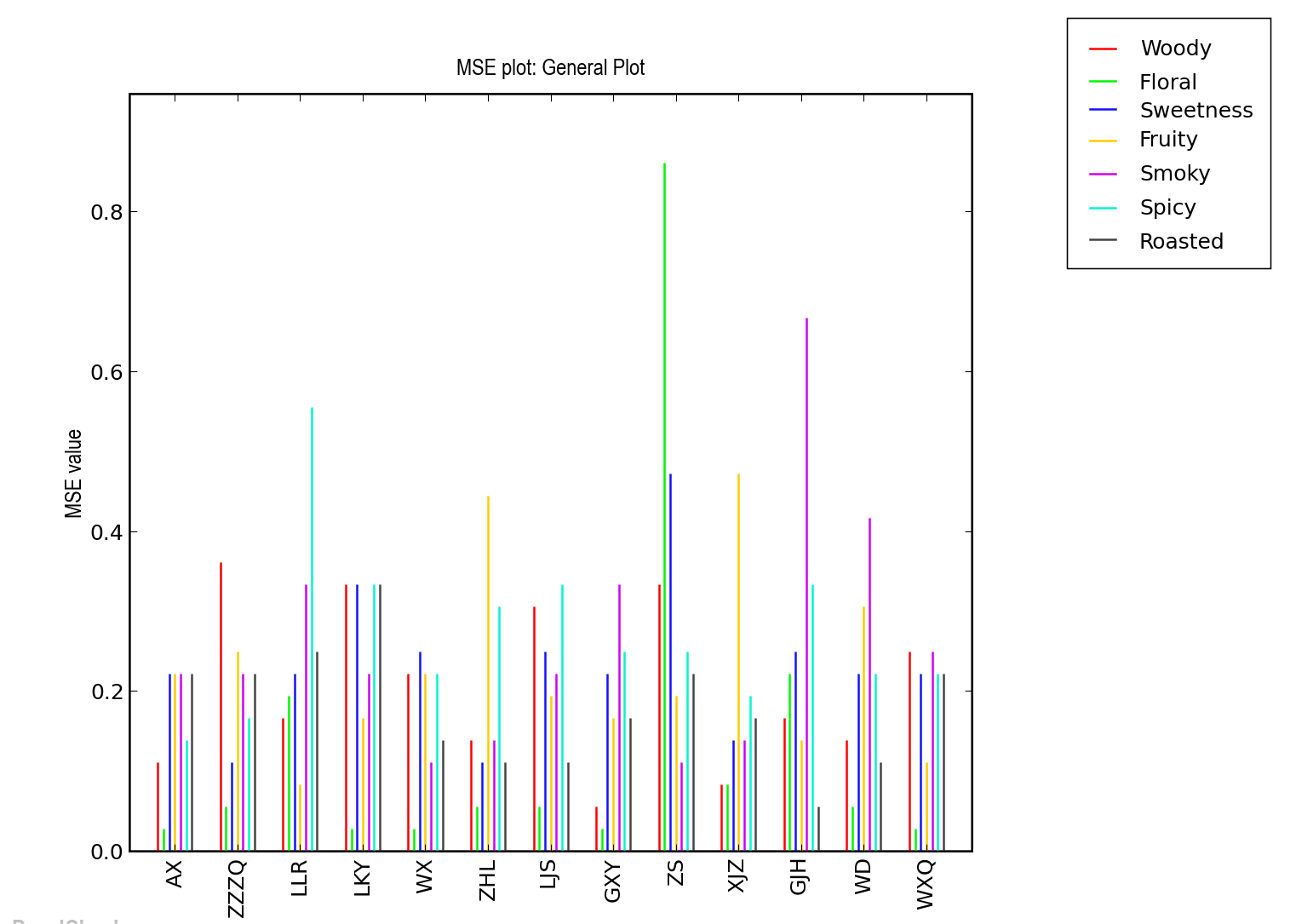


Figure S3 F plots (a) visualising the panels' ability to discriminate between the tested samples for each attribute. MSE plot (b) visualising the repeatability of each panel.

Most panels have relatively low MSE (good repeatability) values combined with relatively high F values (good sample discrimination). 3 panels (LLR, ZS, GJH) with poor discrimination ability were removed, and a total of 10 panels were screened for subsequent analysis.
