# Supplementary material for: Key floral-fruity aroma compounds in Sichuan Congou black tea: identification via MDGC-MS/O and sensory evaluation
Source: Front Nutr. 2025 May 2;12:1577302. doi: 10.3389/fnut.2025.1577302 (PMC12083010; doi:10.3389/fnut.2025.1577302)
Supplement: Supplementary file 2 [file Image_1.pdf]

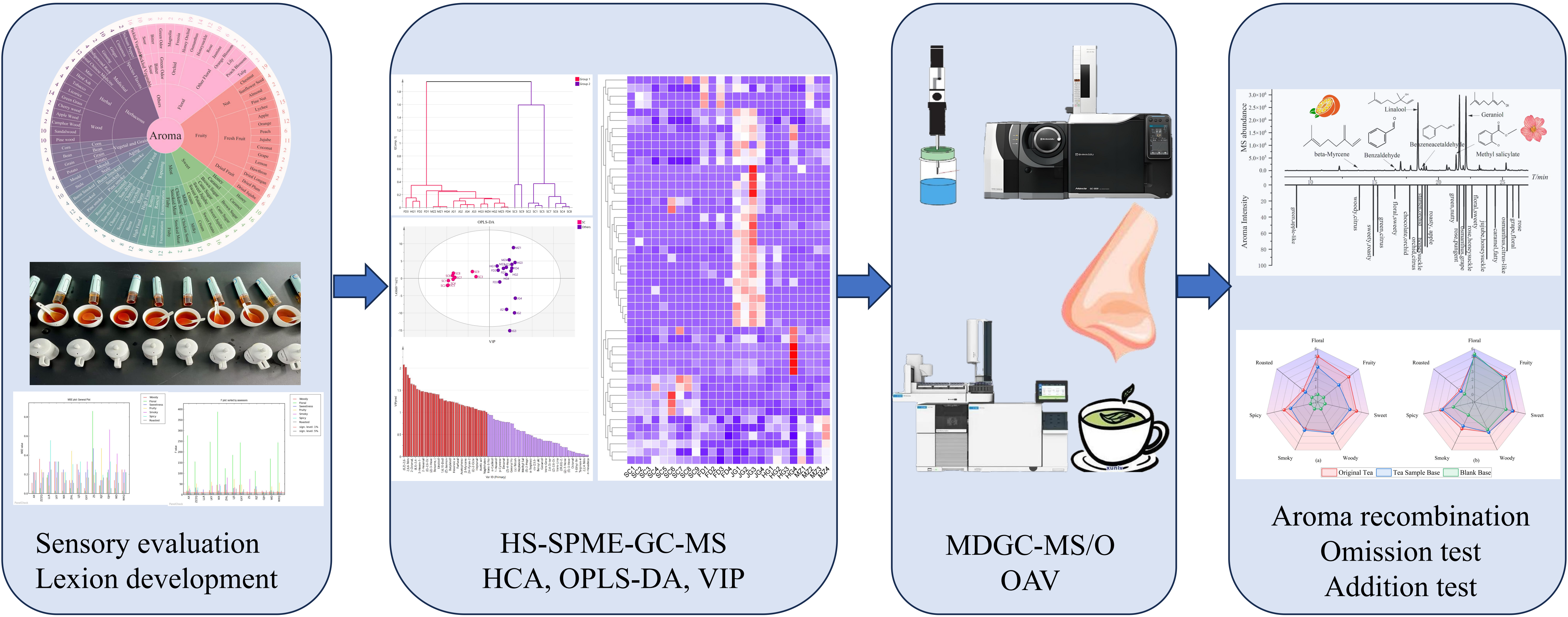

Aroma
Sensory evaluation
Lexion development
HS-SPME-GC-MS
HCA, OPLS-DA, VIP
MDGC-MS/O
OAV
Linalool
Geraniol
beta-Myrcene
Benzaldehyde
Benzeneacetaldehyde
Methyl salicylate
MS abundance
Aroma Intensity
Original Tea
Tea Sample Base
Blank Base
Aroma recombination
Omission test
Addition test
